# When SF_5_ outplays CF_3_: effects of pentafluorosulfanyl decorated scorpionates on copper†

**DOI:** 10.1039/d1sc04846e

**Published:** 2021-10-15

**Authors:** Anurag Noonikara-Poyil, Alvaro Muñoz-Castro, Andrii Boretskyi, Pavel K. Mykhailiuk, H. V. Rasika Dias

**Affiliations:** Department of Chemistry and Biochemistry, The University of Texas at Arlington Arlington TX 76019 USA dias@uta.edu; Grupo de Química Inorgánica y Materiales Moleculares, Facultad de Ingeniería, Universidad Autonoma de Chile El Llano Subercaseaux 2801 Santiago Chile; UORSY, Ukrorgsyntez Ltd PO Box 59 02002 Kyiv Ukraine; Enamine Ltd Chervonotkatska 78 02094 Kyiv Ukraine; Chemistry Department, Taras Shevchenko National University of Kyiv Volodymyrska 64 01601 Kyiv Ukraine Pavel.Mykhailiuk@gmail.com

## Abstract

Polyfluorinated, electron-withdrawing, and sterically demanding supporting ligands are of significant value in chemistry. Here we report the assembly and use of a bis(pyrazolyl)borate, [Ph_2_B(3-(SF_5_)Pz)_2_]^−^ that combines all such features, and involves underutilized pentafluorosulfanyl substituents. The ethylene and carbonyl chemistry of copper(i) supported by [Ph_2_B(3-(SF_5_)Pz)_2_]^−^, a comparison to the trifluoromethylated counterparts involving [Ph_2_B(3-(CF_3_)Pz)_2_]^−^, as well as copper catalyzed cyclopropanation of styrene with ethyl diazoacetate and CF_3_CHN_2_ are presented. The results from cyclopropanation show that SF_5_ groups dramatically improved the yields and stereoselectivity compared to the CF_3_.

## Abstract 2

Copper–ethylene and carbonyl complexes of the newly developed [Ph_2_B(3-(SF_5_)Pz)_2_]^−^ enable the study of ligand steric and electronic effects caused by the –SF_5_ group (dubbed “super CF_3_”), and a comparison to the –CF_3_ bearing analogs.

## Graphical abstract

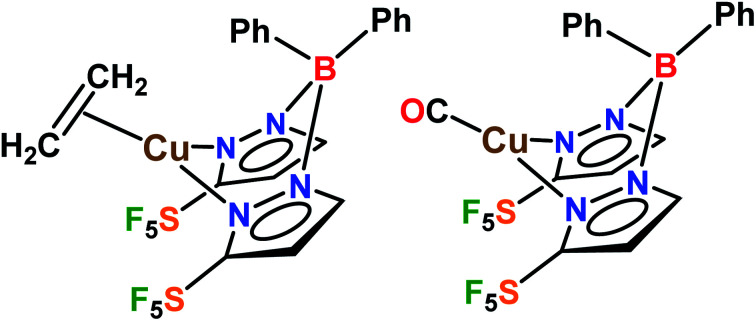

## Introduction

Substituents are the key to modulating the chemical and physical properties of molecules, including those of metal complexes and catalysts. The number of electron-withdrawing substituents that can be utilized for this purpose that are also relatively inert and practical, however, are quite limited. Fluorinated substituents such as the trifluoromethyl (CF_3_) group are especially useful in this regard as they often drastically alter the properties of a molecule compared to their hydrocarbon counterparts.^1^ The pentafluorosulfanyl (SF_5_) is a noticeably underutilized fluorinated substituent compared to the CF_3_ group in chemistry.^2^ It is, however, gaining increasing attention due to its unique and attractive properties including large size (marginally smaller than a *tert*-butyl group), strong electron-withdrawing capabilities, high lipophilicity and excellent chemical and thermal stability, and showing great promise in agrochemical, medicinal and materials chemistry applications.^2,3^ Furthermore, molecules with pentafluorosulfanyl groups are also becoming more accessible *via* effective and convenient routes.^4^ A number of derivatization reactions of SF_5_-group containing molecules are also known.^3*a*,3*b*,4*e*,5^

The metal complexes featuring SF_5_ groups are quite limited,^3*a*,6^ although it was a substituent first introduced in 1960.^7^ Promising outcomes noted in recent reports suggest that pentafluorosulfanyl moiety merits more closer scrutiny and wider utility. For example, recent work by Mecking and co-workers illustrated the benefits of SF_5_ over CF_3_ groups on Ni(ii) salicylaldiminato complexes in ethylene polymerization catalysis (to get more linear and higher molecular weight polymers),^6*a*^ as well as on tetraphenylborate ions in Ni(ii) mediated butadiene polymerizations.^8^ In addition, SF_5_ group has been utilized in luminescent transition metal complexes to minimize the aggregation in the solid-state, improve the solubility, and alter the emission features such as blue shifting of the phosphorescent emissions more significantly relatively to CF_3_ bearing analogs.^2*b*,3*a*,9^

**Fig. 1:**
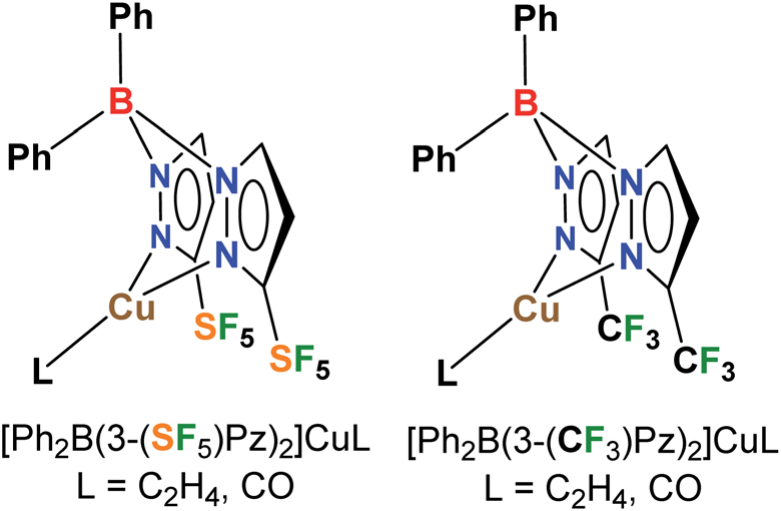
Bis(pyrazolyl)boratocopper(i) complexes decorated with pentafluorosulfanyl (SF_5_) and trifluoromethyl (CF_3_) groups.

Poly(pyrazolyl)borates, commonly referred to as scorpionates,^10^ are very valuable class of ligands in coordination chemistry and catalysis, and form complexes with most metals of the periodic table. Here we report the first metal scorpionates decorated with pentafluorosulfanyl groups. In particular, we describe the synthesis of [Ph_2_B(3-(SF_5_)Pz)_2_]^−^ and the effects of this ligand support on copper(i) as reflected in the structures and bonding of ethylene and CO complexes (which represent two classes of organometallic complexes with significant fundamental and practical significance),^11^ and catalytic alkene cyclopropanation, as well as a direct comparison to the related trifluoromethylated analogs (Fig. 1). It is also notable that there is only an isolated example of a copper complex involving a 4-SF_5_C_6_H_4_-substituted ligand to our knowledge,^12^ whereas CF_3_-bearing ligands with copper are more common and valued in many applications.^11,13^

## Results and discussion

### Synthesis of the SF_5_-pyrazole

**Scheme 1:**
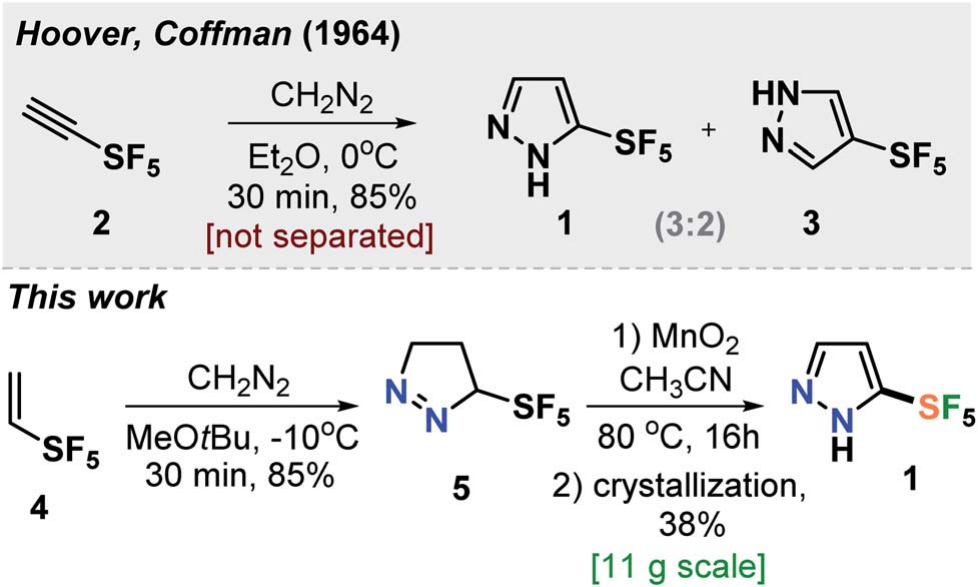
Synthesis of SF_5_-pyrazole **1**.

In 1964, Hoover and Coffman reported that a reaction of alkyne **2** with diazomethane in diethyl ether at 0 °C led to the formation of a mixture of isomeric products **1** and **3** (**3** : **2**) (Scheme 1).^14^ The authors also mentioned that: “these pyrazoles were not separated.” Therefore, we needed to develop a robust practical protocol for the SF_5_-prazole **1**. After some optimization, we found that the reaction of alkene **4** with diazomethane at −10 °C gave pyrazoline **5** in 85% yield. Oxidation of the latter with MnO_2_ followed by crystallization of the resulting material from hexane gave the needed compound SF_5_-pyrazole (**1**) in 38% yield. This product was obtained in 11 g scale in one run (Scheme 1).

### Synthesis of fluorinated scorpionate ligands and copper–ethylene complexes

**Scheme 2:**
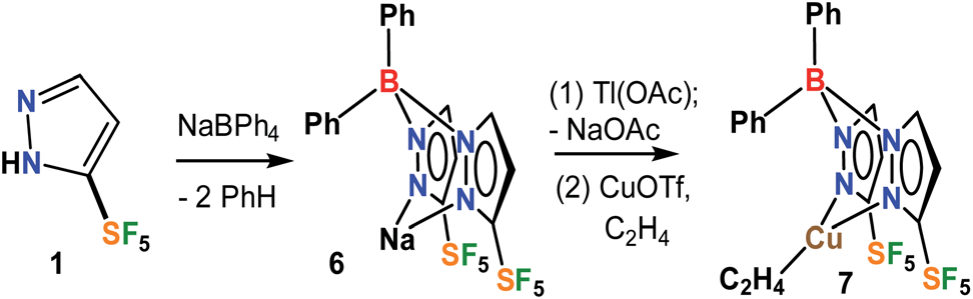
Synthesis of [Ph_2_B(3-(SF_5_)Pz)_2_]Cu(C_2_H_4_).

The fluorinated bis(pyrazolyl)borate [Ph_2_B(3-(SF_5_)Pz)_2_]^−^ ligand possessing SF_5_ groups at the pyrazolyl ring 3-positions was prepared by a reaction of SF_5_-pyrazole (**1**) with NaBPh_4_*via* a benzene elimination pathway (Scheme 2, see ESI† section). This resulting sodium salt was converted to [Ph_2_B(3-(SF_5_)Pz)_2_]Tl (**6**) through metathesis using TlOAc, and utilized in the synthesis of [Ph_2_B(3-(SF_5_)Pz)_2_]Cu(C_2_H_4_) (**7**) by treating with CuOTf and ethylene (Scheme 2). The related copper–ethylene, complex [Ph_2_B(3-(CF_3_)Pz)_2_]Cu(C_2_H_4_) was also synthesized for a comparison. They are colorless crystalline solids, and stable to loss of ethylene in a nitrogen atmosphere at room temperature. The ^19^F NMR spectra of the two adducts are very different due to the unique square pyramidal arrangement of fluorine atoms in SF_5_ moieties *vs.* trigonal pyramidal array in CF_3_ groups, leading to a doublet and a pentet in the former and a singlet in the latter.

The ethylene ^13^C NMR signal in [Ph_2_B(3-(SF_5_)Pz)_2_]Cu(C_2_H_4_) was observed at *δ* = 86.4 ppm. This resonance in [Ph_2_B(3-(CF_3_)Pz)_2_]Cu(C_2_H_4_) was observed at *δ* 82.7 ppm, which is an even larger upfield shift from the free C_2_H_4_ (*δ* 123.1 ppm) signal. Larger upfield shift of the metal bound ethylene ^13^C resonance from the free C_2_H_4_ (*δ* 123.1 ppm) signal has been attributed to the increased shielding resulting from metal-to-ethylene π-back-donation.^15^ Thus [Ph_2_B(3-(SF_5_)Pz)_2_]Cu(C_2_H_4_), with a smaller upfield shift points to relatively lower Cu → ethylene π-backbonding. This is reasonable considering the presence of more electron withdrawing SF_5_ groups (with an estimated electronegativity of 3.65 *vs.* 3.36, and Hammett substituent constant *σ*_m_ of 0.61 *vs.* 0.43 for SF_5_*vs.* CF_3_)^2,16^ on the scorpionate ligand backbone of this copper complex. For comparison, three coordinate [*t*-Bu_2_P(NSiMe_3_)_2_]Cu(C_2_H_4_) with a more strongly backbonding copper site displays its ethylene carbon shift at *δ* 73.0 ppm.^17^ The ^13^C NMR data are particularly useful for such bonding analysis since they are less affected by the ring current effects.

The ethylene protons of [Ph_2_B(3-(R)Pz)_2_]Cu(C_2_H_4_) (R = –SF_5_, –CF_3_) in the ^1^H NMR spectrum appear at *δ* 3.72 and 3.69 ppm, respectively. These protons are most likely affected by the ring currents of flanking phenyl groups sitting over ethylene moieties (see molecular structures below). The presence of additional ethylene in CDCl_3_ solutions at room temperature leads to separate broad signals of free and coordinated ethylene in [Ph_2_B(3-(CF_3_)Pz)_2_]Cu(C_2_H_4_) whereas these signals remain sharp for the –SF_5_ analog **7**, suggesting a quite rapid olefin exchange only in the former at room temperature on the NMR time scale.

### X-ray crystal structures of Cu–ethylene complexes

**Fig. 2:**
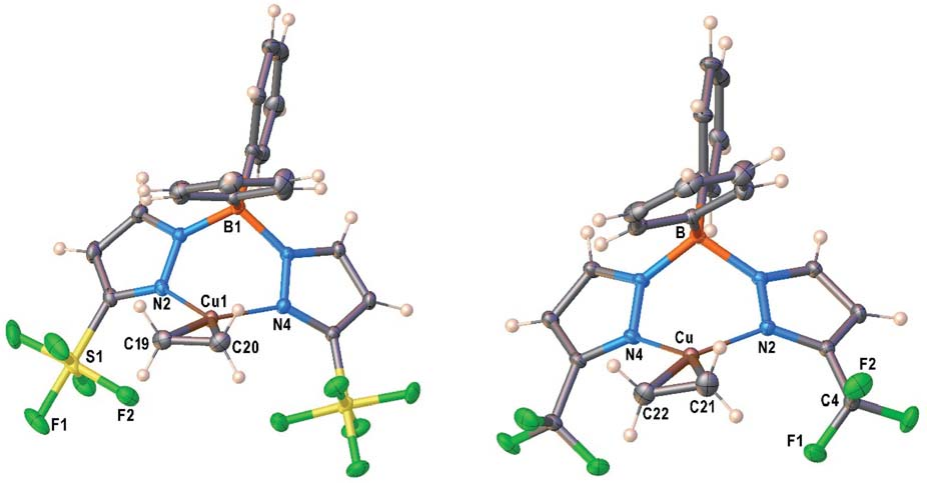
Molecular structures of [Ph_2_B(3-(SF_5_)Pz)_2_]Cu(C_2_H_4_) (**7**) and [Ph_2_B(3-(CF_3_)Pz)_2_]Cu(C_2_H_4_), from left to right.

Molecular structure of [Ph_2_B(3-(R)Pz)_2_]Cu(C_2_H_4_) (R = SF_5_, CF_3_) were unambiguously established by single-crystal X-ray diffraction (Fig. 2). Compound [Ph_2_B(3-(SF_5_)Pz)_2_]Cu(C_2_H_4_) crystallizes with two chemical identical but crystallographically distinct molecules in the asymmetric unit. Selected bond distance and angles are given in Table S3 (ESI†). They are three-coordinate, trigonal planar copper complexes with an η^2^-bound C_2_H_4_ moieties. The bis(pyrazolyl)borate ligands coordinate to copper in κ^2^ fashion *via* nitrogen atoms of two pyrazolyl arms and adopt a boat configuration. One of the phenyl groups on boron sits above the ethylene group. Most of the key features are similar between the two adducts, although the [Ph_2_B(3-(SF_5_)Pz)_2_]Cu(C_2_H_4_) has slightly longer Cu–C and Cu–N distances compared to those of the CF_3_ analog. This could be a result of either greater steric demand or more weakly donating nature of scorpionate in [Ph_2_B(3-(SF_5_)Pz)_2_]Cu(C_2_H_4_).

**Fig. 3:**
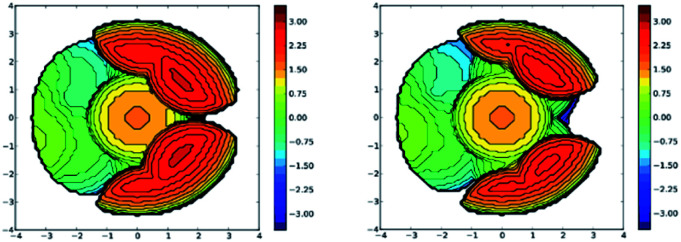
Steric maps of [Ph_2_B(3-(SF_5_)Pz)_2_]Cu (left) and [Ph_2_B(3-(CF_3_)Pz)_2_]Cu (right) moieties based on the calculations using SambVca tool and X-ray data from the ethylene complexes [Ph_2_B(3-(SF_5_)Pz)_2_]Cu(C_2_H_4_) and [Ph_2_B(3-(CF_3_)Pz)_2_]Cu(C_2_H_4_). The resulting % buried volume values are 69.9% (average for the two molecules in the asymmetric unit) and 64.0%, respectively.

Analysis of the topographic steric maps of the two metal complexes using SambVca^18^ and the X-ray crystallographic data indicate percent buried volumes of 69.9% and 64.0% for [Ph_2_B(3-(SF_5_)Pz)_2_]Cu(C_2_H_4_) and [Ph_2_B(3-(CF_3_)Pz)_2_]Cu(C_2_H_4_), respectively (Fig. 3), clearly indicating more protected copper sites in the former as a result of having sterically more demanding SF_5_ groups at the periphery of the coordination pocket. Sluggish ethylene exchange in [Ph_2_B(3-(SF_5_)Pz)_2_]Cu(C_2_H_4_) noted above is probably a result of having greater steric protection at the copper site of this –SF_5_ bearing molecule.

### Computational analysis of copper–ethylene complexes

We have also investigated alkene–copper(i) bonding of [Ph_2_B(3-(SF_5_)Pz)}_2_]Cu(C_2_H_4_), [Ph_2_B(3-(CF_3_)Pz)_2_]Cu(C_2_H_4_), and the hypothetical [Ph_2_B(3-(CH_3_)Pz)_2_]Cu(C_2_H_4_) *via* density functional calculations. The calculated interaction energy (Δ*E*_int_) between the ethylene and Cu(i) center remains similar, ranging from −44.9, −45.9, to −45.2 kcal mol^−1^ (Table 1), respectively, which is further dissected in different contributions within the Ziegler–Rauk energy decomposition analysis (EDA).^19^ It shows that these interactions are primarily electrostatic in nature for all three [Ph_2_B(3-(R)Pz)_2_]Cu(C_2_H_4_) complexes as evident from Δ*E*_elstat_ of about ∼60%, with the remainder consists of ∼36% orbital contributions (Δ*E*_orb_) and ∼4% dispersion-type interactions (Δ*E*_disp_). The Δ*E*_orb_ of [Ph_2_B(3-(SF_5_)Pz)}_2_]Cu(C_2_H_4_) composed of σ-donation and π-backdonation ascribed to the π_1_-C_2_H_4_ → Cu and 
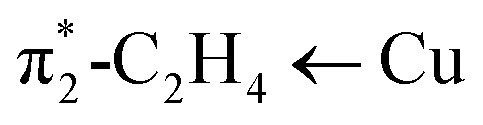
 (Fig. S43, ESI†) in a 29.9% and 54.9% contribution, respectively, which is similar in trend but shows a gradual decrease and an increase in the two components going from –SF_5_ to the –CF_3_ and –CH_3_ analogs, culminating in 24.5% and 63.7%, σ/π-contributions in the most electron rich scorpionate ligand analog [Ph_2_B(3-(CH_3_)Pz)_2_]Cu(C_2_H_4_). These interactions cause a more red-shifted *ῡ*(C=C) as evident from the computed values of 1516.3, 1513.5 and 1509.3 cm^−1^, for [Ph_2_B(3-(R)Pz)_2_]Cu(C_2_H_4_) (R = –SF_5_, –CF_3_, –CH_3_, respectively). These numbers follow the order of Hammett substituent constant *σ*_m_/*σ*_p_ (0.61/0.68, 0.43/0.54, and −0.07/−0.17 for –SF_5_, –CF_3_, –CH_3_, respectively),^2,16^ and are inversely related to the 
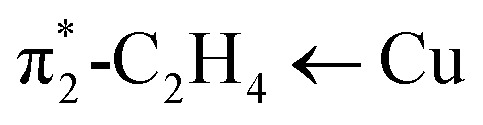
 backbonding contribution (Table 1). This trend is also consistent with computed proton affinities of the [Ph_2_B(3-(R)Pz)_2_]^−^ ligands (and therefore, the donor features of the scorpionate nitrogen sites; see ESI Table S17†), and indicate that [Ph_2_B(3-(SF_5_)Pz)_2_]Cu(C_2_H_4_) features the most weakly donating scorpionate and least backbonding copper site in this series.

**Table 1:** Energy decomposition analyses for the C_2_H_4_–Cu interaction for different [Ph_2_B(3-(R)Pz)_2_]Cu(C_2_H_4_) complexes, with R = –SF_5_, –CF_3_, and –CH_3_. Values in kcal mol^−1^. In addition, π-backbonding and σ-donation components are given as 
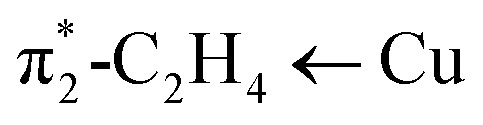
 and π_1_-C_2_H_4_ → Cu, respectively. Calculated *ῡ*(C=C) (in cm^−1^) values are also given

Parameter	[Ph_2_B(3-(SF_5_)Pz)_2_]Cu(C_2_H_4_)		[Ph_2_B(3-(CF_3_)Pz)_2_]Cu(C_2_H_4_)		[Ph_2_B(3-(CH_3_)Pz)_2_]Cu(C_2_H_4_)	
Δ*E*_int_	−44.9		−45.9		−45.2	
Δ*E*_Pauli_	114.0		112.5		125.4	
Δ*E*_disp_	−6.5	4.1%^a^	−5.6	3.5%^a^	−5.3	3.1%^a^
Δ*E*_elstat_	−95.2	59.9%^a^	−95.0	59.9%^a^	−102.1	59.9%^a^
Δ*E*_orb_	−57.2	36.0%^a^	−57.9	36.6%^a^	−63.2	37.1%^a^
π_1_-C_2_H_4_ → Cu	−17.1	29.9%^b^	−16.5	28.5%^b^	−15.5	24.5%^b^
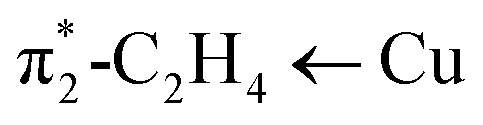	−31.4	54.9%^b^	−33.5	57.9%^b^	−40.2	63.7%^b^
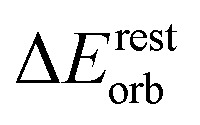	−8.7		−7.8		−7.5	
*ῡ* (C=C) calc.	1516.3		1513.5		1509.3	

a Percentage contribution to the total attractive interactions Δ*E*_elstat_ + Δ*E*_orb_ + Δ*E*_disp_.

b Percentage contribution to the total orbital interactions Δ*E*_orb_.

### Olefin displacement leading to Cu-carbonyl complexes

Some reactivities and catalytic features of [Ph_2_B(3-(SF_5_)Pz)_2_]Cu(C_2_H_4_), [Ph_2_B(3-(CF_3_)Pz)_2_]Cu(C_2_H_4_) have also been investigated. Upon treatment with CO in CH_2_Cl_2_, both adducts afford the corresponding copper carbonyl complexes. They do not lose CO under reduced pressure. The CO stretching frequencies of [Ph_2_B(3-(SF_5_)Pz)_2_]Cu(CO) and [Ph_2_B(3-(CF_3_)Pz)_2_]Cu(CO) were observed at 2121 and 2117 cm^−1^, respectively. For comparison, the *ῡ*(CO) for the highly fluorinated [H_2_B(3,5-(CF_3_)_2_Pz)_2_]Cu(CO)^20^ and relatively electron rich [(Ph_3_B)CH(3,5-(CH_3_)_2_Pz)_2_]Cu(CO)^21^ appear at 2127 and 2092 cm^−1^, respectively. These data indicate that [Ph_2_B(3-(SF_5_)Pz)_2_]Cu(CO) has a notably Lewis acidic copper site, and a relatively weakly donating supporting scorpionate, consistent with the observed carbon chemical shifts and DFT analysis of the corresponding ethylene complex. DFT calculations show that the Cu–CO interaction (ESI†) is slightly less favorable than Cu–C_2_H_4_ (Table 1) in the corresponding [Ph_2_B(3-(R)Pz)_2_]Cu(CO) (Δ*E*_int_ = −39.9 (R = SF_5_), −39.8 (R = CF_3_), and −40.9 kcal mol^−1^ (for hypothetical R = CH_3_)). The thermochemical parameters for the observed C_2_H_4_ → CO replacement in **7** and its –CF_3_ counterpart were also estimated computationally, which show that the free-energy change at room temperature (Δ*G*^298^ K) for these reactions are very small at +1.30 and +0.59 kcal mol^−1^, respectively. They are essentially thermo-neutral processes. Indeed, it is possible to treat CHCl_3_ solutions of [Ph_2_B(3-(SF_5_)Pz)_2_]Cu(CO) and [Ph_2_B(3-(CF_3_)Pz)_2_]Cu(CO) with ethylene at room temperature to re-generate the corresponding ethylene complexes. The Cu–CO bonding features of [Ph_2_B(3-(R)Pz)_2_]Cu(CO) were also investigated using DFT and found to vary systematically along the R = –SF_5_, –CF_3_, and –CH_3_ series, with the lowest 2π* ← Cu backbonding observed for [Ph_2_B(3-(SF_5_)Pz)_2_]Cu(CO), leading to the calculated *ῡ*(CO) of 2110, 2099, and 2080 cm^−1^, respectively (ESI†).

**Fig. 4:**
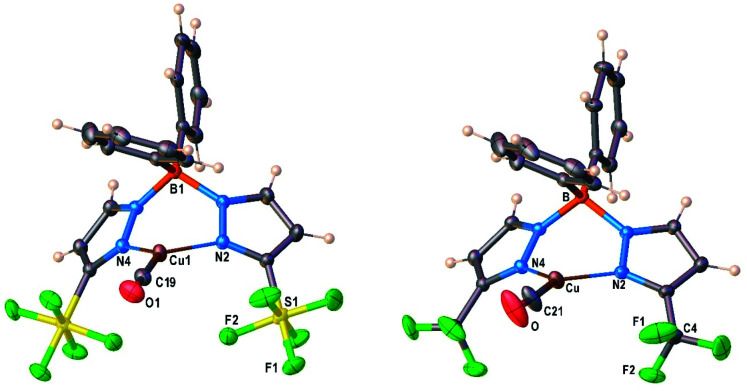
Molecular structures of [Ph_2_B(3-(SF_5_)Pz)_2_]Cu(CO) and [Ph_2_B(3-(CF_3_)Pz)_2_]Cu(CO), from left to right.

Molecular structures of [Ph_2_B(3-(SF_5_)Pz)_2_]Cu(CO), [Ph_2_B(3-(CF_3_)Pz)_2_]Cu(CO) are illustrated in Fig. 4. There are two chemically identical molecules of [Ph_2_B(3-(SF_5_)Pz)_2_]Cu(CO) in its asymmetric unit. Selected bond distances and angles are presented in Table S3 (ESI†). The Cu–CO moieties are essentially linear. The scorpionate coordinates to the metal ion in κ^2^ fashion and adopts a boat configuration. One of the phenyl groups on boron sits above the copper center. The metal to *ipso*-carbon distances are 2.58 and 2.78 Å in [Ph_2_B(3-(R)Pz)_2_]Cu(C_2_H_4_) (R = –SF_5_, –CF_3_), respectively. These separations are within the sum of van der Waals radii of Cu and C (3.10 Å). However, these contacts do not appear to be significant enough to distort the coordination geometry at the metal center because these molecules feature trigonal planar metal sites as evident from the sum of angles at the metal center (∼360°). Furthermore, the *ῡ*(CO) values suggest that the copper sites remain quite Lewis acidic despite the close approach of the phenyl groups. Note that three-coordinate, trigonal planar copper carbonyls are very limited.^22^

Analysis of the topographic steric maps of the two metal complexes using SambVca^18^ and the X-ray crystallographic data indicate percent buried volumes (%*V*_bur_) of 72.8% and 66.3% for [Ph_2_B(3-(SF_5_)Pz)_2_]Cu(CO) and [Ph_2_B(3-(CF_3_)Pz)_2_]Cu(CO), respectively clearly indicting more protected copper sites in the former as a result of having sterically more demanding SF_5_ groups at the periphery of the coordination pocket (Fig. S41, ESI†). These percent buried volume values are larger than those observed for the related ethylene analogs (described above), indicating the adaptability of the scorpionate to accommodate organometallic fragments of different sizes.

### Catalytic activity of copper-complexes

Finally, we have also investigated the catalytic potential of [Ph_2_B(3-(R)Pz)_2_]Cu(C_2_H_4_) (R = –SF_5_, –CF_3_) in cyclopropanation *via* a carbene transfer process. It was found that on reaction of styrene with ethyl diazoacetate (EDA), both copper(i) complexes serve as carbene transfer agents providing the expected cyclopropane as a diastereomeric mixture (Scheme 2). However, [Ph_2_B(3-(SF_5_)Pz)_2_]Cu(C_2_H_4_) gave dramatically higher cyclopropane product yields (99% *vs.* 62%) and greater *cis*-selectivity (3 : 2 *vs.* 1 : 1) compared to the –CF_3_ substituted analog (Scheme 2).

**Scheme 3:**
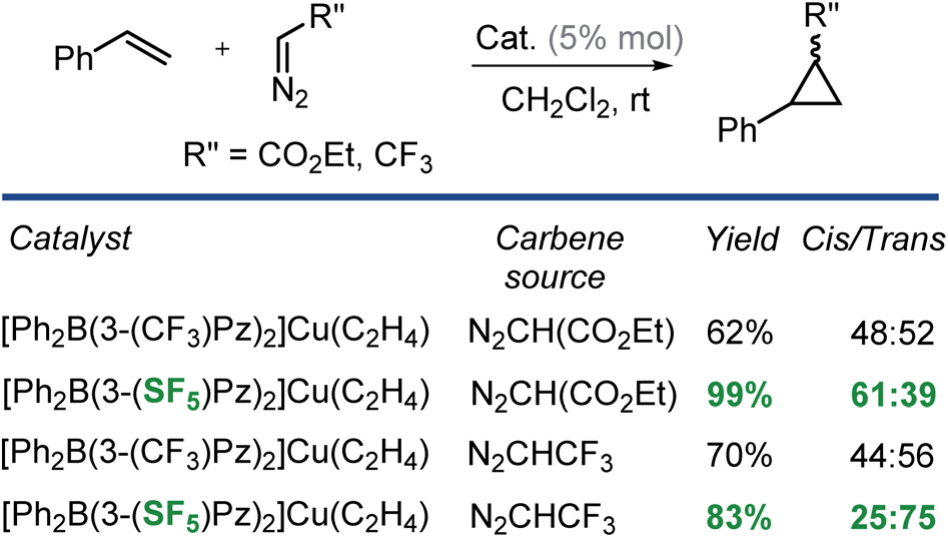
Cyclopropanation of styrene with N_2_CHCO_2_Et (EDA) and CF_3_CHN_2_.

These results are consistent with the previous reports by Perez and co-workers involving tris(pyrazolyl)boratocopper complexes and EDA, which indicate that the higher *cis*-selectivities are associated with bulkier supporting ligands.^23^ Interestingly, when CF_3_CHN_2_ was used as the carbene source,^24^ [Ph_2_B(3-(SF_5_)Pz)_2_]Cu(C_2_H_4_) again gave notably higher product yields than the [Ph_2_B(3-(CF_3_)Pz)_2_]Cu(C_2_H_4_) catalyzed process, but this time, the *trans*-isomer was the major product. It is also known that the *cis*-isomer is the kinetic product while the *trans*-isomer is the thermodynamically favored product.^23^ Therefore, it is possible that the greater steric bulk of the diazo reagent CF_3_CHN_2_ (compared to EDA) favors the latter, causing this interesting reversal in diastereoselectivity. Indeed, Doyle *et al.* has observed high *trans*-selective cyclopropanations in rhodium chemistry with bulky diazo reagents (Scheme 3).^25^

## Conclusions

Overall, we have described the preparation and characterization of the first pentafluorosulfanyl decorated scorpionate [Ph_2_B(3-(SF_5_)Pz)_2_]^−^ and some of its copper chemistry, as well as a new, regioselective route to SF_5_-pyrazole. The [Ph_2_B(3-(SF_5_)Pz)_2_]^−^ is a more sterically demanding and weakly donating ligand compared to the [Ph_2_B(3-(CF_3_)Pz)_2_]^−^, as evident from the copper ethylene and carbonyl chemistry and computational analysis. Moreover, the [Ph_2_B(3-(SF_5_)Pz)_2_]Cu(C_2_H_4_) (**7**) complex displays significantly better efficacy in cyclopropanation of styrene with EDA and CF_3_CHN_2_ compared to that of [Ph_2_B(3-(SF_5_)Pz)_2_]Cu(C_2_H_4_). Fluorinated ligands are important as they often provide metal complexes with certain beneficial features relative to the non-fluorinated, hydrocarbon group bearing ligands. Given the common appearance of CF_3_-ligands in various areas of chemistry,^1*f*^ we believe that with this work, the SF_5_-analogues will also become popular. Further studies on metal complexes supported by SF_5_ containing ligands and practical approaches to other SF_5_-heterocycles are currently underway.

## Data availability

All data associated with this article can be found in the ESI.†

## Author contributions

Conceptualization: HVRD, PM; investigation: AN-P, AM-C, AB; writing and validation: AN-P, AM-C, AB, PM, HVRD; project administration: HVRD.

## Conflicts of interest

There are no conflicts to declare.

## Supplementary Material

SC-012-D1SC04846E-s001

SC-012-D1SC04846E-s002
