# Correction: When SF_5_ outplays CF_3_: effects of pentafluorosulfanyl decorated scorpionates on copper

**DOI:** 10.1039/d3sc90243a

**Published:** 2023-12-22

**Authors:** Anurag Noonikara-Poyil, Alvaro Muñoz-Castro, Andrii Boretskyi, Pavel K. Mykhailiuk, H. V. Rasika Dias

**Affiliations:** a Department of Chemistry and Biochemistry, The University of Texas at Arlington Arlington TX 76019 USA dias@uta.edu; b Grupo de Química Inorgánica y Materiales Moleculares, Facultad de Ingeniería, Universidad Autonoma de Chile El Llano Subercaseaux 2801 Santiago Chile; c UORSY, Ukrorgsyntez Ltd PO Box 59 02002 Kyiv Ukraine; d Enamine Ltd Chervonotkatska 78 02094 Kyiv Ukraine; e Chemistry Department, Taras Shevchenko National University of Kyiv Volodymyrska 64 01601 Kyiv Ukraine Pavel.Mykhailiuk@gmail.com

## Abstract

Correction for ‘When SF_5_ outplays CF_3_: effects of pentafluorosulfanyl decorated scorpionates on copper’ by Anurag Noonikara-Poyil *et al.*, *Chem. Sci.*, 2021, **12**, 14618–14623, https://doi.org/10.1039/D1SC04846E.

The authors regret that in the original version of the manuscript, an inadvertent error was made during percent buried volume calculation and topographical steric map creation of bis(pyrazolyl)borate supporting ligands of [Ph_2_B(3-(SF_5_)Pz)_2_]Cu(C_2_H_4_), [Ph_2_B(3-(CF_3_)Pz)_2_]Cu(C_2_H_4_), [Ph_2_B(3-(SF_5_)Pz)_2_]Cu(CO) and [Ph_2_B(3-(CF_3_)Pz)_2_]Cu(CO) using SambVca. This was caused due to the removal of only ethylene and CO groups, rather than the entire Cu–ethylene or Cu–CO moiety (including the metal atom) as suggested by SambVca protocol. Correct percent buried volumes (%*V*_bur_) pertinent to the results and discussion section are given below.

**Table:** 

Compound	%*V*_bur_
[Ph_2_B(3-(SF_5_)Pz)_2_]Cu(C_2_H_4_)	65.6
[Ph_2_B(3-(CF_3_)Pz)_2_]Cu(C_2_H_4_)	59.5
[Ph_2_B(3-(SF_5_)Pz)_2_]Cu(CO)	68.7
[Ph_2_B(3-(CF_3_)Pz)_2_]Cu(CO)	60.8

The corrected versions of Fig. 3, S40 and S41 can be found below. The electronic supplementary information (ESI) available online has now been updated to reflect these changes.


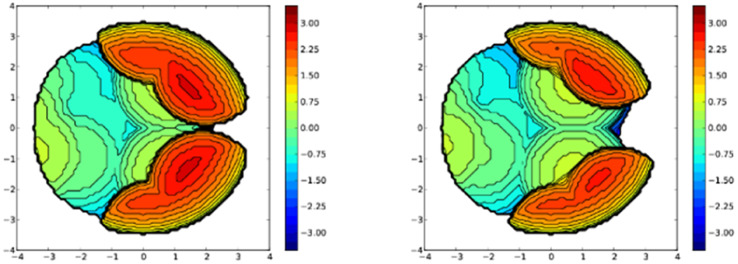
 **Fig. 3** Steric maps of bis(pyrazolyl)borate ligands in [Ph_2_B(3-(SF_5_)Pz)_2_]Cu(C_2_H_4_) (left) and [Ph_2_B(3-(CF_3_)Pz)_2_]Cu(C_2_H_4_) (right). The related % buried volume (%*V*_bur_) values are 65.6 (average for the two molecules in the asymmetric unit) and 59.5, respectively.


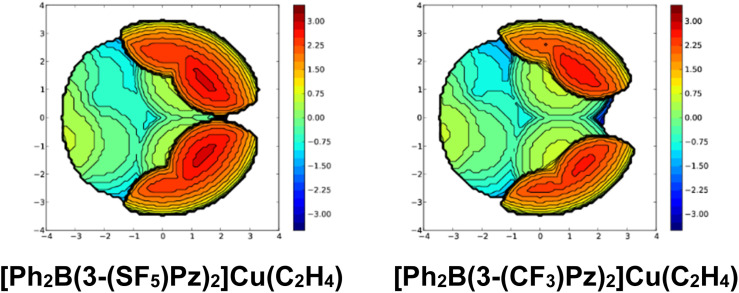
 **Fig. S40** Steric maps of bis(pyrazolyl)borate ligands in [Ph_2_B(3-(SF_5_)Pz)_2_]Cu(C_2_H_4_) (left) and [Ph_2_B(3-(CF_3_)Pz)_2_]Cu(C_2_H_4_) (right) based on X-ray data. The resulting % buried volume (%*V*_bur_) values are 65.6 (average of 66.1 and 65.0 for the two molecules in the asymmetric unit) and 59.5, respectively.


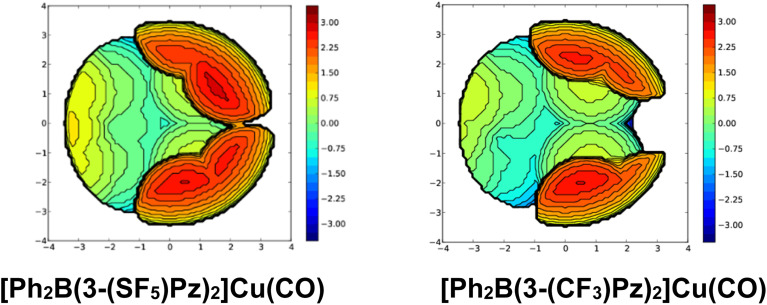
 **Fig. S41** Steric maps of bis(pyrazolyl)borate ligands in [Ph_2_B(3-(SF_5_)Pz)_2_]Cu(CO) (left) and [Ph_2_B(3-(CF_3_)Pz)_2_]Cu(CO) (right) based on X-ray data. The resulting % buried volume (%*V*_bur_) values are 68.7 (average of 69.0 and 68.4 for the two molecules in the asymmetric unit) and 60.8 (fluorine atoms of one of the CF_3_ groups of [Ph_2_B(3-(CF_3_)Pz)_2_]Cu(CO) show positional disorder, and only the major occupancy fluorine atoms were utilized in the calculation to avoid inflated steric bulk), respectively. For comparison, inclusion of both disorder-parts in [Ph_2_B(3-(CF_3_)Pz)_2_]Cu(CO) leads to a marginally higher %*V*_bur_ value of 61.9.

The percent buried volumes (%*V*_bur_) and the steric maps were computed using SambVca 2.1 (https://www.aocdweb.com/OMtools/sambvca2.1/) for a sphere radius of 3.5 Å about the metal center, Bondi van der Waals radii scaled by a factor of 1.17, 0.10 Å mesh spacing, and including hydrogen atoms. The %*V*_bur_ values for [Ph_2_B(3-(SF_5_)Pz)_2_]Cu(C_2_H_4_) and [Ph_2_B(3-(SF_5_)Pz)_2_]Cu(CO) are the average of two molecules of each type in the asymmetric unit.

The Royal Society of Chemistry apologises for these errors and any consequent inconvenience to authors and readers.

## Supplementary Material

SC-015-D3SC90243A-s001

