# Supplementary material for: Correction: When SF5 outplays CF3: effects of pentafluorosulfanyl decorated scorpionates on copper
Source: Chem Sci. 2023 Dec 22;15(3):1159–61. doi: 10.1039/d3sc90243a (PMC10793599; doi:10.1039/d3sc90243a)
Supplement: SC-015-D3SC90243A-s001 [file SC-015-D3SC90243A-s001.pdf]

Electronic Supplementary Material (ESI) for Chemical Science.
This journal is © The Royal Society of Chemistry 2023

Supporting Information

When SF_5 outplays CF_3: Effects of pentafluorosulfanyl decorated scorpionates on copper

Anurag Noonikara-Poyil,[1] Alvaro Muños-Castro,[2] Andrii Boretskyi,[3]

Pavel K. Mykhailiuk,[4,5],* H. V. Rasika Dias [1],*

Affiliations:

[1] Department of Chemistry and Biochemistry, The University of Texas at Arlington, Arlington, Texas 76019, USA

E-mail: dias@uta.edu.

[2] Grupo de Química Inorgánica y Materiales Moleculares, Facultad de Ingeniería, Universidad Autonoma de Chile, El Llano Subercaseaux 2801, Santiago, Chile

[3] UORSY, Ukrorgsyntez Ltd, PO Box 59, 02002 Kyiv, Ukraine

[4] Enamine Ltd., Chervonotkatska 78, 02094 Kyiv, Ukraine

[5] Chemistry Department, Taras Shevchenko National University of Kyiv Volodymyrska 64, 01601 Kyiv, Ukraine

E-mail: Pavel.Mykhailiuk@gmail.com.

Table of Contents

General Information S-2
Table for spectroscopic comparison S-3
Synthesis and characterization of Compounds S-4
Cyclopropanation S-11
^{1}H, ^{13}C, ^{19}F NMR and Raman Spectra of Compounds S-12
X-ray Data Collection and Structure Determinations S-30
Steric Maps S-54
Computational studies S-56
References S-64

General Information

Solvents were purchased from commercial sources, purified before use. NMR spectra were recorded at 25 °C on a JEOL Eclipse 500 and JEOL Eclipse 400 spectrometers (^{1}H, 500.16 and 399.78 MHz ^{13}C, 125.78 and 100.52 MHz, and ^{19}F, 470.62 and 376.17 MHz) or a Varian 400 MHz spectrometer (^{1}H, 399.97 MHz ^{13}C, 100.61 MHz, and ^{19}F, 376.50 MHz). ^{1}H and ^{13}C NMR spectra are referenced to the solvent peak (^{1}H; $CDCl_3$ δ 7.26, $(CD_3)_2SO$ δ 2.50, $(CD_3)_2CO$ δ 2.05 ^{13}C; $CDCl_3$ δ 77.16, $(CD_3)_2SO$ δ 39.52, $(CD_3)_2CO$ δ 29.8). ^{1}H NMR coupling constants (J) are reported in Hertz (Hz) and multiplicities are indicated as follows: s (singlet), d (doublet), t (triplet), pent (pentet), m (multiplet), dd (doublet of doublet), pd (pentet of doublet). ^{19}F NMR values were referenced to external $CFCl_3$. Melting points were obtained on a Mel-Temp II apparatus and were not corrected. Elemental analyses were performed using a Perkin-Elmer Model 2400 CHN analyzer. IR spectra were collected at room temperature on a Shimadzu IR Prestige-21 FTIR containing an ATR attachment using pure liquid or solid materials, with instrument resolution at 2 cm^{-1}. Raman data were collected on a Thermo Scientific DXR3 Raman microscope with a HeNe laser source of 633 nm, by placing pure solid materials on a glass slide. 2-Diazo-1,1,1-trifluoroethane was synthesized as previously reported.[1] All other reactants and reagents were purchased from commercial sources or obtained as noted below. Heating was accomplished by either a heating mantle or a silicone oil bath.

CAUTION: Thallium compounds are toxic. Care must be taken when manipulating thallium containing materials.

Table S1. Selected NMR and vibrational spectroscopic data of ethylene and carbon monoxide complexes.

	Compound	Raman/IR (cm^{-1}) (C=C/C≡O)	^{1}H NMR (ppm) ($C_2\underline{H}_4$)	$^{13}C\{^{1}H\}$ NMR (ppm) (C=C/ C≡O)	Reference
1	$[Ph_2B(3\text{-}(SF_5)Pz)_2]Cu(C_2H_4)$		3.72	86.4	This work
2	$[Ph_2B(3\text{-}(CF_3)Pz)_2]Cu(C_2H_4)$		3.69	82.7	This work
3	$[Ph_2B(3\text{-}(SF_5)Pz)_2]Cu(CO)$	2121	-	170.6	This work
4	$[Ph_2B(3\text{-}(CF_3)Pz)_2]Cu(CO)$	2117	-	171.3	This work
5	Free CO	2143	-	184.0	[2]

Synthesis and Characterization of Compounds

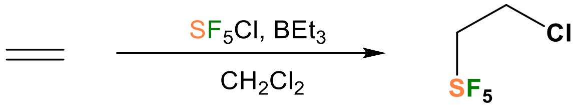

(2-Chloroethyl)pentafluoro-λ⁶-sulfane: A 3-necked 2 L reactor was charged with 500 mL of CH_2Cl_2 and cooled to -90 °C. Then ethylene (~ 20 g, 0.714 mol, 1.2 equiv) and SF_5Cl (95 g, 0.585 mol, 1.0 equiv; obtained according to the previous report[3]) were condensed to the reactor. Et_3B (20 mL, 1.0 M in hexanes) was added at -90 °C, and the mixture was allowed to slowly warm up by passing a weak current of ethylene through the solution. When the temperature reached -50 °C, an additional portion of Et_3B (10 mL, 1.0 M in hexanes) was added, and the current of ethylene was stopped. The mixture was allowed to warm to room temperature. The solution was washed with cold water (with ice), dried, and distilled at atmospheric pressure using a rectification column. The product was obtained with impurities of methylene chloride and 1,2-dichloroethane. Yield: 130 g of crude, ~ 50% purity, 58%, colorless oil. ^{1}H NMR (500 MHz, $CDCl_3$): δ (ppm) 5.37 – 5.24 (m, 2H), 4.01 – 3.98 (m, 2H). ^{19}F{^{1}H} NMR (470 MHz, $CDCl_3$): δ (ppm) 81.8 (pent, $^2J_{FF}$ = 147 Hz, 1F), 65.3 (d, $^2J_{FF}$ = 146, 4F).

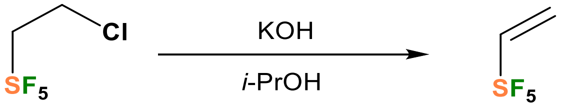

Pentafluoro(vinyl)-λ⁶-sulfane (4): A 500 mL round bottom flask was charged with a solution of 40% KOH (100 mL) and a solution of crude (2-chloroethyl)pentafluoro-λ⁶-sulfane (40 g) in 60 mL of isopropanol. A distillation column was attached to the flask, and the mixture was heated up to boiling (~ 60 °C oil bath temperature). As the product was distilled off, the temperature was increased to 100 °C. The distillate can be used for the next step, despite the possible impurity of methylene. If water gets into the product during distillation, additional washing with water and drying will be needed. Yield: 25 g of crude, ~ 70% purity, 99%, colorless oil. ^{1}H NMR (400 MHz, $CDCl_3$): δ (ppm) 6.77 – 6.59 (m, 1H), 6.02 (d, J = 16.0 Hz, 1H), 5.84 – 5.68 (m, 1H). ^{19}F{^{1}H} NMR (376 MHz, $CDCl_3$): δ (ppm) 81.2 (pent, $^2J_{FF}$ = 151 Hz, 1F), 59.6 (d, $^2J_{FF}$ = 150 Hz, 4F).

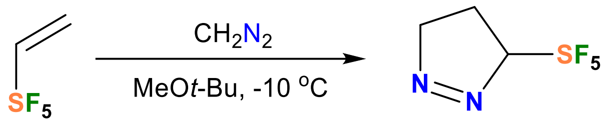

5-(Pentafluoro-λ^6-sulfanyl)-4,5-dihydro-3*H*-pyrazole (5): Crude pentafluoro(vinyl)-λ^6-sulfane (30 g, 0.136 mol, 1.0 equiv) was dissolved in MTBE (150 mL) and a solution of diazomethane (freshly prepared, 6.3 g, 0.15 mol, 1.1 equiv) in MTBE (50 mL) was added at -10 °C. The color of diazomethane disappeared after 30 min, and the mixture was warmed up to a room temperature. The mixture was concentrated under reduced pressure. Yield: 22.7 g, 85%, yellow oil (ca. 80% purity). ^{1}H NMR (500 MHz, $CDCl_3$): δ 7.34 (br, SC*H*N is overlapped with $CHCl_3$ signal), 4.63 (d, *J* = 11.8 Hz, 1H), 4.54 (d, *J* = 11.8 Hz, 1H), 4.39 – 4.29 (m, 1H), 3.70 – 3.55 (m, 1H).^{19}F{^{1}H} NMR (376 MHz, $CDCl_3$): δ (ppm) 79.2 (pent, $^2J_{FF}$ = 148 Hz, 1F), 57.6 (d, $^2J_{FF}$ = 147 Hz, 4F).

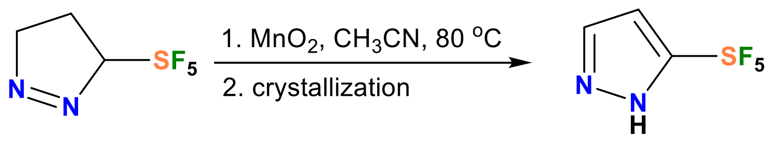

5-(Pentafluoro-λ^6-sulfanyl)-1*H*-pyrazole (1): To a solution of 5-(pentafluoro-λ^6-sulfanyl)-4,5-dihydro-3*H*-pyrazole (30 g, 0.15 mol, 1.0 equiv) in CH_3CN (500 mL) was added MnO_2 (60 g, 0.69 mol, 4.6 equiv). The mixture was heated to 80 °C in oil bath with a thermocouple and stirred at this temperature for 16 h. The mixture was filtered through a thick layer of silica gel. The filtrate was concentrated under reduced pressure and dissolved in a 1M NaOH solution (150 mL), then the mixture was acidified with 3M HCl. The product was extracted with $CHCl_3$ (3 × 100 mL), concentrated, recrystallized from hexane and dried. Yield: 11.5 g, 38%, white solid, m.p. = 115-116 °C. ^{1}H NMR (400 MHz, $CDCl_3$): δ (ppm) 12.04 (br s, 1H, N*H*), 7.70 (d, $^3J_{HH}$ = 1.0 Hz, 1H, C*H*N), 6.68 (d, $^3J_{HH}$ = 2.5 Hz, 1H, C*H*C). ^{13}C{^{1}H} NMR (100 MHz, $CDCl_3$): δ (ppm) 159.8 (pent, $^2J_{FC}$ = 25 Hz, *C*SF_5), 130.4 (*C*HN), 104.1 (br m, *C*HC). ^{19}F{^{1}H} NMR (376 MHz, $CDCl_3$): δ (ppm) 79.9 (pent, $^2J_{FF}$ = 152 Hz, 1F), 63.5 (d, $^2J_{FF}$ = 152 Hz, 4F). LCMS $(M+H)^+$: 195. HRMS (ESI-TOF) *m/z*: $[M + H]^+$ calcd for $C_3H_4F_5N_2S$ 195.0015; found 195.0002.

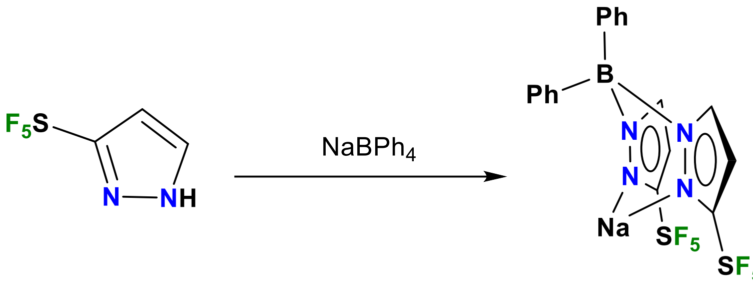

[$Ph_2B(3-(SF_5)Pz)_2$]Na: 5-(Pentafluoro-λ^6--sulfanyl)-1*H*-pyrazole (**1**) (0.20 g, 1.03 mmol) and $NaBPh_4$ (0.12 g, 0.34 mmol) were mixed in a high pressure tube and heated at 180 °C for 4 h. The reaction mixture was cooled to room temperature and washed with hexanes (3 X 5 mL). The solid was dried under vacuum to get pale orange solid. Yield: 50%. M.P.: 195-198 °C (decomposition). ^{1}H NMR ($(CD_3)_2CO$): δ (ppm) 7.93 (br, 2H, Pz*H*), 7.79-7.30 (m, 6H, Ph*H*), 7.17-7.05 (m, 4H, Ph*H*), 6.70 (d, *J* = 2.3 Hz, 2H, Pz*H*). ^{19}F NMR ($(CD_3)_2CO$): δ (ppm) 81.4 (pent, $^2J_{F\text{-}F}$ = 154.8 Hz, 2F), 63.0 (d, $^2J_{F\text{-}F}$ = 154.8 Hz, 8F). ^{13}C{^{1}H} NMR ($(CD_3)_2CO$): δ (ppm) 160.5 (br, C-3), 135.6, 134.9, 133.1, 131.8, 131.1, 128.0, 127.3, 103.9 (C-4).

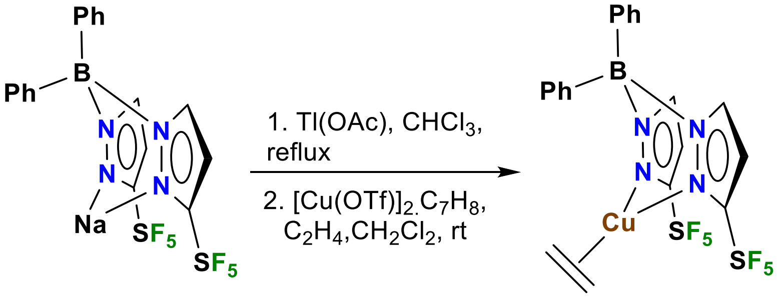

[$Ph_2B(3-(SF_5)Pz)_2$]Cu(C_2H_4): [$Ph_2B(3-(SF_5)Pz)_2$]Na (0.10 g, 0.17 mmol) and Tl(OAc) (0.05 g, 0.19 mmol) were dissolved 5 mL of $CHCl_3$ in a Schlenk flask and refluxed for 1 h. The reaction mixture was cooled to room temperature and filtered through a pad of celite in a frit funnel. The solvent was evaporated to get the crude product. This crude product is suitable for the synthesis of copper complex. It is possible to remove the impurity, presumably the unreacted sodium salt, by passing the dichloromethane solution of crude material through an alumina column. Dichloromethane from the eluent was evaporated to obtain cleaner the thallium adduct, [$Ph_2B(3-(SF_5)Pz)_2$]Tl as white powder. Yield: 72%, ^{1}H NMR ($CDCl_3$): δ (ppm) 7.72 (br,

2H, Pz*H*), 7.41-7.35 (m, 6H, Ph*H*), 7.06 (br, 4H, Ph*H*), 6.48 (d, J = 2.3 Hz, 2H, Pz*H*). ^{19}F NMR ($CDCl_3$): δ (ppm) 82.8 (pd, $^{2}J_{F\text{-}F}$ = 154 Hz, $^{4}J_{Tl\text{-}F}$ = 75.6 Hz, 2F), 65.5 (dd, $^{4}J_{Tl\text{-}F}$ = 777.3 Hz, $^{2}J_{F\text{-}F}$ = 154 Hz, 8F). ^{13}C{^{1}H} NMR ($CDCl_3$): δ (ppm) 159.2 (br, C-3), 148.3 (br), 138.2, 135.1, 128.7, 128.2, 102.2 (C-4). [$Ph_2B(3\text{-}(SF_5)Pz)_2$]Tl and [Cu(OTf)]$_2$•$C_7H_8$ (0.05 g, 0.09 mmol) were taken in a 50 mL Schlenk flask and 10 mL ethylene-saturated CH_2Cl_2 was added into it. The reaction mixture was stirred for 3 h at room temperature. Ethylene gas was bubbled for three times during the reaction (30 seconds each time). The reaction mixture was filtered through a pad celite in a frit funnel. The filtrate was concentrated with continuous flow of ethylene and kept at -20 °C to obtain X-ray quality colorless crystals of [$Ph_2B(3\text{-}(SF_5)Pz)_2$]Cu($C_2H_4$). Yield: 89%. M.P.: 110-113 °C (decomposition). Anal. Calc. $C_{20}H_{18}BCuF_{10}N_4S_2$: C, 37.37; H, 2.82%; N, 8.72%. Found: C, 36.99%; H, 2.68%; N, 8.34%. ^{1}H NMR ($CDCl_3$): δ (ppm) 7.63 (br, 2H, Pz*H*), 7.35-7.28 (m, 5H, Ph*H*), 7.24-7.23 (m, 3H, Ph*H*), 6.64-6.62 (m, 2H, Ph*H*), 6.54 (d, J = 2.9 Hz, 2H, Pz*H*), 3.72 (s, 4H, C_2H_4). ^{19}F NMR ($CDCl_3$): δ (ppm) 80.8 (pent, $^{2}J_{F\text{-}F}$ = 153 Hz, 2F), 65.4 (d, $^{2}J_{F\text{-}F}$ = 153 Hz, 8F). ^{13}C{^{1}H} NMR ($CDCl_3$): δ (ppm) 159.4 (br, C-3), 137.7 (C-2), 136.1, 133.9, 128.1, 127.9, 127.7, 103.5 (C-4), 86.4 (C=C). IR (cm^{-1}): 3010, 2933, 2862, 2360, 2337, 1539, 1497, 1432, 1389, 1305, 1273, 1195, 1149, 1077, 984, 977, 892, 849, 815. Raman (cm^{-1}): 3161, 3152, 3137, 3055, 3044, 2996, 1593, 1568, 1538, 1371, 1315, 1278, 1273, 1232, 1188, 1156, 1142, 1078, 1072, 1031, 1017, 1000, 981, 959, 833, 827.

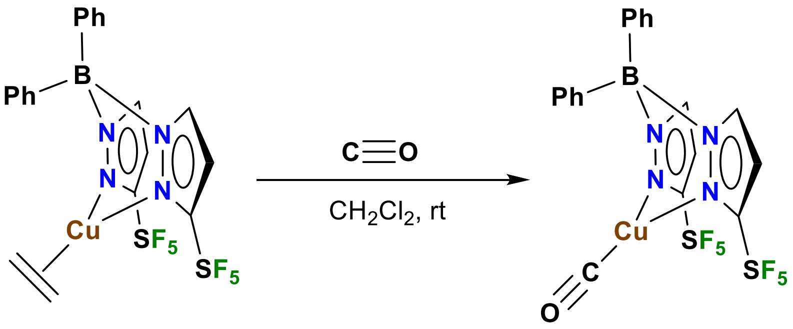

[$Ph_2B(3\text{-}(SF_5)Pz)_2$]Cu(CO): [$Ph_2B(3\text{-}(SF_5)Pz)_2$]Cu($C_2H_4$) (0.03 g, 0.05 mmol) was dissolved in 3 mL CH_2Cl_2 and stirred for ~1-2 min while bubbling carbon monoxide. The reaction mixture was concentrated with continuous flow of carbon monoxide and kept at -20 °C to obtain X-ray quality colorless crystals of [$Ph_2B(3\text{-}(SF_5)Pz)_2$]Cu(CO). Yield: 98%. M.P.: 105-107 °C (decomposition). ^{1}H NMR ($CDCl_3$): δ (ppm) 7.64 (br, 2H, Pz*H*), 7.37-7.34 (m, 7H, Ph*H*), 6.96 (br, 3H, Ph*H*), 6.53 (d, J = 1.7

Hz, 2H, Pz*H*). ^{19}F NMR ($CDCl_3$): δ (ppm) 80.2 (pent, $^{2}J_{F\text{-}F}$ = 154.8 Hz, 2F), 65.1 (d, $^{2}J_{F\text{-}F}$ = 154.8 Hz, 8F). $^{13}C\{^{1}H\}$ NMR ($CDCl_3$): δ (ppm) 170.6 (br, CO), 159.0(br, C-3), 143.1 (br), 137.2, 135.0, 128.3, 128.1, 103.5 (C-4). IR (cm^{-1}): 3024, 2967, 2121 ($\bar{\upsilon}_{C\equiv O}$), 1498, 1445, 1431, 1393, 1322, 1306, 1264, 1198, 1157, 1143, 1077, 987, 979, 849, 814.

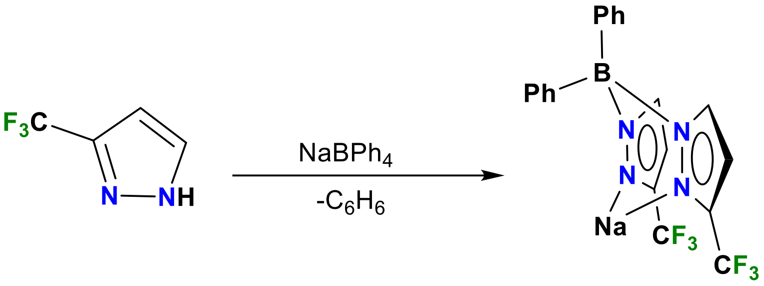

[Ph₂B(3-(CF₃)Pz)₂]Na: The 3-(CF_3)PzH (2.00 g, 14.70 mmol) and $NaBPh_4$ (1.70 g, 4.90 mmol) were mixed in a 25 mL long Schlenk flask and heated at 150 ºC for 4 h during which the benzene was removed using a short path distillation apparatus. The reaction mixture was cooled to room temperature and washed with hexanes (3 X 15 mL). The remaining solid was dried under vacuum to get pure product as white solid. Yield: 52%. M.P.: 215-218 °C (decomposition). Anal. Calc. $C_{20}H_{14}BF_6N_4Na$: C, 52.43; H, 3.08%; N, 12.23%. Found: C, 52.80%; H, 3.46%; N, 11.90%. ^{1}H NMR ($(CD_3)_2SO$): δ (ppm) 7.10-7.03 (m, 12H, Pz*H* & Ph*H*), 6.42 (d, *J* = 1.72 Hz, 2H, Pz*H*). ^{19}F NMR ($(CD_3)_2SO$): δ (ppm) -59.0 (s). $^{13}C\{^{1}H\}$ NMR ($(CD_3)_2SO$): δ (ppm) 151.6, 140.4 (q, $^{2}J_{C\text{-}F}$ = 36.0 Hz, C-3/C-5), 135.6, 133.7, 126.1, 124.8, 122.9 (q, $^{1}J_{C\text{-}F}$ =267.5 Hz, CF_3), 101.4 (C-4). IR (cm^{-1}): 3117, 3060, 1515, 1496, 1434, 1369, 1259, 1165, 1125, 1113, 1060, 1007, 970, 883, 845, 814.

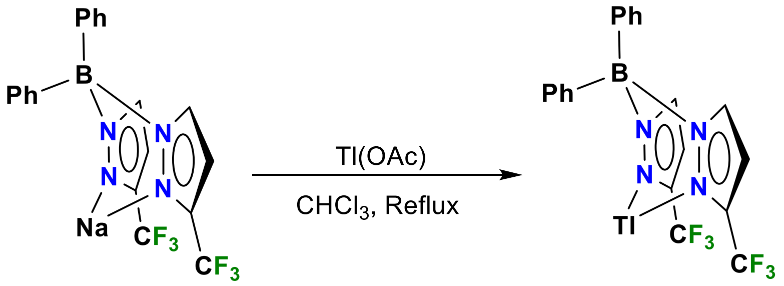

[Ph₂B(3-(CF₃)Pz)₂]Tl: [$Ph_2B(3\text{-}(CF_3)Pz)_2$]Na (1.00 g, 2.18 mmol) and Tl(OAc) (0.58 g, 2.28 mM) were dissolved 30 mL of $CHCl_3$ in a Schlenk flask and refluxed for 1 h. The reaction mixture was cooled to room temperature and filtered through a pad

of celite in a frit funnel. The solvent was evaporated to get the product as white powder. Yield: 88%. M.P.: 194-197 °C (decomposition). Anal. Calc. $C_{20}H_{14}BF_6N_4Tl$: C, 37.56; H, 2.21%; N, 8.76%. Found: C, 37.21%; H, 2.18%; N, 8.48%. ^{1}H NMR ($CDCl_3$): δ (ppm) 7.74 (d, J = 1.2 Hz, 2H, Pz*H*), 7.38-7.32 (m, 6H, Ph*H*), 7.01 (br, 4H, Ph*H*), 6.49 (s, 1H, Pz*H*). ^{19}F NMR ($CDCl_3$): δ (ppm) -60.2 (d, $^4J_{Tl\text{-}F}$ = 575.8 Hz). ^{13}C{^{1}H} NMR ($CDCl_3$): δ (ppm) 148.9, 143.0 (q, $^2J_{C\text{-}F}$ = 43.2 Hz, C-3/C-5), 138.9, 135.0, 128.5, 127.9, 122.2 (q, $^1J_{C\text{-}F}$ =268.7 Hz, CF_3), 102.7 (C-4). IR (cm^{-1}): 2945, 1558, 1431, 1364, 1274, 1260, 1236, 1150, 1126, 1115, 1052, 1037, 999, 974.

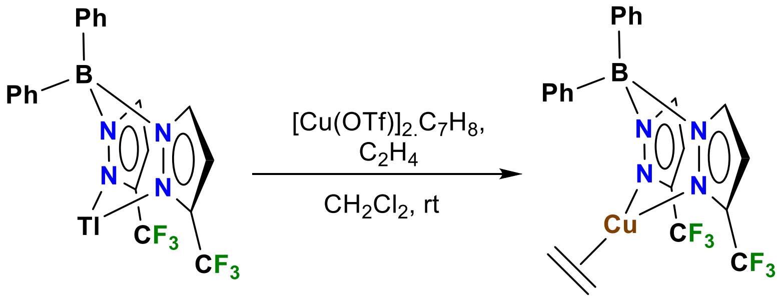

[$Ph_2B(3\text{-}(CF_3)Pz)_2$]Cu($C_2H_4$): [$Ph_2B(3\text{-}(CF_3)Pz)_2$]Tl (0.10 g, 0.16 mmol) and [Cu(OTf)]$_2$.C_7H_8 (0.05 g, 0.09 mmol) were taken in a 50 mL Schlenk flask and 10 mL ethylene-saturated CH_2Cl_2 was added into it. The reaction mixture was stirred for 3 h at room temperature. Ethylene gas was bubbled for three times during the reaction (30 seconds each time). The reaction mixture was filtered through a pad celite in a frit funnel. The filtrate was concentrated with continuous flow of ethylene and kept at -20 °C to obtain X-ray quality colorless crystals of [$Ph_2B(3\text{-}(CF_3)Pz)_2$]Cu($C_2H_4$). Yield: 91%. M.P.: 115-117 °C (decomposition). Anal. Calc. $C_{22}H_{18}BCuF_8N_4$: C, 50.16; H, 3.44%; N, 10.64%. Found: C, 49.81%; H, 3.25%; N, 10.29%. ^{1}H NMR ($CDCl_3$): δ (ppm) 7.65 (s, 2H, Pz*H*), 7.29 (br, 6H, Ph*H*), 6.95 (br, 4H, Ph*H*), 6.54 (s, 2H, Pz*H*), 3.69 (s, 4H, C_2H_4). ^{19}F NMR ($CDCl_3$): δ (ppm) -60.6 (s). ^{13}C{^{1}H} NMR ($CDCl_3$): δ (ppm) 142.7 (q, $^2J_{C\text{-}F}$ = 37.2 Hz, C-3/C-5), 138.0, 134.9 (br), 127.6, 120.9 (q, $^1J_{C\text{-}F}$ =268.7 Hz, CF_3), 103.7 (C-4), 82.7 (C=C). IR (cm^{-1}): 3009, 2928, 2851, 1531, 1524, 1495, 1433, 1424, 1371, 1274, 1268, 1258, 1194, 1184, 1166, 1159, 1128, 1077, 1012, 976, 956, 944, 891. Raman (cm^{-1}): 3173, 3157, 3152, 3138, 3067, 3052, 2983, 1593, 1569, 1531, 1521, 1388, 1381, 1371, 1269, 1185, 1168, 1159, 1144, 1083, 1032, 1011, 1000, 974, 944, 836.

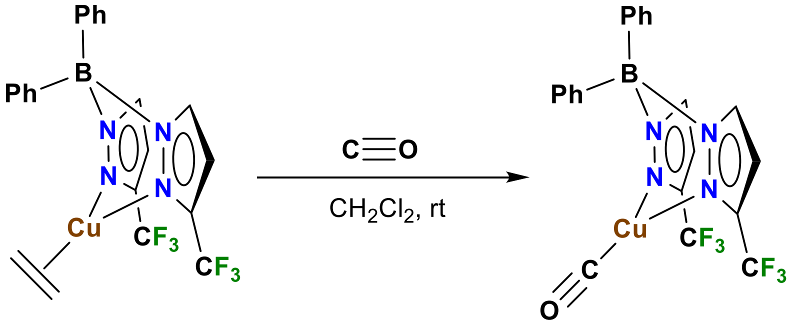

[$Ph_2B(3\text{-}(CF_3)Pz)_2$]Cu(CO): [$Ph_2B(3\text{-}(CF_3)Pz)_2$]Cu($C_2H_4$) (0.10 g, 0.05 mmol) was dissolved in 8 mL CH_2Cl_2 and stirred for ~3-5 min while bubbling carbon monoxide. The reaction mixture was concentrated with continuous flow of carbon monoxide and kept at -20 °C to obtain X-ray quality colorless crystals of [$Ph_2B(3\text{-}(CF_3)Pz)_2$]Cu(CO) Yield: 99%. M.P.: 115-117 °C (decomposition). Anal. Calc. $C_{21}H_{14}BCuF_6N_4O$: C, 47.89; H, 2.68%; N, 10.64%. Found: C, 47.58%; H, 2.55%; N, 10.48%. ^1H NMR ($CDCl_3$): δ (ppm) 7.63 (d, J = 1.2 Hz, 2H, Pz*H*), 7.34-7.29 (m, 6H, Ph*H*), 6.90-6.89 (m, 4H, Ph*H*), 6.54 (d, J = 2.3 Hz, 2H, Pz*H*). ^{19}F NMR ($CDCl_3$): δ (ppm) -61.2 (s). $^{13}C\{^1H\}$ NMR ($CDCl_3$): δ (ppm) 171.3 (C≡O), 145.1, 142.7 (q, $^2J_{C\text{-}F}$ = 37.2 Hz, C-3/C-5), 138.1, 134.5, 127.9, 120.9 (q, $^1J_{C\text{-}F}$ =269.9 Hz, CF_3), 103.6 (C-4). IR (cm^{-1}): 3011, 2932, 2854, 2117 ($\bar{\upsilon}_{C\equiv O}$), 1524, 1491, 1433, 1370, 1275, 1259, 1199, 1166, 1160, 1135, 1083, 1076, 1012, 893, 835.

Cyclopropanation

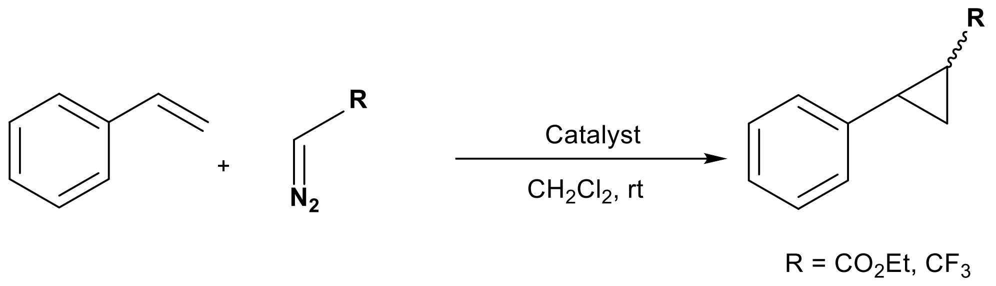

General method for the cyclopropanation: A Schlenk flask was charged with the styrene (2.8 mmol, 10.0 equiv.), catalyst (5 mol%) and dichloromethane (5.0 mL) under a nitrogen atmosphere. A dichloromethane solution of diazocompound (0.28 m mol) was added to the reaction via a syringe pump over 5 h. Following the complete addition of the diazocompound, the react ion mixture was stirred for 5 h. NMR yield was calculated using 1,3,5-tris(trifluoromethyl)benzene (^{1}H NMR was used to calculate the yield of ethyldiazoacetate reactions (ester CH_2 peak) and ^{19}F NMR was used to calculate the yield of 2-diazo-l,l,l-trifluoroethane reactions (CF_3 peak)).

Table S2. Cyclopropanation

Entry	Carbene source	Catalyst	Cyclopropane Yield (%)	Cis/trans	Reference to products
1	$N_2CH(CO_2Et)$	$[Ph_2B(3\text{-}(CF_3)Pz)_2]Cu(C_2H_4)$	62	48:52	[4]
2	$N_2CH(CO_2Et)$	$[Ph_2B(3\text{-}(SF_5)Pz)_2]Cu(C_2H_4)$	99	61:39	[4]
3	N_2CHCF_3	$[Ph_2B(3\text{-}(CF_3)Pz)_2]Cu(C_2H_4)$	70	44:56	[5]
4	N_2CHCF_3	$[Ph_2B(3\text{-}(SF_5)Pz)_2]Cu(C_2H_4)$	83	25:75	[5]

^{1}H, ^{13}C, ^{19}F NMR and Raman Spectra of Compounds

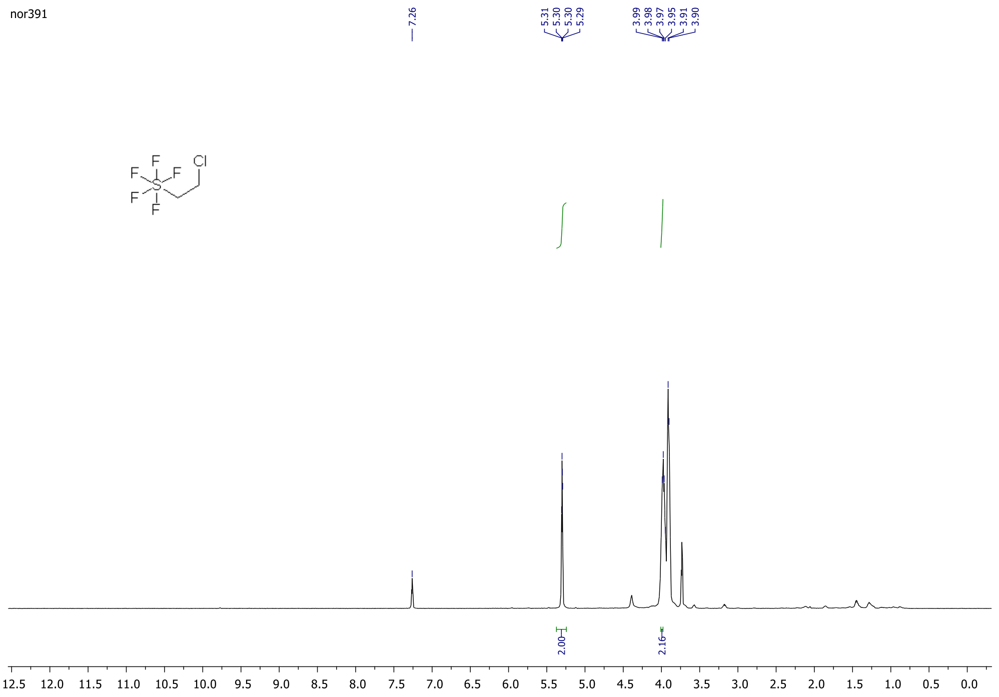

Figure S1: ^{1}H NMR Spectrum of **(2-Chloroethyl)pentafluoro-λ^{6}-sulfane** in $CDCl_3$.

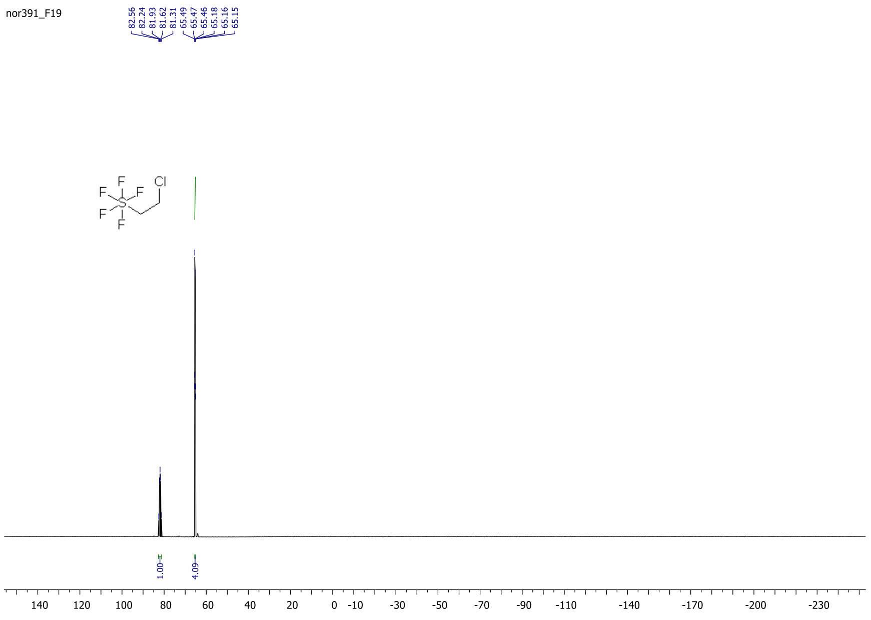

Figure S2: ^{19}F NMR Spectrum of **(2-Chloroethyl)pentafluoro-λ^{6}-sulfane** in $CDCl_3$.

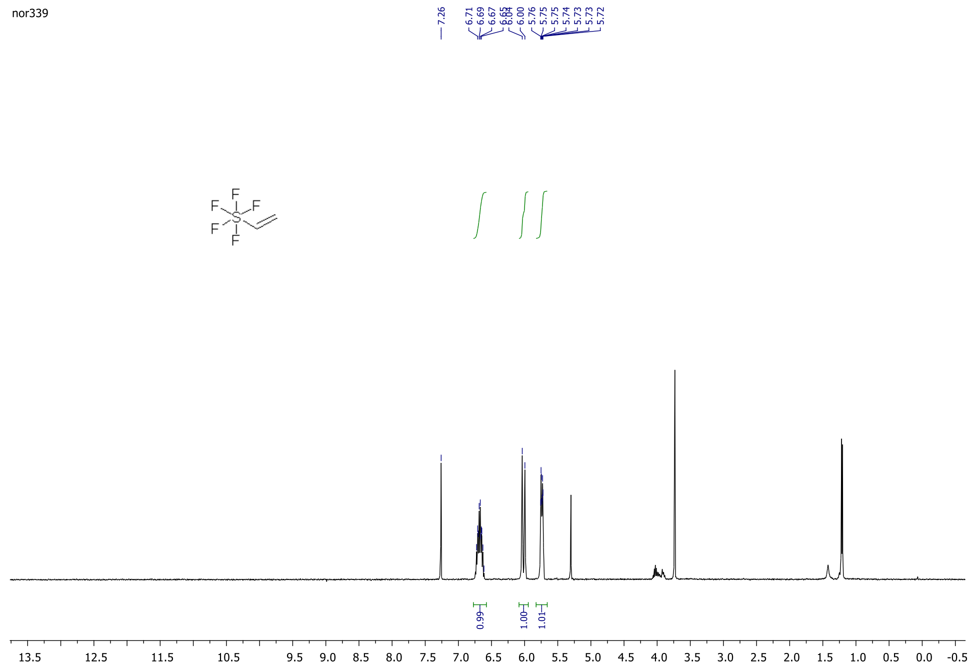

Figure S3: ^{1}H NMR Spectrum of **Pentafluoro(vinyl)-λ⁶-sulfane (4)** in $CDCl_3$.

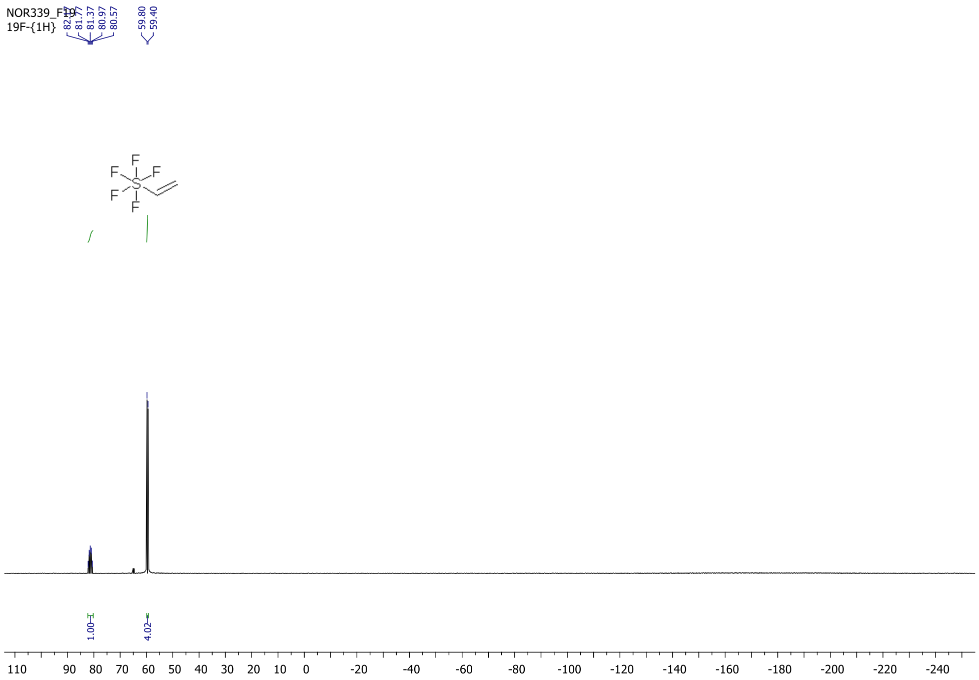

Figure S4: ^{19}F NMR Spectrum of **Pentafluoro(vinyl)-λ⁶-sulfane (4)** in $CDCl_3$.

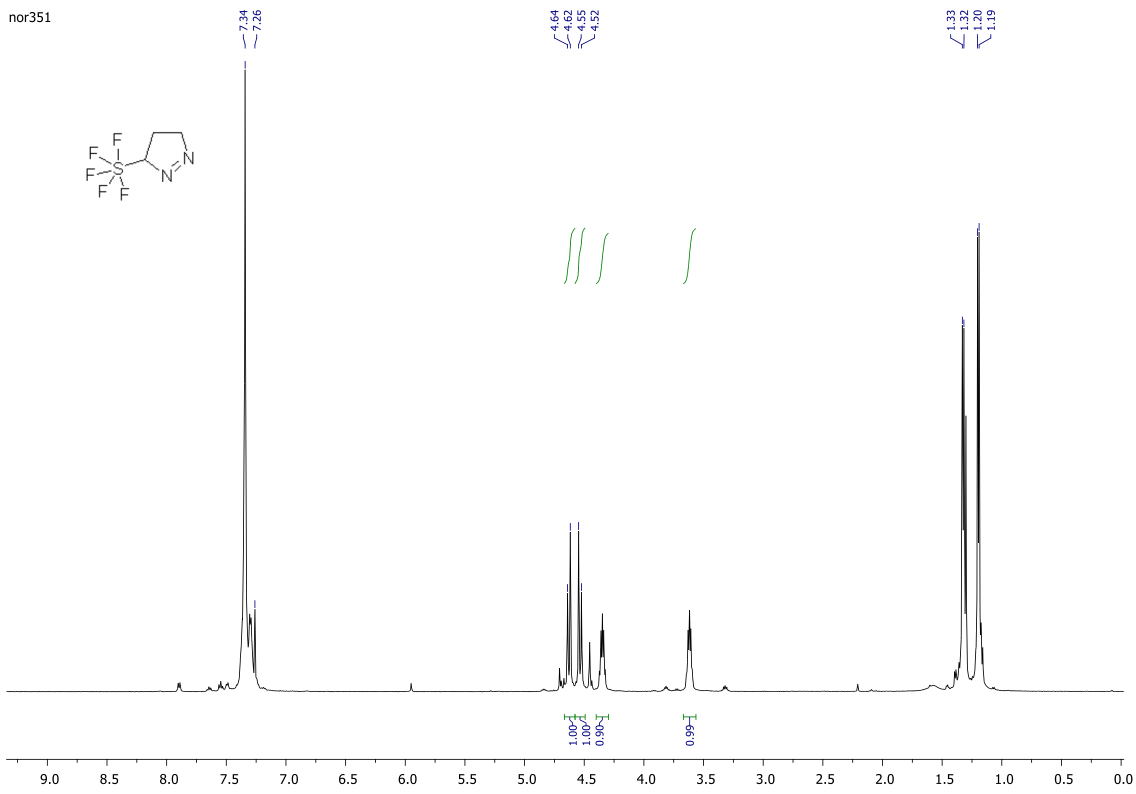

Figure S5: ^{1}H NMR Spectrum of **5-(Pentafluoro-λ^{6}-sulfanyl)-4,5-dihydro-3*H*-pyrazole (5)** in $CDCl_3$.

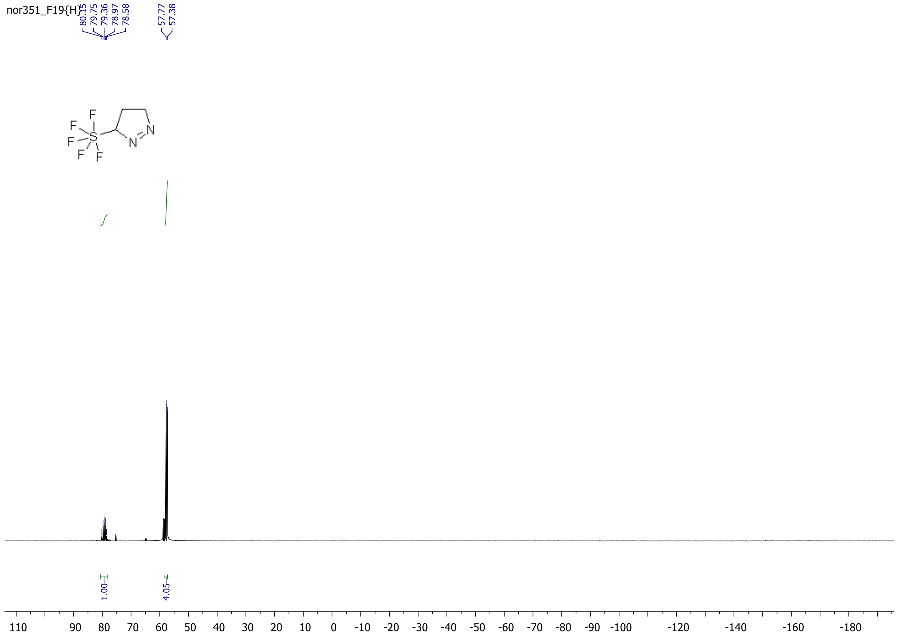

Figure S6: ^{19}F NMR Spectrum of **5-(Pentafluoro-λ^{6}-sulfanyl)-4,5-dihydro-3*H*-pyrazole (5)** in $CDCl_3$.

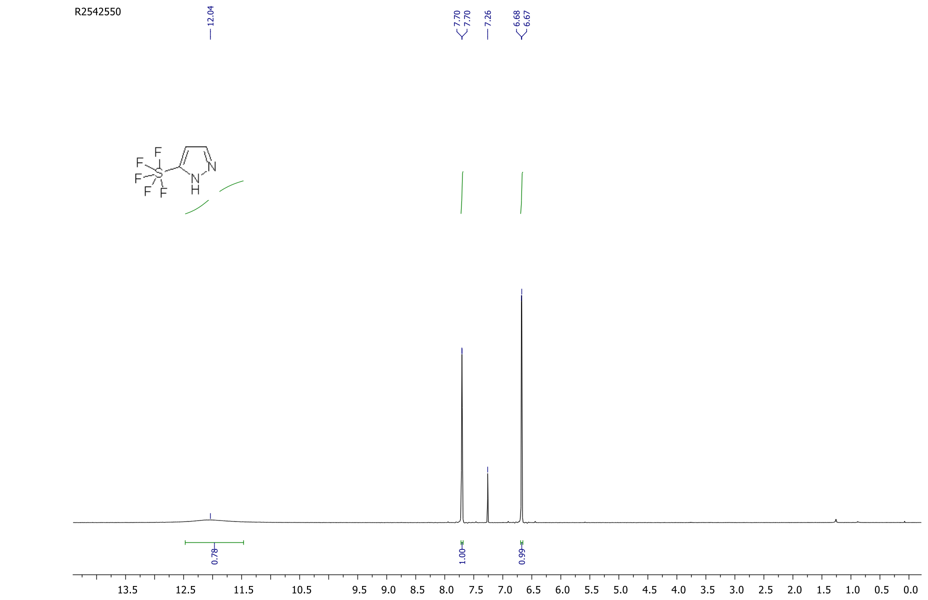

Figure S7: ^{1}H NMR Spectrum of **5-(Pentafluoro-λ^{6}-sulfanyl)-1*H*-pyrazole (1)** in $CDCl_3$.

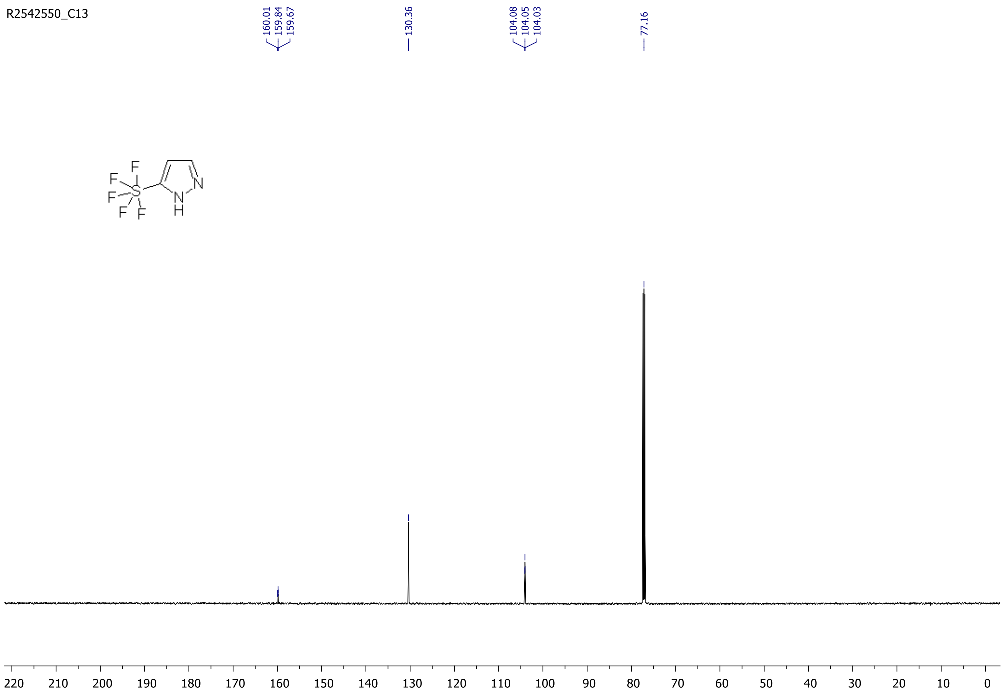

Figure S8: ^{13}C NMR Spectrum of **5-(Pentafluoro-λ^{6}-sulfanyl)-1*H*-pyrazole (1)** in $CDCl_3$.

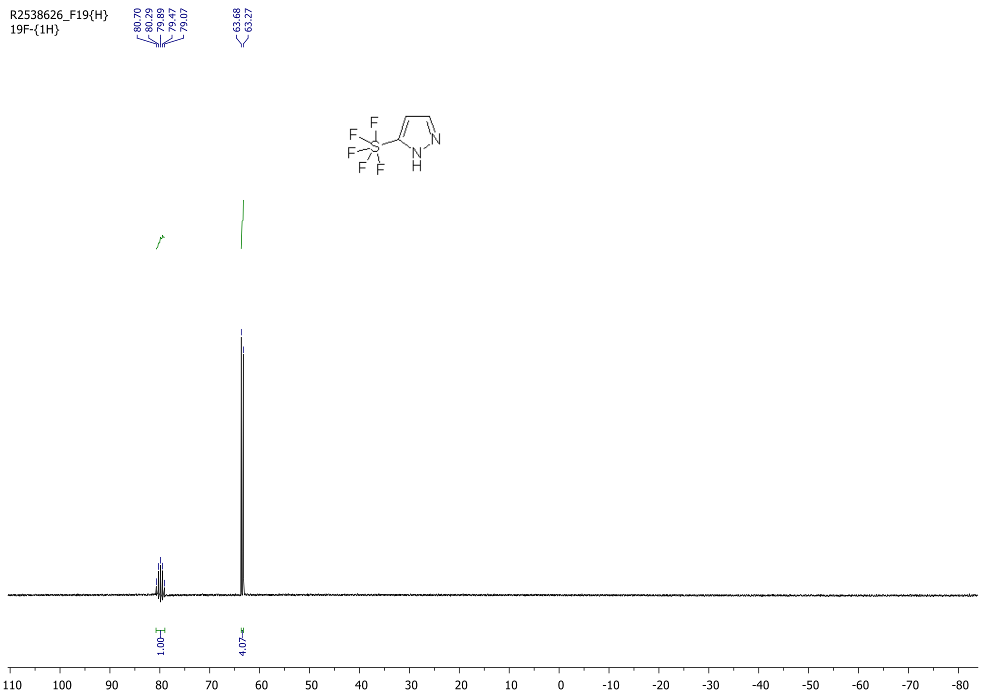

Figure S9: ^{19}F NMR Spectrum of **5-(Pentafluoro-λ^6-sulfanyl)-1*H*-pyrazole (1)** in $CDCl_3$.

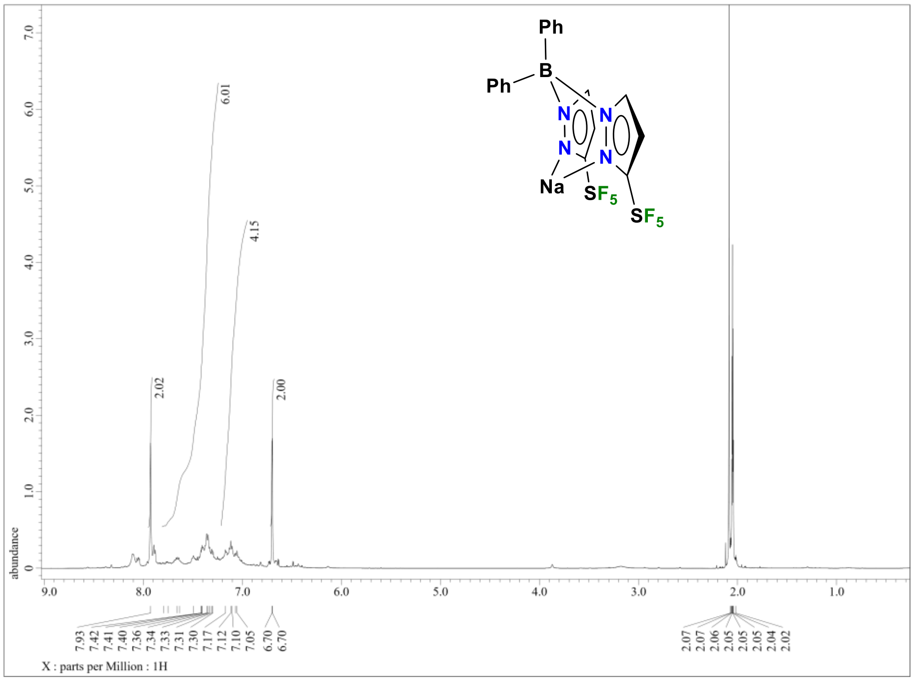

Figure S10: ^{1}H NMR Spectrum of **[$Ph_2B(3\text{-}(SF_5)Pz)_2$]Na** in $(CD_3)_2CO$.

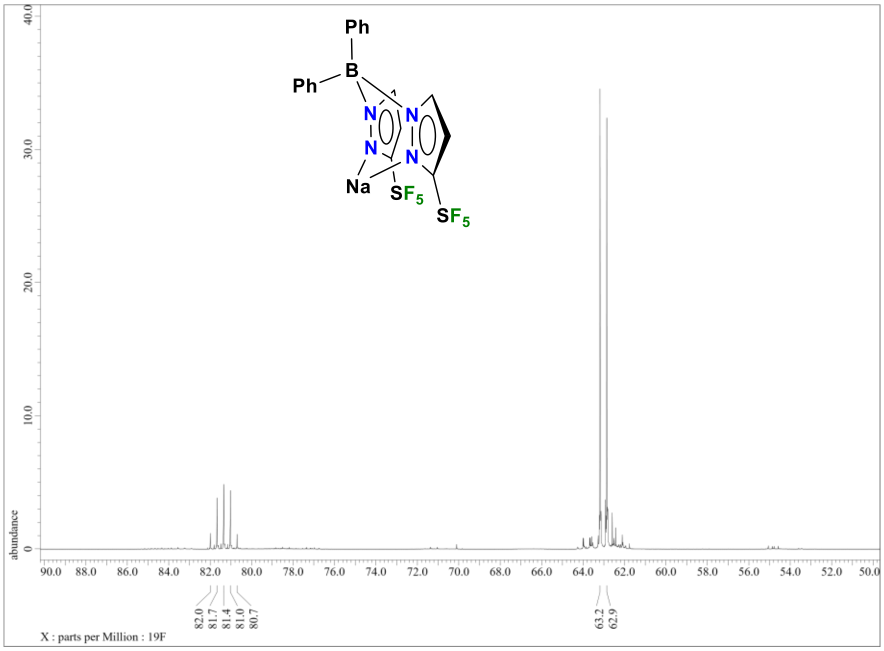

Figure S11: ^{19}F NMR Spectrum of **[Ph$_2$B(3-(SF$_5$)Pz)$_2$]Na** in $(CD_3)_2CO$.

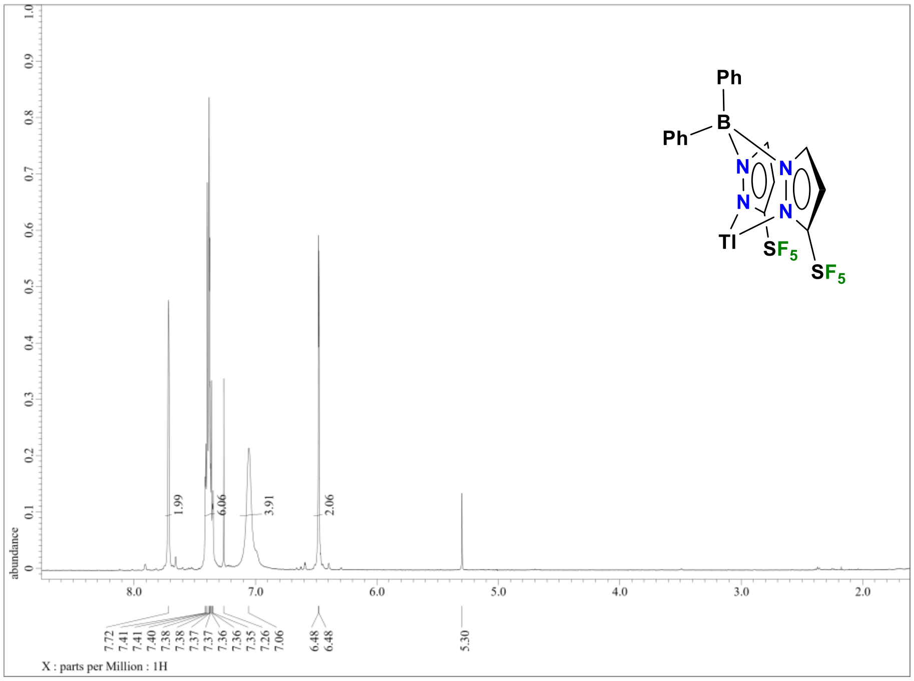

Figure S12: ^{1}H NMR Spectrum of **[Ph$_2$B(3-(SF$_5$)Pz)$_2$]Tl** in $CDCl_3$.

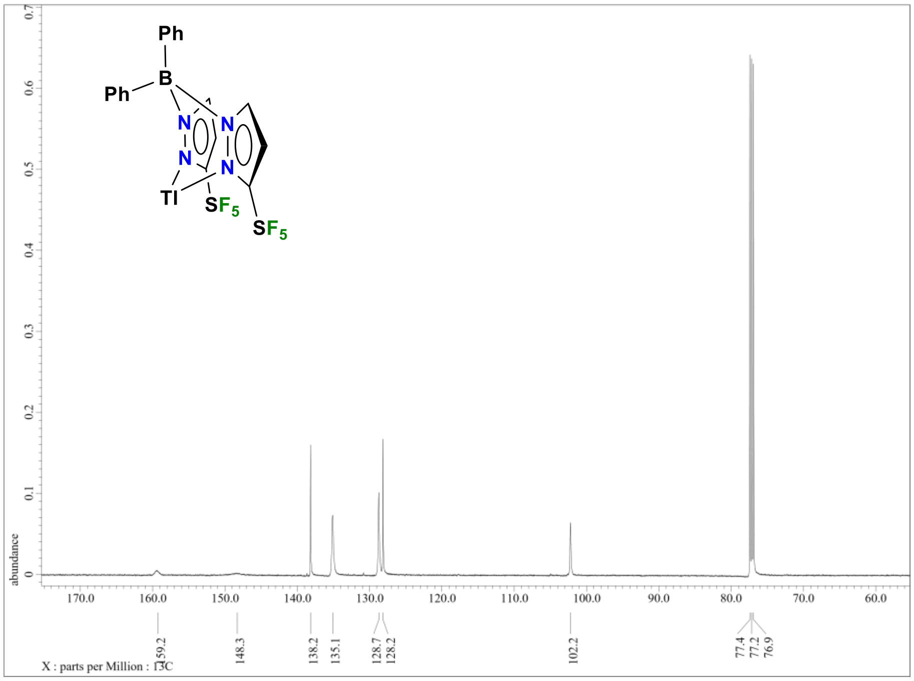

Figure S13: ^{13}C NMR Spectrum of **$Ph_2B(3-(SF_5)Pz)_2]Tl$** in $CDCl_3$.

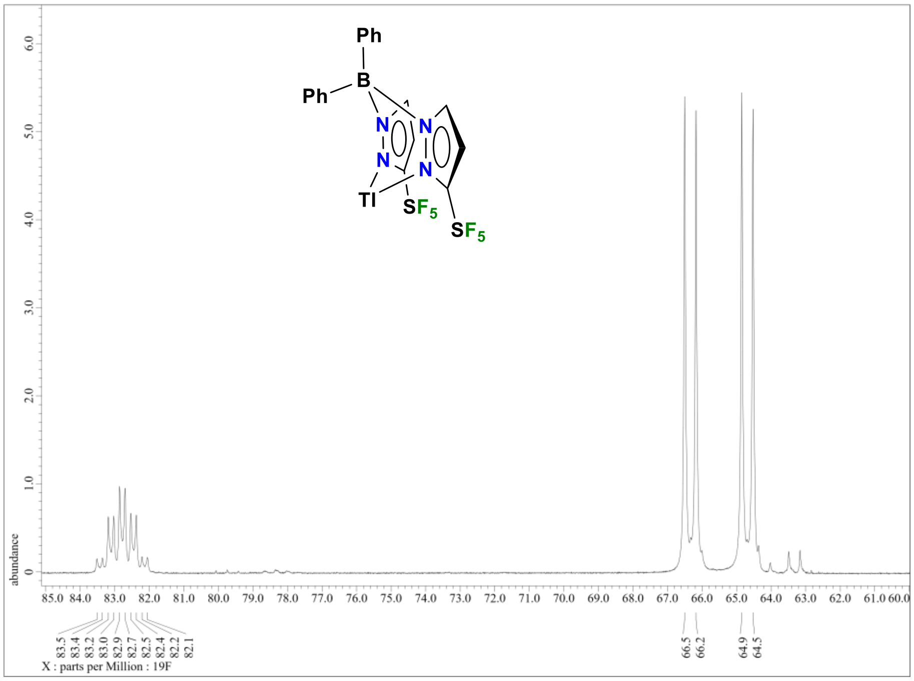

Figure S14: ^{19}F NMR Spectrum of **$[Ph_2B(3-(SF_5)Pz)_2]Tl$** in $CDCl_3$.

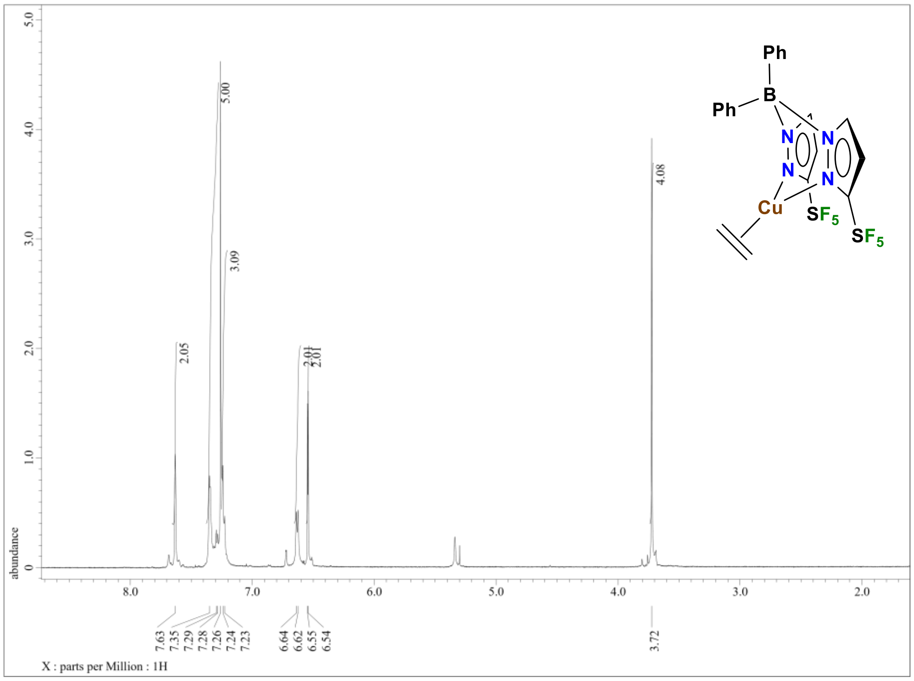

Figure S15: ^{1}H NMR Spectrum of **[Ph$_2$B(3-(SF$_5$)Pz)$_2$]Cu(C$_2$H$_4$)** in $CDCl_3$

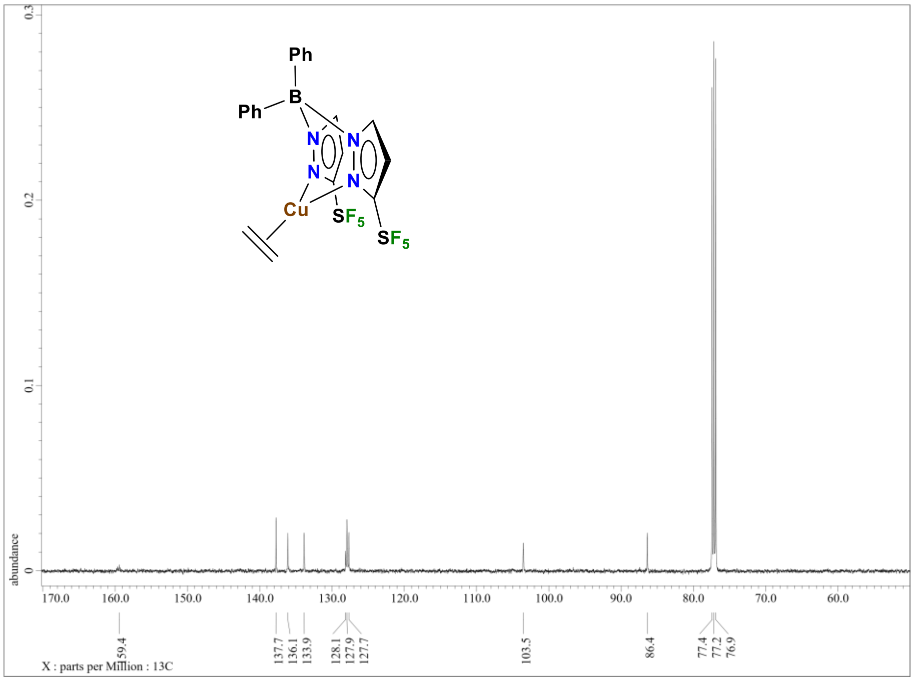

Figure S16: ^{13}C NMR Spectrum of **[Ph$_2$B(3-(SF$_5$)Pz)$_2$]Cu(C$_2$H$_4$)** in $CDCl_3$.

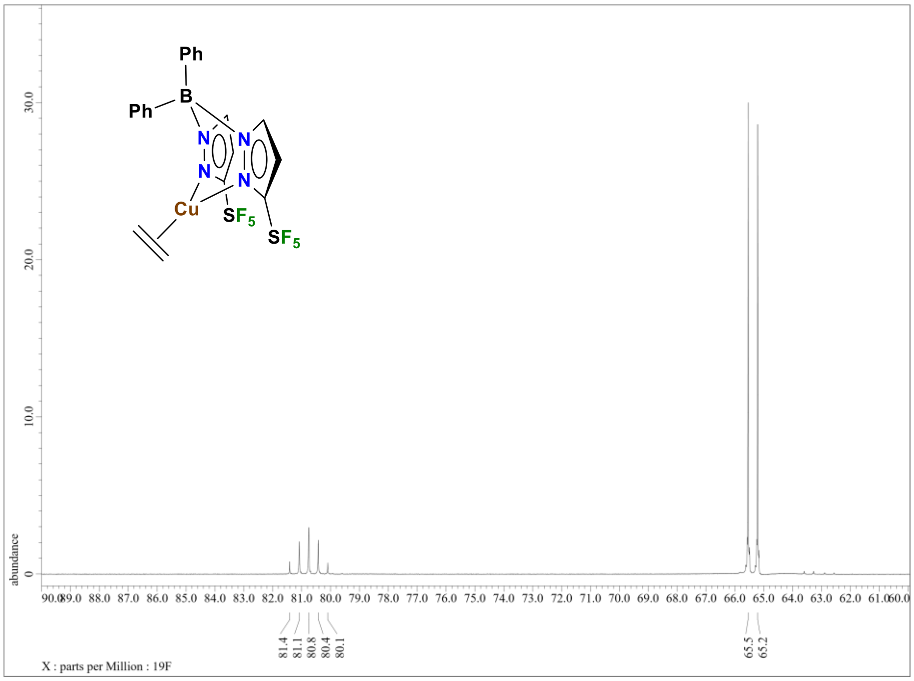

Figure S17: ^{19}F NMR Spectrum of **[Ph$_2$B(3-(SF$_5$)Pz)$_2$]Cu(C$_2$H$_4$)** in CDCl$_3$.

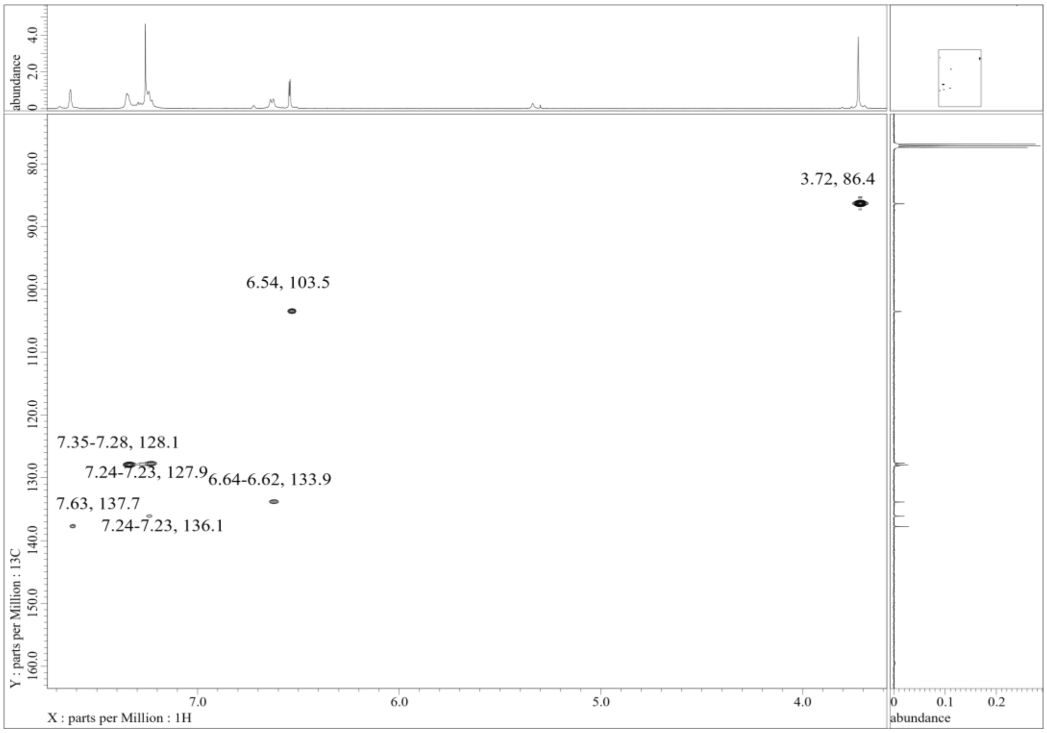

Figure S18: HSQC NMR Spectrum of **[Ph$_2$B(3-(SF$_5$)Pz)$_2$]Cu(C$_2$H$_4$)** in CDCl$_3$.

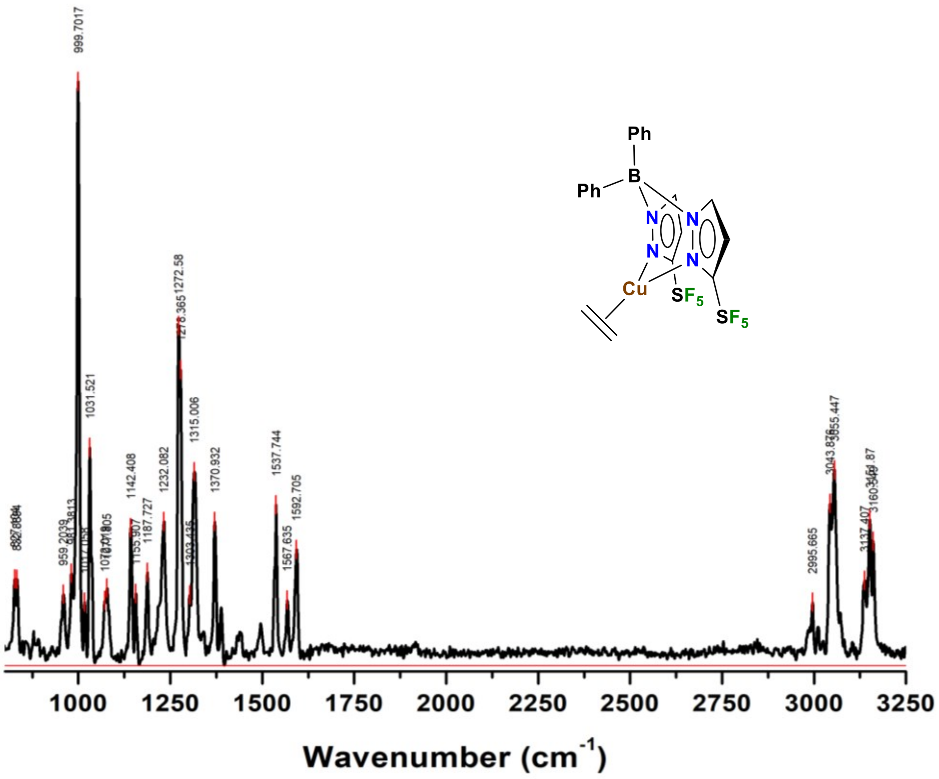

Figure S19: Raman Spectrum of **[Ph$_2$B(3-SF$_5$)Pz)$_2$]Cu(C$_2$H$_4$)**.

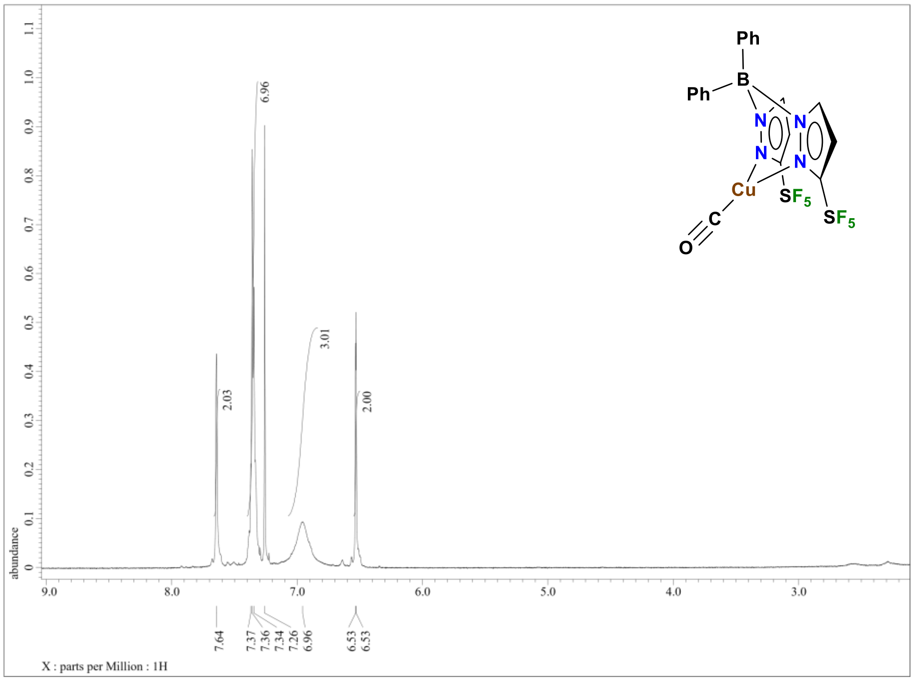

Figure S20: ^{1}H NMR Spectrum of **[Ph$_2$B(3-(SF$_5$)Pz)$_2$]Cu(CO)** in CDCl$_3$

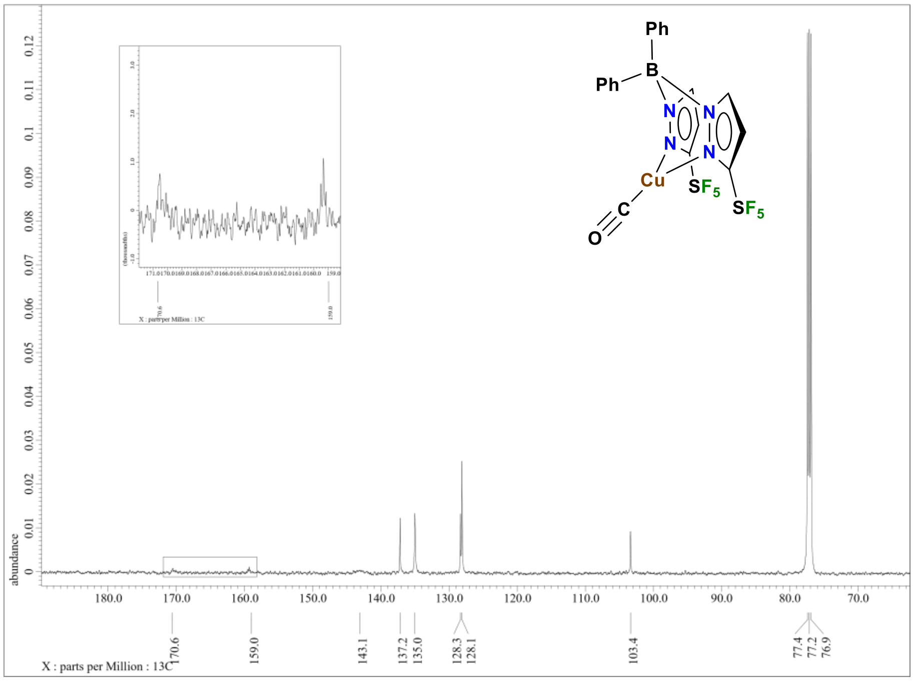

Figure S21: ^{13}C NMR Spectrum of **[Ph$_2$B(3-(SF$_5$)Pz)$_2$]Cu(CO)** in $CDCl_3$

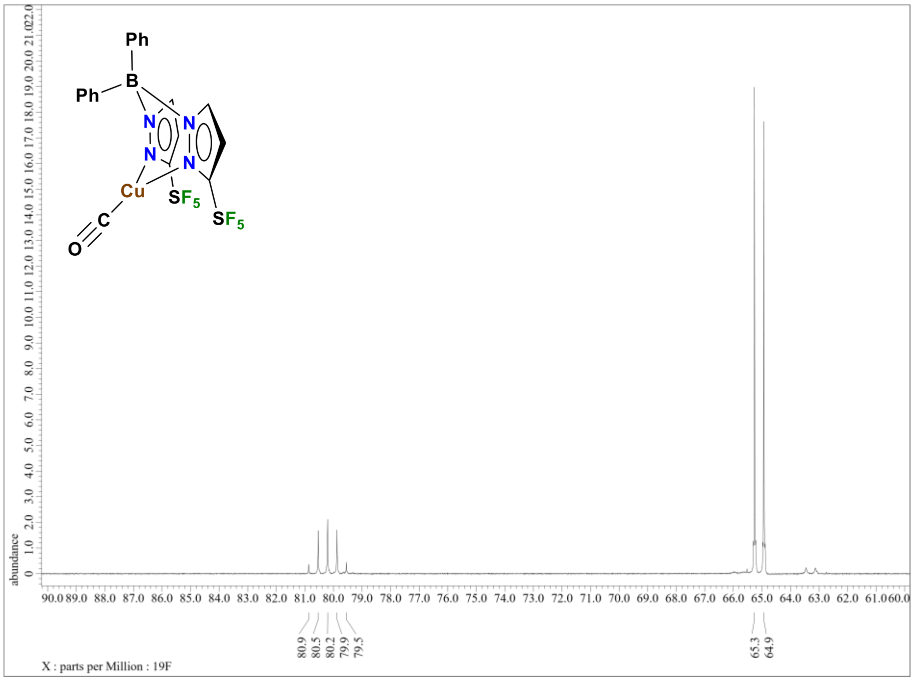

Figure S22: ^{19}F NMR Spectrum of **[Ph$_2$B(3-(SF$_5$)Pz)$_2$]Cu(CO)** in $CDCl_3$

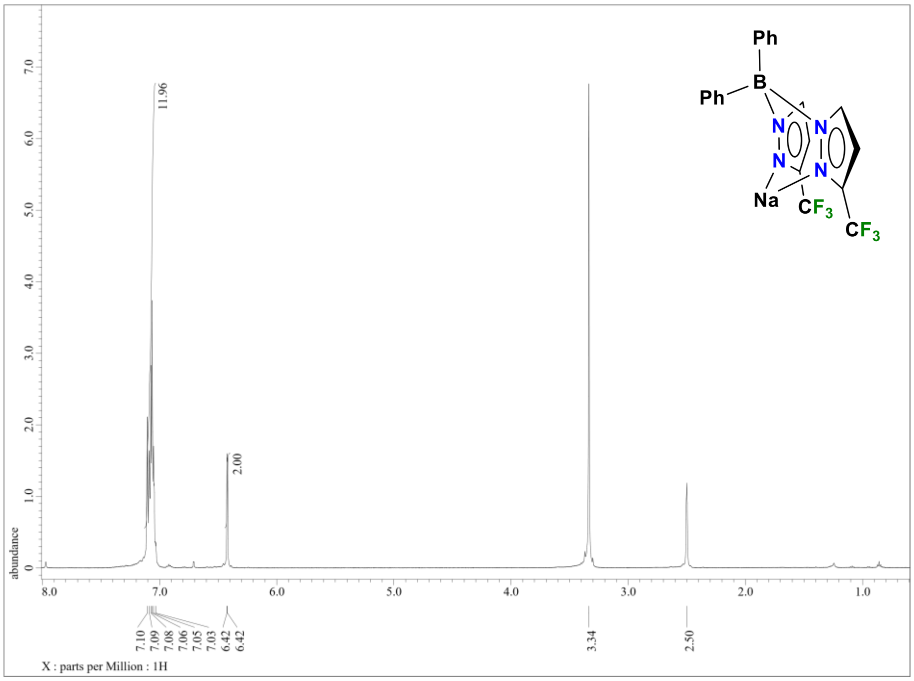

Figure S23: ^{1}H NMR Spectrum of **[Ph$_2$B(3-CF$_3$)Pz)$_2$]Na** in (CD$_3$)$_2$SO.

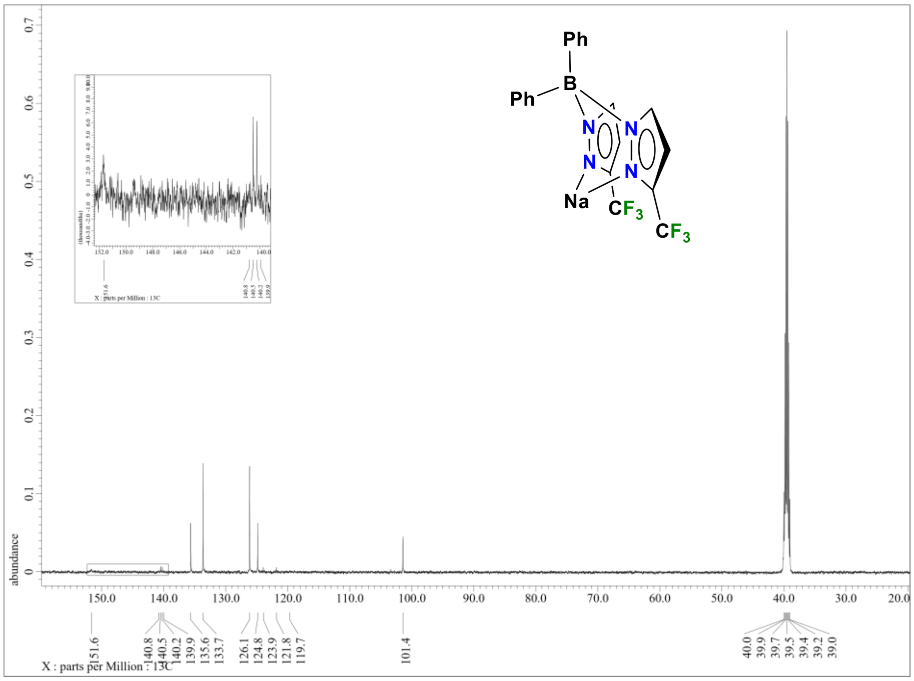

Figure S24: ^{13}C NMR Spectrum of **[Ph$_2$B(3-CF$_3$)Pz)$_2$]Na** in (CD$_3$)$_2$SO.

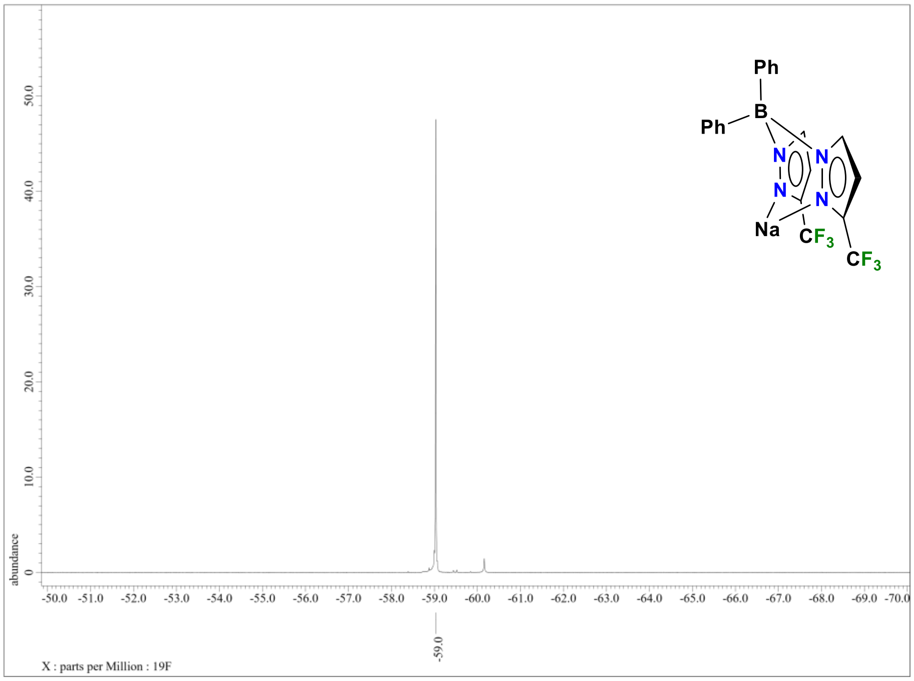

Figure S25: ^{19}F NMR Spectrum of **[$Ph_2B(3\text{-}CF_3)Pz)_2$]Na** in $(CD_3)_2SO$.

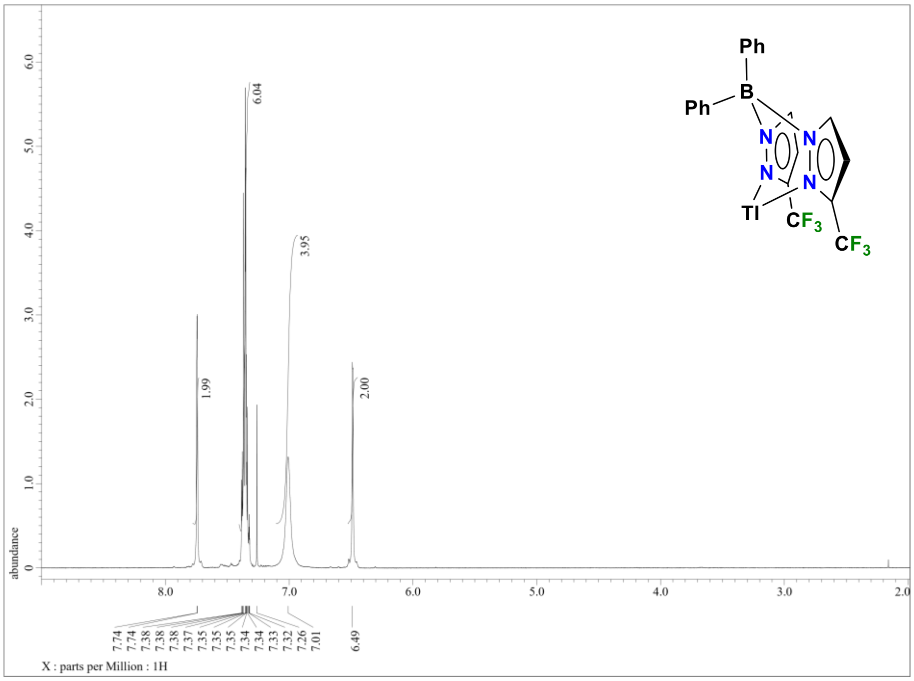

Figure S26: ^{1}H NMR Spectrum of **[$Ph_2B(3\text{-}CF_3)Pz)_2$]Tl** in $CDCl_3$

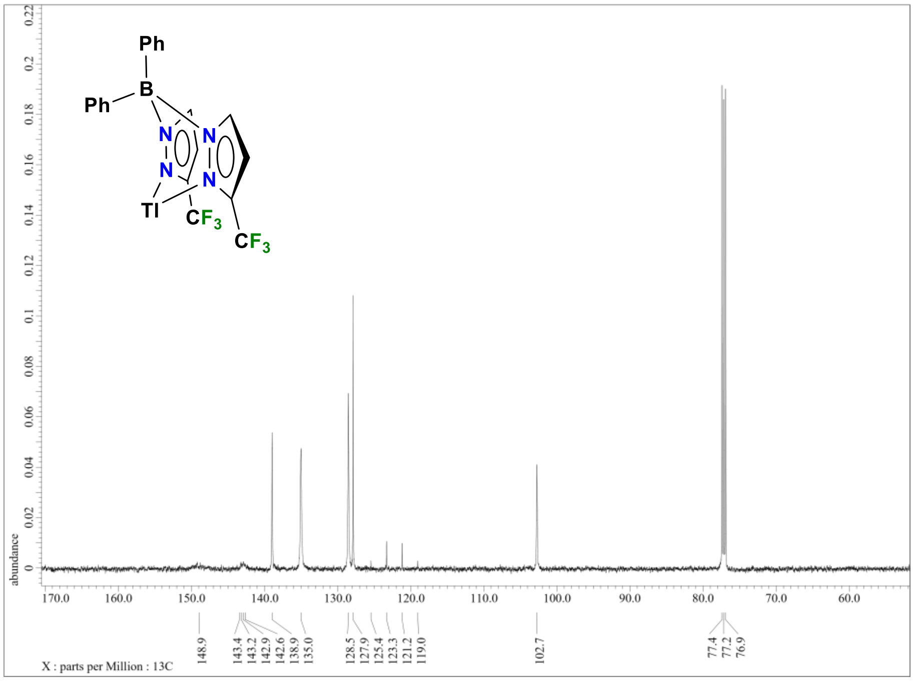

Figure S27: ^{13}C NMR Spectrum of **[Ph$_2$B(3-CF$_3$)Pz)$_2$]Tl** in CDCl$_3$.

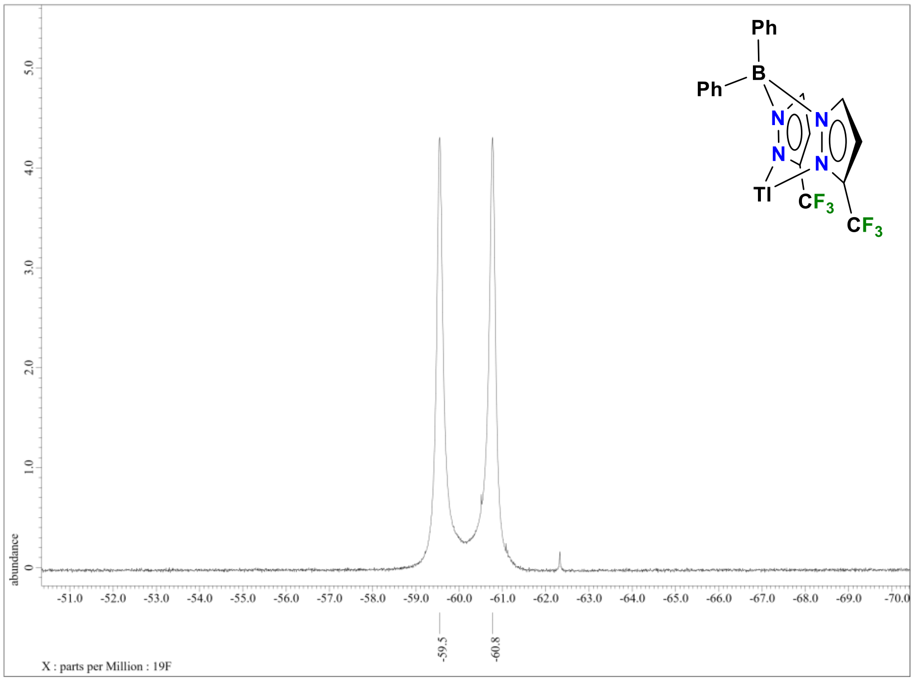

Figure S28: ^{19}F NMR Spectrum of **[Ph$_2$B(3-CF$_3$)Pz)$_2$]Tl** in CDCl$_3$.

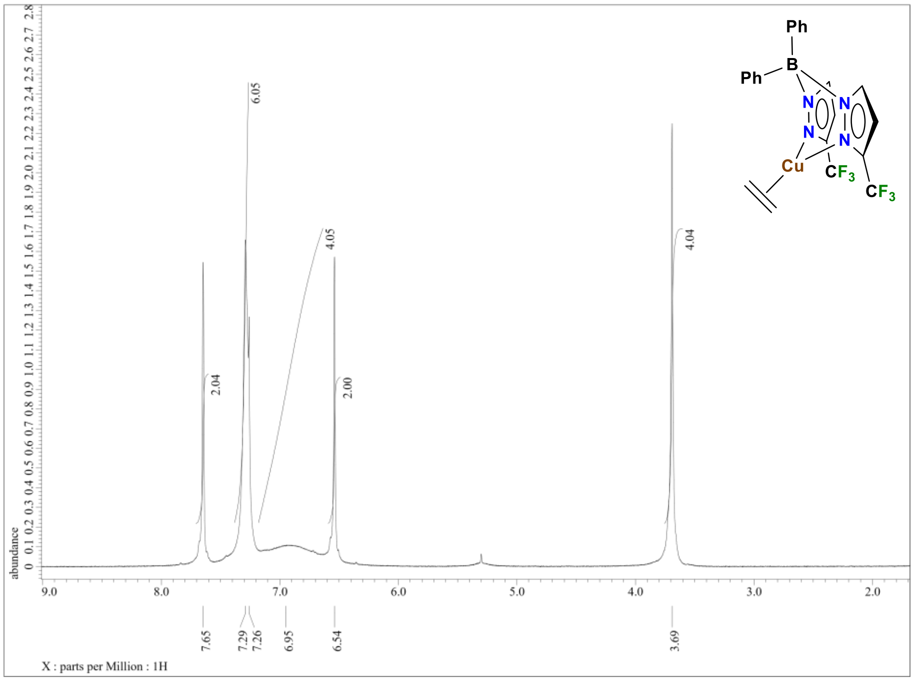

Figure S29: ^{1}H NMR Spectrum of **[Ph$_2$B(3-CF$_3$)Pz)$_2$]Cu(C$_2$H$_4$)** in CDCl$_3$

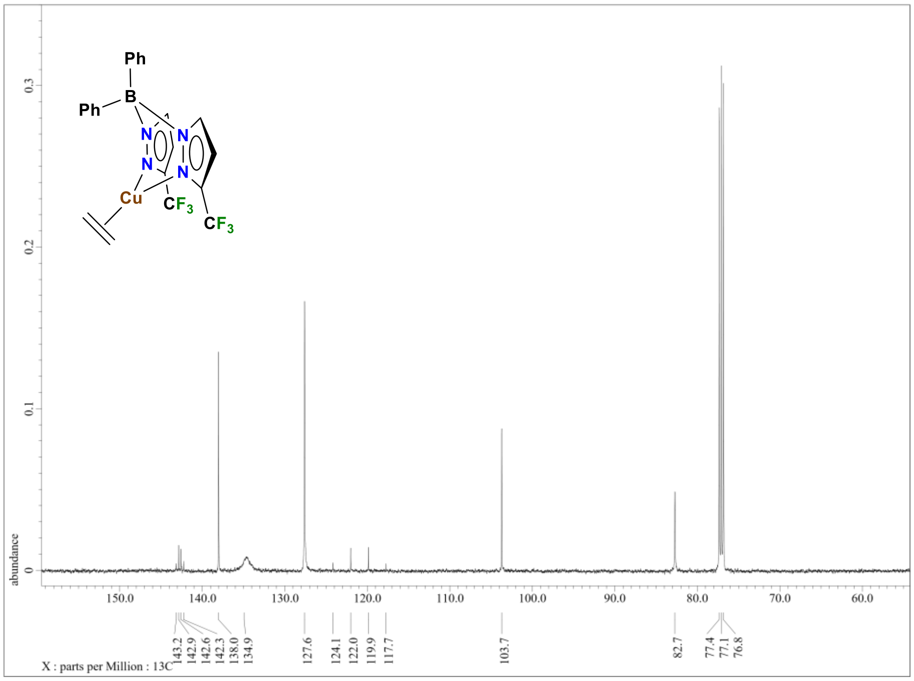

Figure S30: ^{13}C NMR Spectrum of **[Ph$_2$B(3-CF$_3$)Pz)$_2$]Cu(C$_2$H$_4$)** in CDCl$_3$.

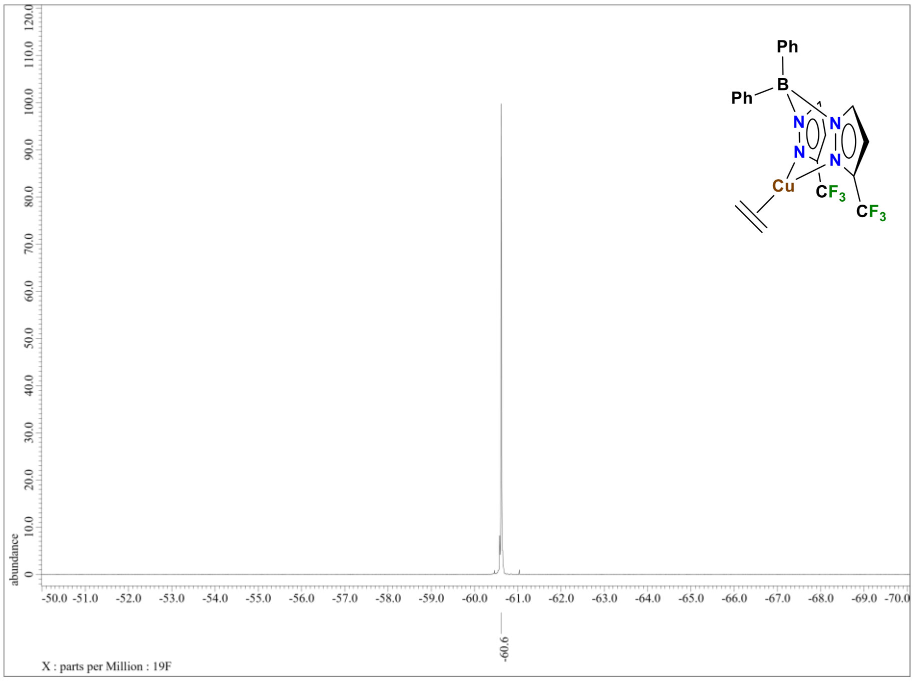

Figure S31: ^{19}F NMR Spectrum of **[Ph$_2$B(3-CF$_3$)Pz)$_2$]Cu(C$_2$H$_4$)** in CDCl$_3$.

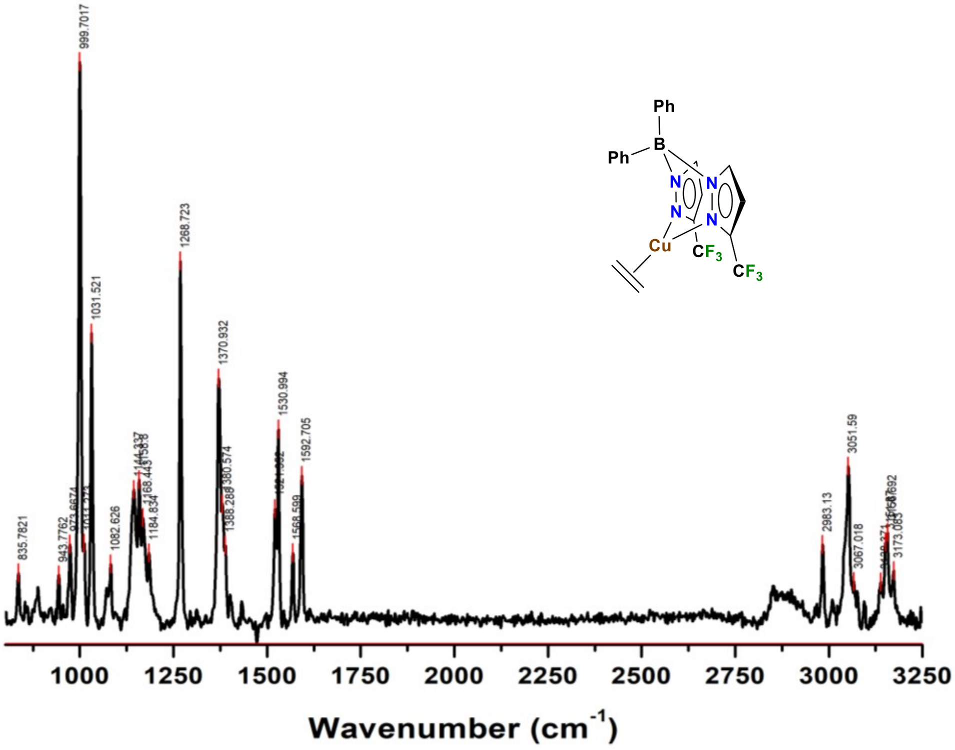

Figure S32: Raman Spectrum of **[Ph$_2$B(3-CF$_3$)Pz)$_2$]Cu(C$_2$H$_4$)**.

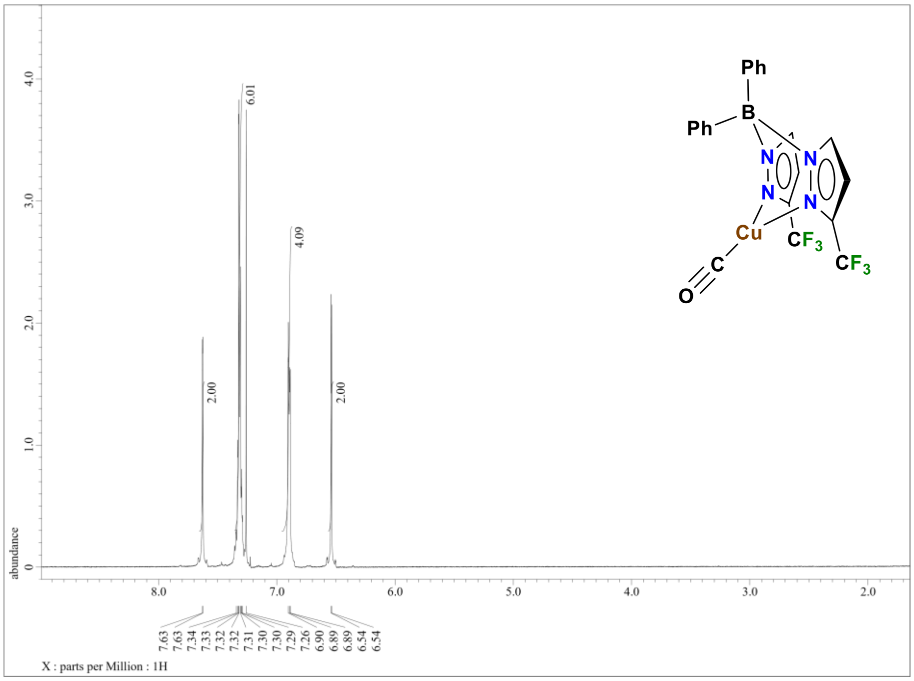

Figure S33: ^{1}H NMR Spectrum of **[Ph$_2$B(3-CF$_3$)Pz)$_2$]Cu(CO)** in $CDCl_3$

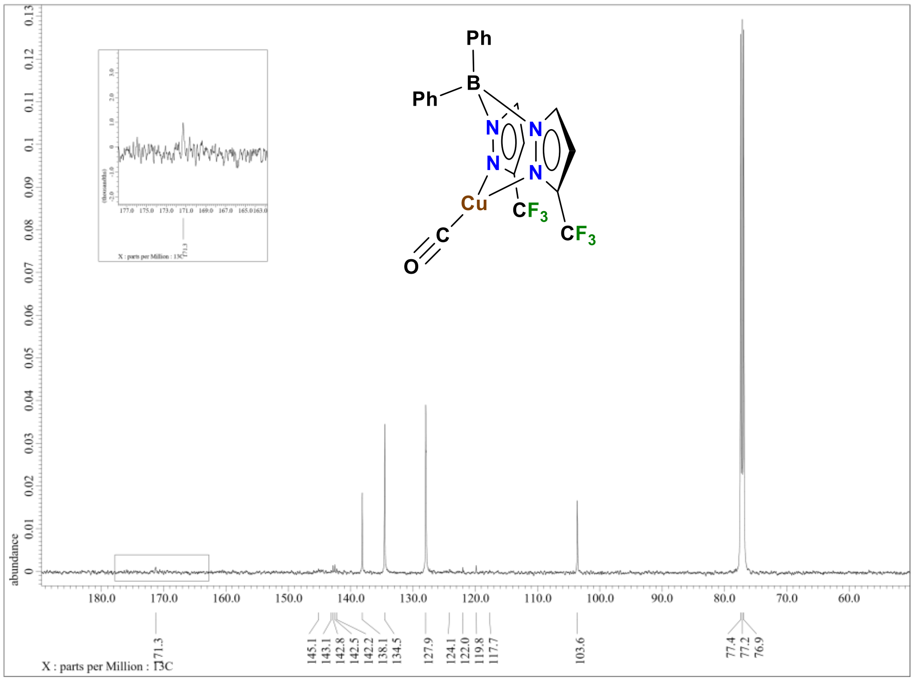

Figure S34: ^{13}C NMR Spectrum of **[Ph$_2$B(3-CF$_3$)Pz)$_2$]Cu(CO)** in $CDCl_3$.

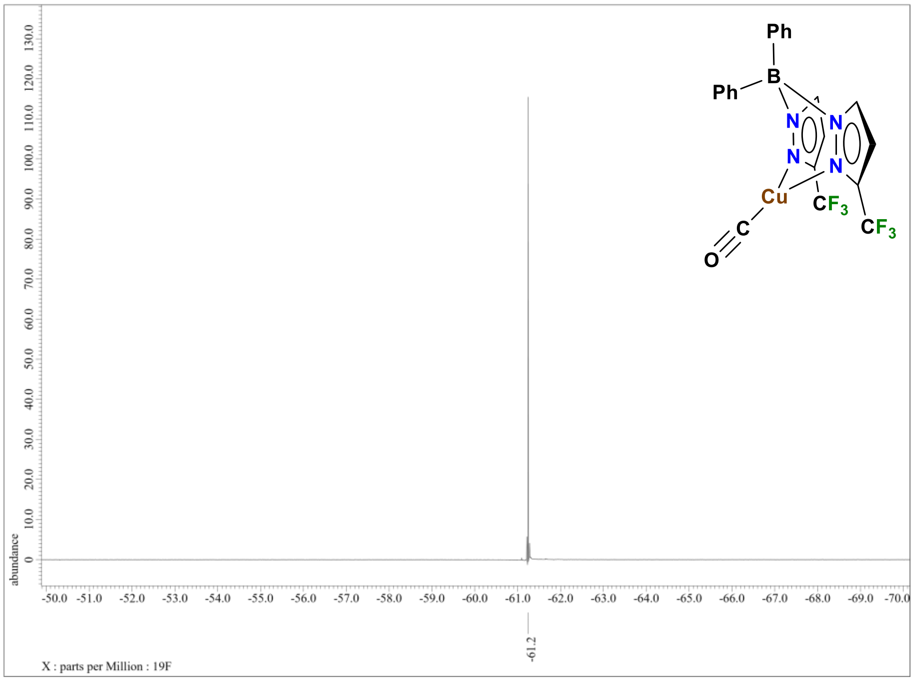

Figure S35: ^{19}F NMR Spectrum of **[$Ph_2B(3\text{-}CF_3)Pz)_2$]Cu(CO)** in $CDCl_3$.

X-ray Data Collection and Structure Determinations

A suitable crystal covered with a layer of hydrocarbon/Paratone-N oil was selected and mounted on a Cryo-loop, and immediately placed in the low-temperature nitrogen stream. The X-ray intensity data of [$Ph_2B(3-(SF_5)Pz)_2$]$Cu(C_2H_4)$ and [$Ph_2B(3-(CF_3)Pz)_2$]$Cu(C_2H_4)$ were measured at 100 K, on a Bruker system with a SMART APEX II CCD area detector system, while data of [$Ph_2B(3-(SF_5)Pz)_2$]Cu(CO) and [$Ph_2B(3-(CF_3)Pz)_2$]Cu(CO) were measured at 100(2) K on a Bruker D8 Quest with a PHOTON II 7 CPAD detector. Both instruments were equipped with an Oxford Cryosystems 700 series cooler, a graphite monochromator, and a Mo Kα fine-focus sealed tube (λ = 0.71073 Å). Intensity data were processed using the Bruker Apex program suite. Absorption corrections were applied by using SADABS.[6] Initial atomic positions were located by SHELXT,[7] and the structures of the compounds were refined by the least-squares method using SHELXL[8] within Olex2 GUI.[9] All the non-hydrogen atoms were refined anisotropically. The hydrogen atoms of ethylene moieties of [$Ph_2B(3-(SF_5)Pz)_2$]$Cu(C_2H_4)$ and [$Ph_2B(3-(CF_3)Pz)_2$]$Cu(C_2H_4)$ were located in difference Fourier maps, included and refined freely with isotropic displacement parameters. The remaining hydrogen atoms were included in their calculated positions and refined as riding on the atoms to which they are joined. The [$Ph_2B(3-(SF_5)Pz)_2$]$Cu(C_2H_4)$ and [$Ph_2B(3-(SF_5)Pz)_2$]Cu(CO) complexes crystallize in $P\bar{1}$ space group with two chemically identical molecules in the asymmetric unit. X-ray structural figures were generated using Olex2.[9] The **CCDC** 2104704-2104707 files contain the supplementary crystallographic data. These data can be obtained free of charge via http://www.ccdc.cam.ac.uk/conts/retrieving.html or from the Cambridge Crystallographic Data Centre (CCDC), 12 Union Road, Cambridge, CB2 1EZ, UK).

Table S3. Selected bond distances (Å) and angles (º) of copper(I) complexes. Data for the second molecule in the asymmetric unit in *italics*.

Parameter	$[Ph_2B(3-(SF_5)Pz)_2]Cu(C_2H_4)$	$[Ph_2B(3-CF_3)Pz)_2]Cu(C_2H_4)$	$[Ph_2B(3-SF_5)Pz_2]Cu(CO)$	$[Ph_2B(3-CF_3)Pz)_2]Cu(CO)$
C=C	1.369(2)<br>*1.353(2)*	1.3750(17)	-	-
C≡O	-	-	1.120(2)<br>*1.121(2)*	1.119(2)
Cu-C	2.0199(13)<br>2.0225(13)<br>*2.0307(14)*<br>*2.0230(15)*	2.0123(11)<br>2.0184(11)	1.803(2)<br>*1.807(2)*	1.8028(16)
Cu••C(Phenyl)	2.875<br>*2.723*	2.957	2.643<br>*2.510*	2.778
Cu-N	1.9937(10)<br>1.9870(10)<br>*1.9980(10)*<br>*2.0075(11)*	1.9745(8)<br>1.9795(8)	2.0054(15)<br>1.9910(15)<br>*2.0154(15)*<br>*2.0094(16)*	1.9871(11)<br>1.9838(10)
N-Cu-N	93.05(4)<br>*92.30(4)*	95.12(3)	92.50(6)<br>*91.24(6)*	93.92(4)
C-Cu-C	39.59(6)<br>*39.00(6)*	39.89(5)	-	-
Cu-C-O	-	-	179.3(2)<br>*172.2(2)*	176.4(2)
N-Cu-C	-	-	134.34(8)<br>131.12(8)<br>*130.38(8)*<br>*138.06(8)*	137.21(6)<br>128.47(6)
Σ angles at Cu	359.42<br>*360.00*	359.98	357.96<br>*359.68*	359.61

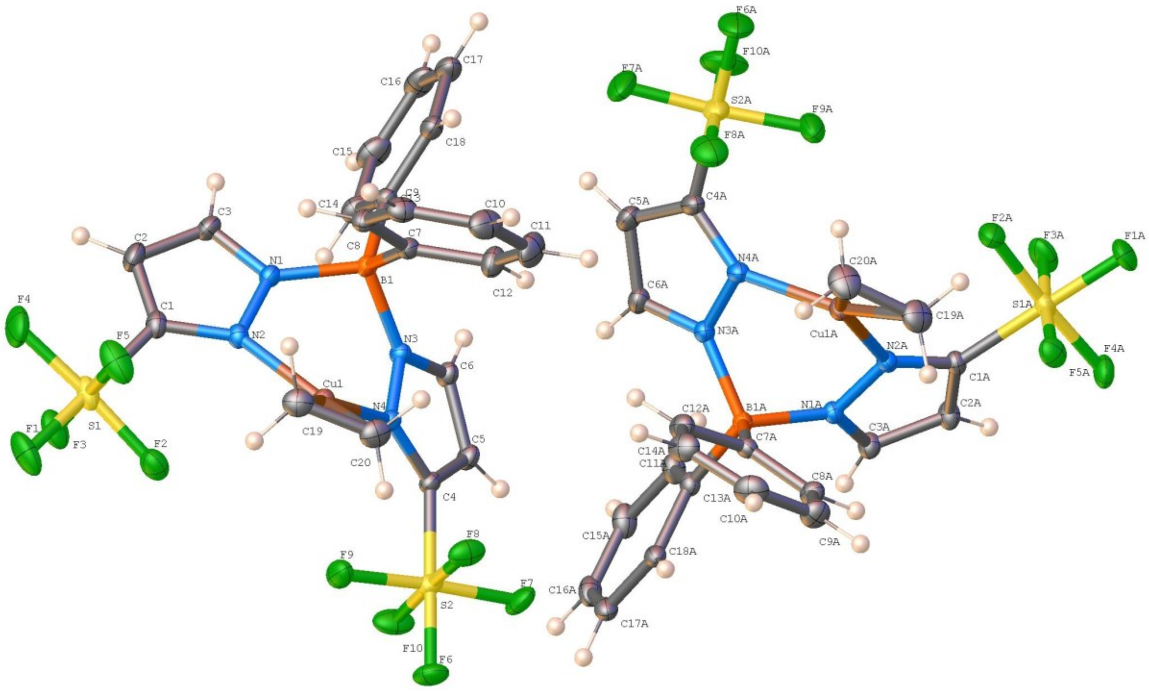

Figure S36: Molecular structure and atom labelling scheme of **$[Ph_2B(3\text{-}(SF_5)Pz)_2]Cu(C_2H_4)$.**

Table S4: Crystal data and structure refinement for **$[Ph_2B(3\text{-}(SF_5)Pz)_2]Cu(C_2H_4)$**.

Identification code	rad992_0m_a
Empirical formula	$C_{20}H_{18}BCuF_{10}N_4S_2$
Formula weight	642.85
Temperature/K	100.0
Crystal system	triclinic
Space group	P-1
a/Å	11.6035(8)
b/Å	14.7339(11)
c/Å	15.7485(11)
α/°	80.1640(10)

β/°	76.2550(10)
γ/°	68.3860(10)
Volume/Å^3	2421.1(3)
Z	4
ρ_{calc}g/cm^3	1.764
μ/mm^{-1}	1.171
F(000)	1288.0
Crystal size/mm^3	0.36 × 0.33 × 0.27
Radiation	MoKα (λ = 0.71073)
2Θ range for data collection/°	3.846 to 66.378
Index ranges	-17 ≤ h ≤ 17, -22 ≤ k ≤ 22, -24 ≤ l ≤ 23
Reflections collected	32946
Independent reflections	17011 [R_{int} = 0.0154, R_{sigma} = 0.0251]
Data/restraints/parameters	17011/0/717
Goodness-of-fit on F^2	1.015
Final R indexes [I>=2σ (I)]	R_1 = 0.0294, wR_2 = 0.0778
Final R indexes [all data]	R_1 = 0.0346, wR_2 = 0.0805
Largest diff. peak/hole / e Å^{-3}	0.89/-0.58

Table S5: Bond Lengths for **[$Ph_2B(3-(SF_5)Pz)_2$]$Cu(C_2H_4)$.**

Atom	Atom	Length/Å	Atom	Atom	Length/Å
Cu1	N2	1.9937(10)	Cu1A	N2A	1.9980(10)
Cu1	N4	1.9870(10)	Cu1A	N4A	2.0075(11)
Cu1	C19	2.0199(13)	Cu1A	C19A	2.0307(14)
Cu1	C20	2.0225(13)	Cu1A	C20A	2.0230(15)
S1	F1	1.5828(10)	S1A	F1A	1.5775(9)
S1	F2	1.5792(10)	S1A	F2A	1.5866(9)
S1	F3	1.5839(9)	S1A	F3A	1.5834(9)
S1	F4	1.5744(11)	S1A	F4A	1.5843(9)
S1	F5	1.5867(9)	S1A	F5A	1.5855(9)
S1	C1	1.7864(13)	S1A	C1A	1.7821(12)

Table S5: Bond Lengths for **[$Ph_2B(3\text{-}(SF_5)Pz)_2$]Cu($C_2H_4$).**

Atom	Atom	Length/Å	Atom	Atom	Length/Å
S2	F6	1.5825(9)	S2A	F6A	1.5804(10)
S2	F7	1.5765(9)	S2A	F7A	1.5741(10)
S2	F8	1.5830(9)	S2A	F8A	1.5804(12)
S2	F9	1.5824(9)	S2A	F9A	1.5836(10)
S2	F10	1.5796(9)	S2A	F10A	1.5824(13)
S2	C4	1.7846(12)	S2A	C4A	1.7828(14)
N1	N2	1.3612(14)	N1A	N2A	1.3612(14)
N1	C3	1.3491(15)	N1A	C3A	1.3475(15)
N1	B1	1.5881(16)	N1A	B1A	1.5853(16)
N2	C1	1.3377(15)	N2A	C1A	1.3393(15)
N3	N4	1.3608(14)	N3A	N4A	1.3611(14)
N3	C6	1.3497(15)	N3A	C6A	1.3476(16)
N3	B1	1.5916(16)	N3A	B1A	1.5935(16)
N4	C4	1.3405(15)	N4A	C4A	1.3371(15)
C1	C2	1.3907(19)	C1A	C2A	1.3914(17)
C2	C3	1.3812(19)	C2A	C3A	1.3838(17)
C4	C5	1.3906(17)	C4A	C5A	1.392(2)
C5	C6	1.3825(17)	C5A	C6A	1.3828(19)
C7	C8	1.3982(17)	C7A	C8A	1.4037(17)
C7	C12	1.4040(17)	C7A	C12A	1.4052(16)
C7	B1	1.6152(18)	C7A	B1A	1.6162(18)
C8	C9	1.3963(18)	C8A	C9A	1.3959(18)
C9	C10	1.388(2)	C9A	C10A	1.391(2)
C10	C11	1.395(2)	C10A	C11A	1.391(2)
C11	C12	1.387(2)	C11A	C12A	1.3926(19)
C13	C14	1.4054(19)	C13A	C14A	1.4094(18)
C13	C18	1.4032(18)	C13A	C18A	1.4026(17)
C13	B1	1.6134(18)	C13A	B1A	1.6116(17)
C14	C15	1.397(2)	C14A	C15A	1.3889(19)

Table S5: Bond Lengths for **[$Ph_2B(3\text{-}(SF_5)Pz)_2$]Cu($C_2H_4$).**

Atom	Atom	Length/Å	Atom	Atom	Length/Å
C15	C16	1.385(2)	C15A	C16A	1.391(2)
C16	C17	1.389(2)	C16A	C17A	1.384(2)
C17	C18	1.3983(19)	C17A	C18A	1.3991(17)
C19	C20	1.369(2)	C19A	C20A	1.353(2)

Table S6: Bond Angles for **[$Ph_2B(3\text{-}(SF_5)Pz)_2$]Cu($C_2H_4$)**.

Atom	Atom	Atom	Angle/˚	Atom	Atom	Atom	Angle/˚
N2	Cu1	C19	110.98(5)	N2A	Cu1A	N4A	92.30(4)
N2	Cu1	C20	149.44(5)	N2A	Cu1A	C19A	112.86(5)
N4	Cu1	N2	93.05(4)	N2A	Cu1A	C20A	151.59(6)
N4	Cu1	C19	155.78(5)	N4A	Cu1A	C19A	154.79(6)
N4	Cu1	C20	116.21(5)	N4A	Cu1A	C20A	115.99(6)
C19	Cu1	C20	39.59(6)	C20A	Cu1A	C19A	39.00(6)
F1	S1	F3	87.63(5)	F1A	S1A	F2A	87.97(5)
F1	S1	F5	88.02(5)	F1A	S1A	F3A	88.31(5)
F1	S1	C1	179.05(6)	F1A	S1A	F4A	88.25(5)
F2	S1	F1	87.80(6)	F1A	S1A	F5A	87.92(5)
F2	S1	F3	89.69(5)	F1A	S1A	C1A	179.46(6)
F2	S1	F5	89.94(6)	F2A	S1A	C1A	92.56(5)
F2	S1	C1	92.91(5)	F3A	S1A	F2A	89.93(5)
F3	S1	F5	175.65(5)	F3A	S1A	F4A	89.77(5)
F3	S1	C1	91.73(5)	F3A	S1A	F5A	176.23(5)
F4	S1	F1	88.05(6)	F3A	S1A	C1A	91.77(5)
F4	S1	F2	175.85(6)	F4A	S1A	F2A	176.22(5)
F4	S1	F3	89.97(6)	F4A	S1A	F5A	89.99(5)
F4	S1	F5	90.08(6)	F4A	S1A	C1A	91.21(5)
F4	S1	C1	91.23(6)	F5A	S1A	F2A	90.06(5)
F5	S1	C1	92.62(6)	F5A	S1A	C1A	92.00(5)

Table S6: Bond Angles for **[$Ph_2B(3\text{-}(SF_5)Pz)_2$]$Cu(C_2H_4)$**.

Atom	Atom	Atom	Angle/˚	Atom	Atom	Atom	Angle/˚
F6	S2	F8	87.83(5)	F6A	S2A	F9A	87.89(6)
F6	S2	C4	179.44(5)	F6A	S2A	F10A	88.35(7)
F7	S2	F6	87.95(5)	F6A	S2A	C4A	179.57(6)
F7	S2	F8	89.93(5)	F7A	S2A	F6A	88.07(6)
F7	S2	F9	175.97(5)	F7A	S2A	F8A	89.80(7)
F7	S2	F10	90.01(6)	F7A	S2A	F9A	175.96(6)
F7	S2	C4	91.53(5)	F7A	S2A	F10A	90.16(7)
F8	S2	C4	92.36(5)	F7A	S2A	C4A	91.56(6)
F9	S2	F6	88.03(5)	F8A	S2A	F6A	88.00(7)
F9	S2	F8	89.79(5)	F8A	S2A	F9A	89.80(6)
F9	S2	C4	92.50(5)	F8A	S2A	F10A	176.35(6)
F10	S2	F6	88.11(5)	F8A	S2A	C4A	92.23(6)
F10	S2	F8	175.94(5)	F9A	S2A	C4A	92.48(6)
F10	S2	F9	89.98(6)	F10A	S2A	F9A	89.98(7)
F10	S2	C4	91.69(5)	F10A	S2A	C4A	91.42(7)
N2	N1	B1	120.67(9)	N2A	N1A	B1A	119.01(9)
C3	N1	N2	109.87(10)	C3A	N1A	N2A	109.84(10)
C3	N1	B1	129.27(10)	C3A	N1A	B1A	131.12(10)
N1	N2	Cu1	113.98(7)	N1A	N2A	Cu1A	113.70(7)
C1	N2	Cu1	139.69(9)	C1A	N2A	Cu1A	140.69(8)
C1	N2	N1	105.59(10)	C1A	N2A	N1A	105.60(10)
N4	N3	B1	119.52(9)	N4A	N3A	B1A	118.04(9)
C6	N3	N4	109.75(10)	C6A	N3A	N4A	109.81(10)
C6	N3	B1	130.39(10)	C6A	N3A	B1A	132.06(10)
N3	N4	Cu1	115.04(7)	N3A	N4A	Cu1A	114.13(7)
C4	N4	Cu1	139.33(8)	C4A	N4A	Cu1A	140.06(9)
C4	N4	N3	105.63(10)	C4A	N4A	N3A	105.65(10)
N2	C1	S1	121.54(10)	N2A	C1A	S1A	121.83(9)
N2	C1	C2	111.90(11)	N2A	C1A	C2A	111.94(10)

Table S6: Bond Angles for **[$Ph_2B(3\text{-}(SF_5)Pz)_2$]$Cu(C_2H_4)$**.

Atom	Atom	Atom	Angle/°	Atom	Atom	Atom	Angle/°
C2	C1	S1	126.49(10)	C2A	C1A	S1A	126.22(9)
C3	C2	C1	103.67(11)	C3A	C2A	C1A	103.47(10)
N1	C3	C2	108.97(12)	N1A	C3A	C2A	109.13(11)
N4	C4	S2	121.10(9)	N4A	C4A	S2A	121.37(10)
N4	C4	C5	111.92(10)	N4A	C4A	C5A	111.95(11)
C5	C4	S2	126.98(9)	C5A	C4A	S2A	126.68(10)
C6	C5	C4	103.54(10)	C6A	C5A	C4A	103.44(11)
N3	C6	C5	109.15(11)	N3A	C6A	C5A	109.15(12)
C8	C7	C12	116.70(12)	C8A	C7A	C12A	116.84(11)
C8	C7	B1	123.75(10)	C8A	C7A	B1A	122.57(10)
C12	C7	B1	119.55(11)	C12A	C7A	B1A	120.55(10)
C9	C8	C7	122.03(12)	C9A	C8A	C7A	121.60(11)
C10	C9	C8	119.96(13)	C10A	C9A	C8A	120.16(12)
C9	C10	C11	119.11(13)	C9A	C10A	C11A	119.41(13)
C12	C11	C10	120.34(13)	C10A	C11A	C12A	120.00(12)
C11	C12	C7	121.80(13)	C11A	C12A	C7A	121.89(12)
C14	C13	B1	121.85(11)	C14A	C13A	B1A	123.10(11)
C18	C13	C14	116.82(12)	C18A	C13A	C14A	116.38(11)
C18	C13	B1	121.33(12)	C18A	C13A	B1A	120.49(11)
C15	C14	C13	121.97(14)	C15A	C14A	C13A	121.97(12)
C16	C15	C14	119.84(15)	C14A	C15A	C16A	120.22(13)
C15	C16	C17	119.67(13)	C17A	C16A	C15A	119.36(12)
C16	C17	C18	120.26(14)	C16A	C17A	C18A	120.15(12)
C17	C18	C13	121.43(14)	C17A	C18A	C13A	121.90(12)
C20	C19	Cu1	70.31(8)	C20A	C19A	Cu1A	70.19(9)
C19	C20	Cu1	70.10(8)	C19A	C20A	Cu1A	70.81(9)
N1	B1	N3	106.20(9)	N1A	B1A	N3A	104.80(9)
N1	B1	C7	109.61(10)	N1A	B1A	C7A	109.71(9)
N1	B1	C13	109.15(9)	N1A	B1A	C13A	110.11(9)

Table S6: Bond Angles for **[$Ph_2B(3\text{-}(SF_5)Pz)_2$]$Cu(C_2H_4)$**.

**Atom**	**Atom**	**Atom**	**Angle/˚**	**Atom**	**Atom**	**Atom**	**Angle/˚**
N3	B1	C7	106.25(9)	N3A	B1A	C7A	106.77(9)
N3	B1	C13	108.92(10)	N3A	B1A	C13A	110.05(9)
C13	B1	C7	116.22(10)	C13A	B1A	C7A	114.87(10)

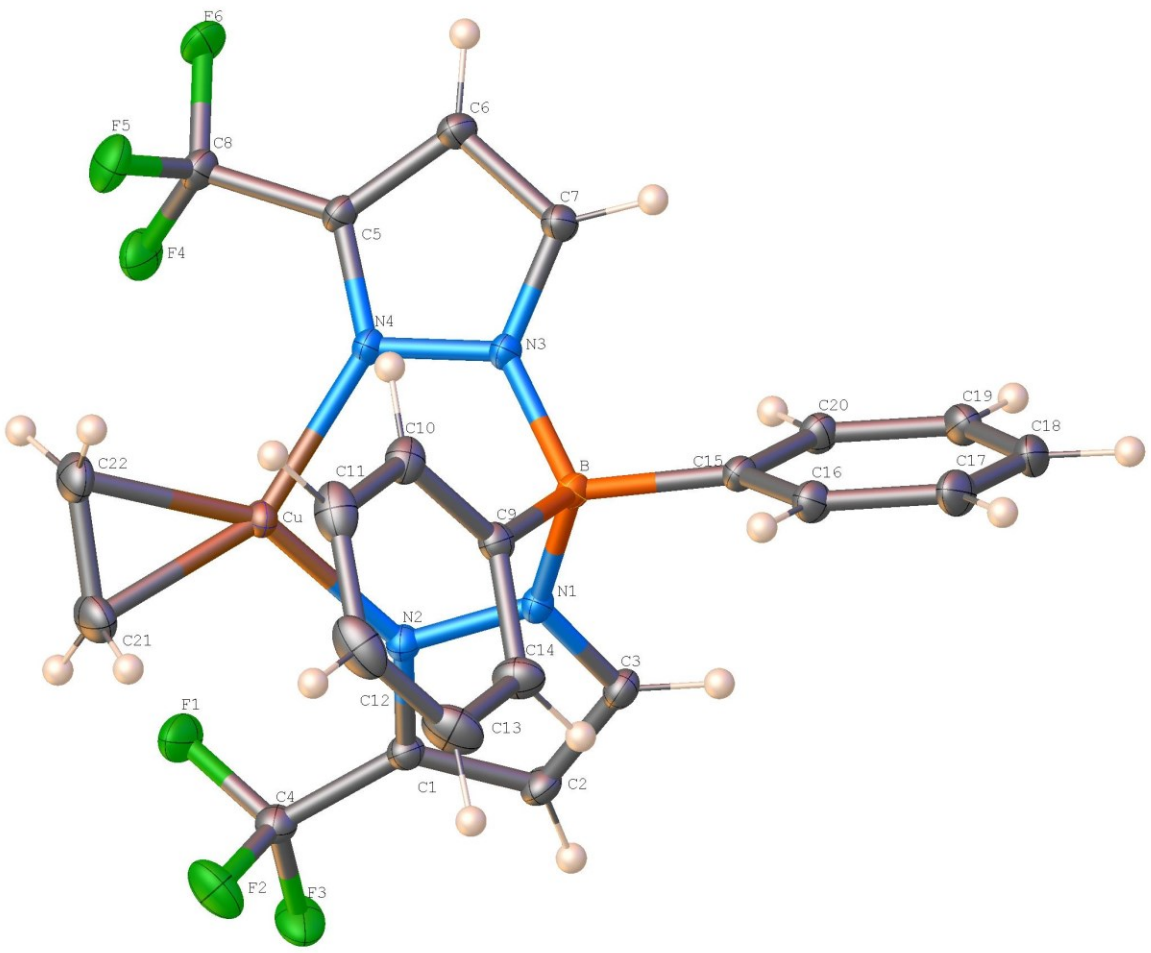

Figure S37: Molecular structure and atom labelling scheme of **[$Ph_2B(3\text{-}CF_3)Pz)_2$]$Cu(C_2H_4)$.**

Table S7: Crystal data and structure refinement for **[$Ph_2B(3\text{-}CF_3)Pz)_2$]$Cu(C_2H_4)$**.

Identification code	rad994_0m_a
Empirical formula	$C_{22}H_{18}BCuF_6N_4$
Formula weight	526.75
Temperature/K	100
Crystal system	triclinic

Space group	P-1
a/Å	9.9858(7)
b/Å	10.2229(7)
c/Å	12.0676(8)
α/°	79.9490(10)
β/°	66.8730(10)
γ/°	77.9780(10)
Volume/Å^3	1102.16(13)
Z	2
ρ_{calc}g/cm^3	1.587
μ/mm^{-1}	1.059
F(000)	532.0
Crystal size/mm^3	0.45 × 0.43 × 0.19
Radiation	MoKα (λ = 0.71073)
2Θ range for data collection/°	4.096 to 66.034
Index ranges	-14 ≤ h ≤ 15, -15 ≤ k ≤ 15, -18 ≤ l ≤ 17
Reflections collected	14954
Independent reflections	7733 [R_{int} = 0.0135, R_{sigma} = 0.0190]
Data/restraints/parameters	7733/0/323
Goodness-of-fit on F^2	1.030
Final R indexes [I>=2σ (I)]	R_1 = 0.0250, wR_2 = 0.0701
Final R indexes [all data]	R_1 = 0.0265, wR_2 = 0.0710
Largest diff. peak/hole / e Å^{-3}	0.55/-0.34

Table S8: Bond Lengths for **[$Ph_2B(3\text{-}CF_3)Pz)_2$]Cu($C_2H_4$)**.

Atom	Atom	Length/Å	Atom	Atom	Length/Å
Cu	N2	1.9745(8)	C2	C3	1.3887(14)
Cu	N4	1.9795(8)	C5	C6	1.3929(14)
Cu	C21	2.0123(11)	C5	C8	1.4881(13)

Table S8: Bond Lengths for **[$Ph_2B(3\text{-}CF_3)Pz)_2$]Cu($C_2H_4$)**.

Atom	Atom	Length/Å	Atom	Atom	Length/Å
Cu	C22	2.0184(11)	C6	C7	1.3883(13)
F1	C4	1.3388(12)	C9	C10	1.3993(14)
F2	C4	1.3432(13)	C9	C14	1.4079(14)
F3	C4	1.3375(12)	C9	B	1.6205(14)
F4	C8	1.3398(12)	C10	C11	1.4032(16)
F5	C8	1.3448(13)	C11	C12	1.386(2)
F6	C8	1.3393(12)	C12	C13	1.387(2)
N1	N2	1.3623(11)	C13	C14	1.3898(16)
N1	C3	1.3473(12)	C15	C16	1.4009(13)
N1	B	1.5935(13)	C15	C20	1.4078(14)
N2	C1	1.3412(12)	C15	B	1.6170(14)
N3	N4	1.3648(11)	C16	C17	1.3979(15)
N3	C7	1.3466(12)	C17	C18	1.3892(17)
N3	B	1.5779(13)	C18	C19	1.3920(17)
N4	C5	1.3409(12)	C19	C20	1.3952(14)
C1	C2	1.3937(14)	C21	C22	1.3750(17)
C1	C4	1.4866(14)			

Table S9: Bond Angles for **[$Ph_2B(3\text{-}CF_3)Pz)_2$]Cu($C_2H_4$)**.

Atom	Atom	Atom	Angle/°	Atom	Atom	Atom	Angle/°
N2	Cu	N4	95.12(3)	C7	C6	C5	103.86(8)
N2	Cu	C21	113.95(4)	N3	C7	C6	108.93(9)
N2	Cu	C22	153.68(4)	F4	C8	F5	106.31(9)
N4	Cu	C21	150.78(4)	F4	C8	C5	112.89(8)
N4	Cu	C22	111.18(4)	F5	C8	C5	112.89(9)
C21	Cu	C22	39.89(5)	F6	C8	F4	107.55(9)
N2	N1	B	119.24(7)	F6	C8	F5	106.74(9)
C3	N1	N2	109.68(8)	F6	C8	C5	110.13(9)

Table S9: Bond Angles for $[Ph_2B(3\text{-}CF_3)Pz)_2]Cu(C_2H_4)$.

Atom	Atom	Atom	Angle/°	Atom	Atom	Atom	Angle/°
C3	N1	B	130.31(8)	C10	C9	C14	116.82(9)
N1	N2	Cu	116.35(6)	C10	C9	B	124.10(9)
C1	N2	Cu	136.87(7)	C14	C9	B	119.06(9)
C1	N2	N1	106.16(8)	C9	C10	C11	121.60(10)
N4	N3	B	120.33(8)	C12	C11	C10	119.97(11)
C7	N3	N4	109.78(8)	C11	C12	C13	119.57(11)
C7	N3	B	129.57(8)	C12	C13	C14	120.23(12)
N3	N4	Cu	116.47(6)	C13	C14	C9	121.76(11)
C5	N4	Cu	136.81(7)	C16	C15	C20	116.70(9)
C5	N4	N3	105.97(8)	C16	C15	B	121.72(9)
N2	C1	C2	111.35(9)	C20	C15	B	121.56(8)
N2	C1	C4	120.70(9)	C17	C16	C15	121.83(10)
C2	C1	C4	127.93(9)	C18	C17	C16	120.07(10)
C3	C2	C1	103.79(9)	C17	C18	C19	119.59(10)
N1	C3	C2	109.02(9)	C18	C19	C20	119.81(10)
F1	C4	F2	106.11(9)	C19	C20	C15	121.98(10)
F1	C4	C1	113.00(8)	C22	C21	Cu	70.30(6)
F2	C4	C1	112.63(9)	C21	C22	Cu	69.81(6)
F3	C4	F1	107.02(9)	N1	B	C9	105.49(7)
F3	C4	F2	106.49(9)	N1	B	C15	109.00(8)
F3	C4	C1	111.16(8)	N3	B	N1	106.25(7)
N4	C5	C6	111.45(8)	N3	B	C9	109.52(8)
N4	C5	C8	121.09(9)	N3	B	C15	110.01(8)
C6	C5	C8	127.46(9)	C15	B	C9	116.04(8)

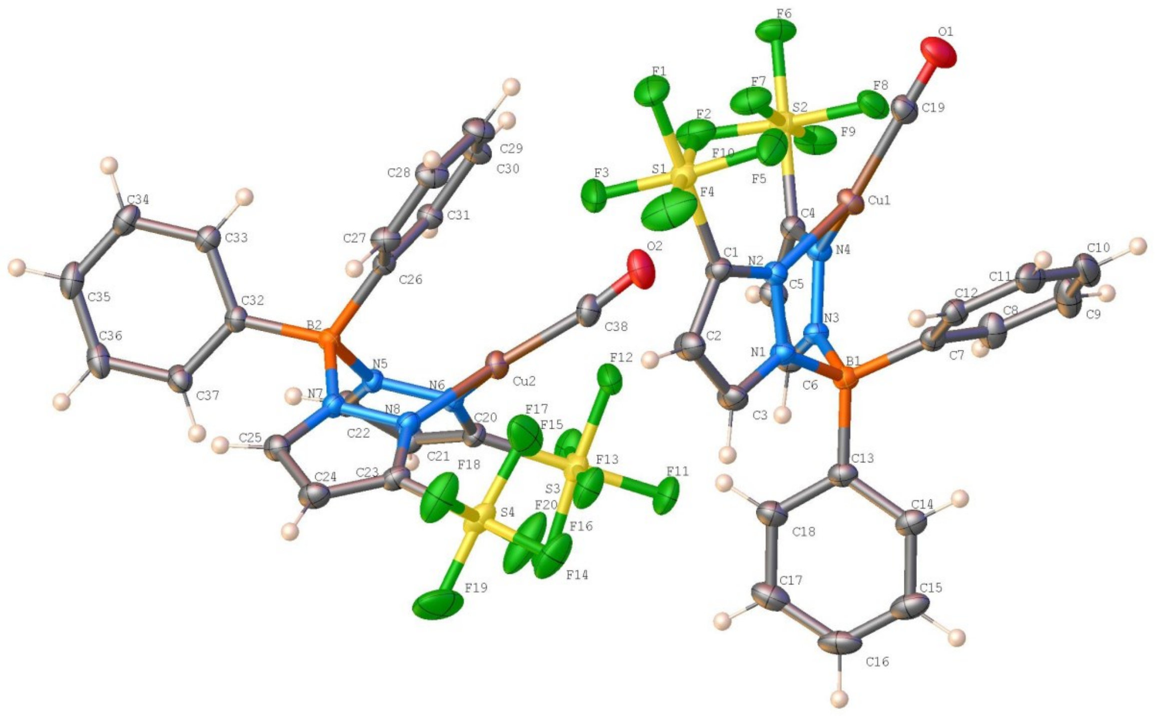

Figure S38: Molecular structure and atom labelling scheme of **[$Ph_2B(3\text{-}SF_5)Pz_2$]Cu(CO).**

Table S10: Crystal data and structure refinement for **[$Ph_2B(3\text{-}SF_5)Pz_2$]Cu(CO)**.

Identification code	HRD9_0m_a
Empirical formula	$C_{19}H_{14}BCuF_{10}N_4OS_2$
Formula weight	642.81
Temperature/K	100.0
Crystal system	triclinic
Space group	P-1
a/Å	11.5023(4)
b/Å	14.7295(5)
c/Å	15.6519(6)

α/°	80.195(2)
β/°	74.824(2)
γ/°	68.378(2)
Volume/Å^3	2371.16(15)
Z	4
ρ_{calc}g/cm^3	1.801
μ/mm^{-1}	1.198
F(000)	1280.0
Crystal size/mm^3	0.225 × 0.21 × 0.055
Radiation	Mo Kα (λ = 0.71073)
2Θ range for data collection/°	5.412 to 64.062
Index ranges	-17 ≤ h ≤ 17, -21 ≤ k ≤ 21, -23 ≤ l ≤ 23
Reflections collected	43095
Independent reflections	16208 [R_{int} = 0.0282, R_{sigma} = 0.0339]
Data/restraints/parameters	16208/0/686
Goodness-of-fit on F^2	1.091
Final R indexes [I>=2σ (I)]	R_1 = 0.0384, wR_2 = 0.0822
Final R indexes [all data]	R_1 = 0.0601, wR_2 = 0.0959
Largest diff. peak/hole / e Å^{-3}	0.59/-0.64

Table S11: Bond Lengths for **[$Ph_2B(3\text{-}SF_5)Pz_2$]Cu(CO)**.

Atom	Atom	Length/Å	Atom	Atom	Length/Å
Cu1	N2	2.0054(15)	Cu2	N8	2.0094(16)
Cu1	N4	1.9910(15)	Cu2	C26	2.5101(17)
Cu1	C19	1.803(2)	Cu2	C38	1.807(2)
S1	F1	1.5767(13)	S3	F11	1.5788(13)
S1	F2	1.5736(14)	S3	F12	1.5816(14)
S1	F3	1.5756(15)	S3	F13	1.5810(14)
S1	F4	1.5680(15)	S3	F14	1.5772(14)
S1	F5	1.5821(15)	S3	F15	1.5800(14)

Table S11: Bond Lengths for **[$Ph_2B(3\text{-}SF_5)Pz_2$]Cu(CO)**.

Atom	Atom	Length/Å	Atom	Atom	Length/Å
S1	C1	1.7816(19)	S3	C20	1.7818(18)
S2	F6	1.5822(13)	S4	F16	1.5807(14)
S2	F7	1.5808(13)	S4	F17	1.5708(17)
S2	F8	1.5786(14)	S4	F18	1.5703(15)
S2	F9	1.5737(13)	S4	F19	1.5713(18)
S2	F10	1.5795(14)	S4	F20	1.5699(16)
S2	C4	1.7870(18)	S4	C23	1.786(2)
O1	C19	1.120(2)	O2	C38	1.121(3)
N1	N2	1.358(2)	N5	N6	1.360(2)
N1	C3	1.349(2)	N5	C22	1.347(2)
N1	B1	1.584(3)	N5	B2	1.590(2)
N2	C1	1.331(2)	N6	C20	1.329(2)
N3	N4	1.359(2)	N7	N8	1.363(2)
N3	C6	1.346(2)	N7	C25	1.349(2)
N3	B1	1.598(3)	N7	B2	1.596(2)
N4	C4	1.332(2)	N8	C23	1.336(2)
C1	C2	1.389(3)	C20	C21	1.388(3)
C2	C3	1.379(3)	C21	C22	1.385(3)
C4	C5	1.384(3)	C23	C24	1.385(3)
C5	C6	1.378(3)	C24	C25	1.381(3)
C7	C8	1.408(3)	C26	C27	1.407(2)
C7	C12	1.404(3)	C26	C31	1.410(2)
C7	B1	1.617(3)	C26	B2	1.622(3)
C8	C9	1.384(3)	C27	C28	1.389(3)
C9	C10	1.391(4)	C28	C29	1.394(3)
C10	C11	1.382(3)	C29	C30	1.385(3)
C11	C12	1.398(3)	C30	C31	1.392(3)
C13	C14	1.395(3)	C32	C33	1.400(3)

Table S11: Bond Lengths for **[$Ph_2B(3\text{-}SF_5)Pz_2$]Cu(CO)**.

Atom	Atom	Length/Å	Atom	Atom	Length/Å
C13	C18	1.405(3)	C32	C37	1.405(3)
C13	B1	1.611(3)	C32	B2	1.607(3)
C14	C15	1.396(3)	C33	C34	1.396(3)
C15	C16	1.383(4)	C34	C35	1.380(3)
C16	C17	1.387(4)	C35	C36	1.389(3)
C17	C18	1.393(3)	C36	C37	1.390(3)
Cu2	N6	2.0154(15)			

Table S12: Bond Angles for **[$Ph_2B(3\text{-}SF_5)Pz_2$]Cu(CO)**.

Atom	Atom	Atom	Angle/˚	Atom	Atom	Atom	Angle/˚
N4	Cu1	N2	92.50(6)	C38	Cu2	N6	130.38(8)
C19	Cu1	N2	134.34(8)	C38	Cu2	N8	138.06(8)
C19	Cu1	N4	131.12(8)	C38	Cu2	C26	107.98(8)
F1	S1	F5	87.99(8)	F11	S3	F12	87.74(8)
F1	S1	C1	179.21(9)	F11	S3	F13	87.96(7)
F2	S1	F1	87.70(8)	F11	S3	F15	88.16(7)
F2	S1	F3	89.42(9)	F11	S3	C20	179.24(9)
F2	S1	F5	89.37(9)	F12	S3	C20	91.84(8)
F2	S1	C1	92.58(8)	F13	S3	F12	90.16(8)
F3	S1	F1	87.95(8)	F13	S3	C20	92.68(8)
F3	S1	F5	175.81(8)	F14	S3	F11	88.44(8)
F3	S1	C1	91.31(8)	F14	S3	F12	176.17(8)
F4	S1	F1	88.16(8)	F14	S3	F13	89.99(8)
F4	S1	F2	175.86(8)	F14	S3	F15	89.68(8)
F4	S1	F3	90.28(10)	F14	S3	C20	91.97(8)
F4	S1	F5	90.63(11)	F15	S3	F12	89.91(8)
F4	S1	C1	91.55(9)	F15	S3	F13	176.11(7)

Table S12: Bond Angles for **[$Ph_2B(3\text{-}SF_5)Pz_2$]Cu(CO)**.

Atom	Atom	Atom	Angle/˚	Atom	Atom	Atom	Angle/˚
F5	S1	C1	92.75(9)	F15	S3	C20	91.21(8)
F6	S2	C4	179.36(8)	F16	S4	C23	179.41(11)
F7	S2	F6	87.98(7)	F17	S4	F16	87.92(9)
F7	S2	C4	92.52(8)	F17	S4	F19	176.05(9)
F8	S2	F6	87.85(7)	F17	S4	C23	92.64(9)
F8	S2	F7	90.02(8)	F18	S4	F16	87.93(8)
F8	S2	F10	176.09(7)	F18	S4	F17	89.42(11)
F8	S2	C4	92.54(8)	F18	S4	F19	89.31(11)
F9	S2	F6	88.07(7)	F18	S4	C23	91.87(8)
F9	S2	F7	175.99(7)	F19	S4	F16	88.30(9)
F9	S2	F8	90.48(8)	F19	S4	C23	91.14(9)
F9	S2	F10	89.53(8)	F20	S4	F16	88.04(8)
F9	S2	C4	91.43(8)	F20	S4	F17	90.32(12)
F10	S2	F6	88.23(7)	F20	S4	F18	175.96(9)
F10	S2	F7	89.69(8)	F20	S4	F19	90.68(12)
F10	S2	C4	91.37(8)	F20	S4	C23	92.16(9)
N2	N1	B1	118.38(14)	N6	N5	B2	117.30(14)
C3	N1	N2	109.83(15)	C22	N5	N6	109.82(14)
C3	N1	B1	131.79(16)	C22	N5	B2	132.87(15)
N1	N2	Cu1	113.51(11)	N5	N6	Cu2	112.22(11)
C1	N2	Cu1	140.80(13)	C20	N6	Cu2	141.96(13)
C1	N2	N1	105.67(15)	C20	N6	N5	105.81(15)
N4	N3	B1	117.47(14)	N8	N7	B2	117.30(14)
C6	N3	N4	109.33(15)	C25	N7	N8	109.44(15)
C6	N3	B1	133.17(15)	C25	N7	B2	133.21(16)
N3	N4	Cu1	114.27(11)	N7	N8	Cu2	112.16(11)
C4	N4	Cu1	139.55(13)	C23	N8	Cu2	142.18(14)
C4	N4	N3	105.92(14)	C23	N8	N7	105.65(15)
N2	C1	S1	120.51(14)	N6	C20	S3	120.69(14)

Table S12: Bond Angles for **[$Ph_2B(3\text{-}SF_5)Pz_2$]Cu(CO)**.

Atom	Atom	Atom	Angle/°	Atom	Atom	Atom	Angle/°
N2	C1	C2	112.04(17)	N6	C20	C21	112.06(16)
C2	C1	S1	127.31(15)	C21	C20	S3	127.23(14)
C3	C2	C1	103.61(17)	C22	C21	C20	103.57(16)
N1	C3	C2	108.85(18)	N5	C22	C21	108.73(16)
N4	C4	S2	120.67(14)	N8	C23	S4	119.99(15)
N4	C4	C5	111.94(16)	N8	C23	C24	112.15(17)
C5	C4	S2	127.38(14)	C24	C23	S4	127.86(15)
C6	C5	C4	103.56(16)	C25	C24	C23	103.51(17)
N3	C6	C5	109.24(16)	N7	C25	C24	109.25(17)
C8	C7	B1	120.02(17)	C27	C26	Cu2	95.71(12)
C12	C7	C8	116.01(18)	C27	C26	C31	116.30(16)
C12	C7	B1	123.96(17)	C27	C26	B2	121.19(16)
C9	C8	C7	122.3(2)	C31	C26	Cu2	91.20(11)
C8	C9	C10	120.1(2)	C31	C26	B2	122.45(15)
C11	C10	C9	119.5(2)	B2	C26	Cu2	85.83(10)
C10	C11	C12	120.0(2)	C28	C27	C26	122.07(18)
C11	C12	C7	122.10(19)	C27	C28	C29	119.98(18)
C14	C13	C18	116.95(18)	C30	C29	C28	119.54(18)
C14	C13	B1	121.33(18)	C29	C30	C31	120.10(18)
C18	C13	B1	121.70(17)	C30	C31	C26	121.95(17)
C13	C14	C15	121.6(2)	C33	C32	C37	116.81(17)
C16	C15	C14	120.2(2)	C33	C32	B2	120.52(16)
C15	C16	C17	119.7(2)	C37	C32	B2	122.65(16)
C16	C17	C18	119.8(2)	C34	C33	C32	121.69(18)
C17	C18	C13	121.8(2)	C35	C34	C33	120.17(19)
O1	C19	Cu1	179.3(2)	C34	C35	C36	119.51(18)
N1	B1	N3	105.47(14)	C35	C36	C37	120.22(19)
N1	B1	C7	108.58(15)	C36	C37	C32	121.59(19)
N1	B1	C13	109.94(15)	O2	C38	Cu2	172.2(2)

Table S12: Bond Angles for **[$Ph_2B(3\text{-}SF_5)Pz_2$]Cu(CO)**.

**Atom**	**Atom**	**Atom**	**Angle/˚**	**Atom**	**Atom**	**Atom**	**Angle/˚**
N3	B1	C7	105.71(14)	N5	B2	N7	104.66(13)
N3	B1	C13	109.30(15)	N5	B2	C26	108.07(14)
C13	B1	C7	117.15(16)	N5	B2	C32	110.90(14)
N6	Cu2	C26	81.34(6)	N7	B2	C26	106.39(14)
N8	Cu2	N6	91.24(6)	N7	B2	C32	110.23(14)
N8	Cu2	C26	79.71(6)	C32	B2	C26	115.91(15)

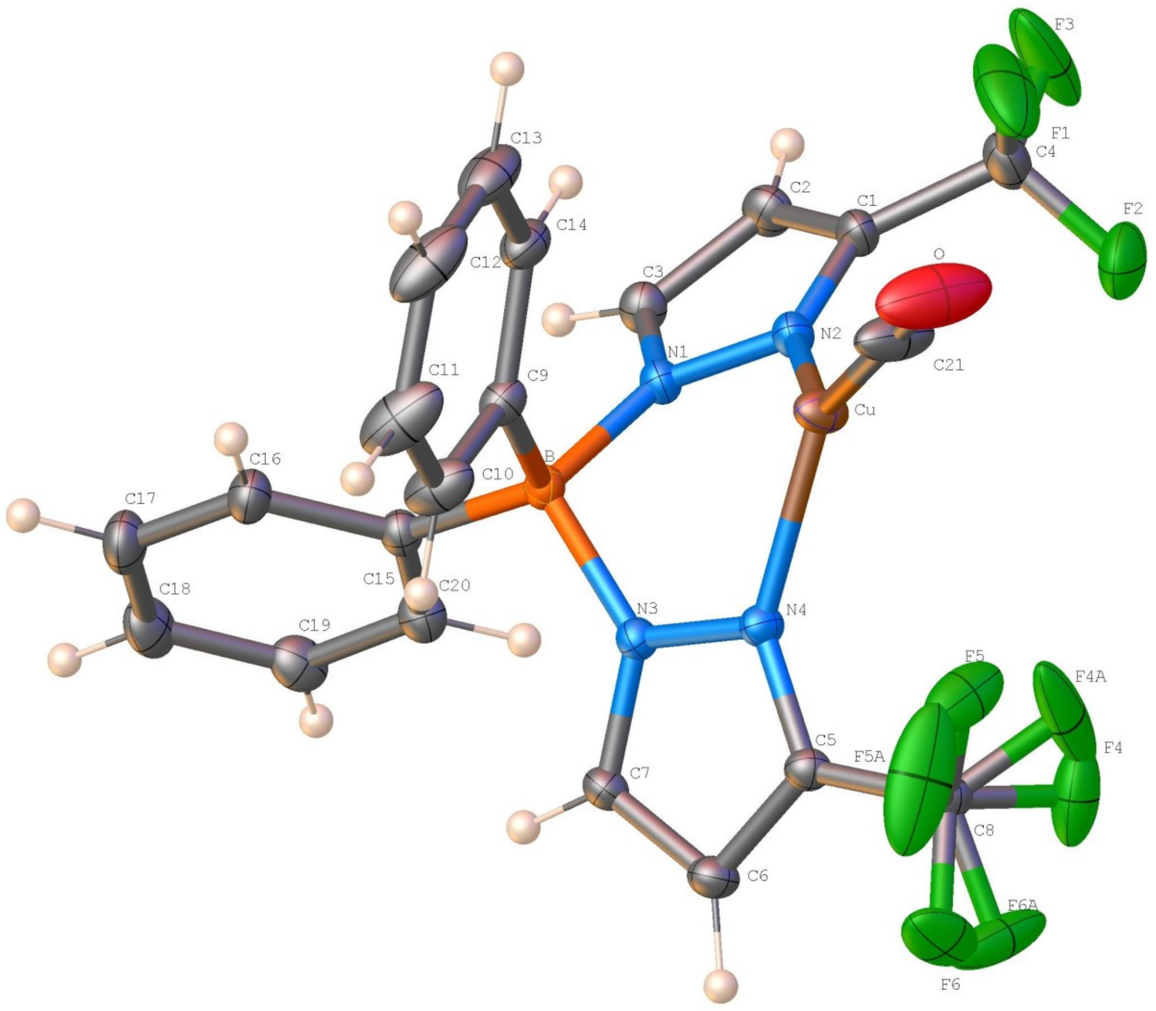

Figure S39: Molecular structure and atom labelling scheme of **$[Ph_2B(3\text{-}CF_3)Pz_2]Cu(CO)$.**

Table S13: Crystal data and structure refinement for **$[Ph_2B(3\text{-}CF_3)Pz_2]Cu(CO)$**.

Identification code	HRD8_0m_a
Empirical formula	$C_{21}H_{14}BCuF_6N_4O$
Formula weight	526.71
Temperature/K	100.0
Crystal system	triclinic

Space group	P-1
a/Å	10.1945(6)
b/Å	10.2497(6)
c/Å	12.0937(7)
α/°	114.940(2)
β/°	91.685(3)
γ/°	106.275(2)
Volume/Å^3	1084.00(11)
Z	2
ρ_{calc}g/cm^3	1.614
μ/mm^{-1}	1.081
F(000)	528.0
Crystal size/mm^3	0.4 × 0.25 × 0.2
Radiation	Mo Kα (λ = 0.71073)
2Θ range for data collection/°	4.474 to 66.282
Index ranges	-15 ≤ h ≤ 15, -15 ≤ k ≤ 15, -18 ≤ l ≤ 18
Reflections collected	20881
Independent reflections	8090 [R_{int} = 0.0157, R_{sigma} = 0.0184]
Data/restraints/parameters	8090/0/335
Goodness-of-fit on F^2	1.094
Final R indexes [I>=2σ (I)]	R_1 = 0.0355, wR_2 = 0.0863
Final R indexes [all data]	R_1 = 0.0412, wR_2 = 0.0918
Largest diff. peak/hole / e Å^{-3}	1.04/-0.87

Table S14: Bond Lengths for **[$Ph_2B(3\text{-}CF_3)Pz_2$]Cu(CO)**.

Atom	Atom	Length/Å	Atom	Atom	Length/Å
Cu	N2	1.9871(11)	C5	C6	1.3927(17)
Cu	N4	1.9838(10)	C5	C8	1.4879(17)
Cu	C21	1.8028(16)	C6	C7	1.3868(17)

Table S14: Bond Lengths for **[$Ph_2B(3\text{-}CF_3)Pz_2$]Cu(CO)**.

Atom	Atom	Length/Å	Atom	Atom	Length/Å
F1	C4	1.318(2)	C8	F4A	1.287(9)
F2	C4	1.3269(19)	C8	F5A	1.342(9)
F3	C4	1.3296(19)	C8	F6A	1.306(9)
F4	C8	1.331(2)	C9	C10	1.4047(19)
F5	C8	1.3167(19)	C9	C14	1.4013(18)
F6	C8	1.3241(19)	C9	B	1.6227(18)
O	C21	1.119(2)	C10	C11	1.390(2)
N1	N2	1.3590(14)	C11	C12	1.381(3)
N1	C3	1.3452(16)	C12	C13	1.385(3)
N1	B	1.5804(16)	C13	C14	1.4000(19)
N2	C1	1.3391(16)	C15	C16	1.3988(17)
N3	N4	1.3593(14)	C15	C20	1.4078(17)
N3	C7	1.3454(15)	C15	B	1.6078(18)
N3	B	1.5925(17)	C16	C17	1.3969(19)
N4	C5	1.3397(15)	C17	C18	1.385(2)
C1	C2	1.3929(19)	C18	C19	1.389(2)
C1	C4	1.4844(19)	C19	C20	1.3934(18)
C2	C3	1.3844(18)			

Table S15: Bond Angles for **[$Ph_2B(3\text{-}CF_3)Pz_2$]Cu(CO)**.

Atom	Atom	Atom	Angle/˚	Atom	Atom	Atom	Angle/˚
N4	Cu	N2	93.93(4)	F5	C8	F6	107.79(15)
C21	Cu	N2	137.21(6)	F5	C8	C5	113.28(12)
C21	Cu	N4	128.47(6)	F6	C8	F4	106.10(16)
N2	N1	B	119.38(9)	F6	C8	C5	110.53(13)
C3	N1	N2	109.90(10)	F4A	C8	C5	115.7(4)
C3	N1	B	130.01(10)	F4A	C8	F5A	105.4(10)
N1	N2	Cu	114.71(8)	F4A	C8	F6A	105.0(9)

Table S15: Bond Angles for **[$Ph_2B(3\text{-}CF_3)Pz_2$]Cu(CO)**.

Atom	Atom	Atom	Angle/˚	Atom	Atom	Atom	Angle/˚
C1	N2	Cu	138.75(9)	F5A	C8	C5	113.1(5)
C1	N2	N1	105.91(10)	F6A	C8	C5	115.1(4)
N4	N3	B	118.83(9)	F6A	C8	F5A	101.0(10)
C7	N3	N4	109.60(10)	C10	C9	B	119.77(11)
C7	N3	B	131.16(10)	C14	C9	C10	116.69(12)
N3	N4	Cu	114.98(7)	C14	C9	B	123.54(11)
C5	N4	Cu	138.59(8)	C11	C10	C9	121.79(15)
C5	N4	N3	106.42(9)	C12	C11	C10	120.33(16)
N2	C1	C2	111.49(11)	C11	C12	C13	119.55(14)
N2	C1	C4	120.28(12)	C12	C13	C14	120.05(15)
C2	C1	C4	128.23(12)	C13	C14	C9	121.56(14)
C3	C2	C1	103.72(11)	C16	C15	C20	116.77(11)
N1	C3	C2	108.97(11)	C16	C15	B	121.74(11)
F1	C4	F2	106.16(14)	C20	C15	B	121.47(10)
F1	C4	F3	108.29(16)	C17	C16	C15	121.68(13)
F1	C4	C1	113.06(13)	C18	C17	C16	120.08(13)
F2	C4	F3	105.91(14)	C17	C18	C19	119.85(12)
F2	C4	C1	112.91(13)	C18	C19	C20	119.66(13)
F3	C4	C1	110.13(13)	C19	C20	C15	121.94(12)
N4	C5	C6	111.04(11)	O	C21	Cu	176.4(2)
N4	C5	C8	120.94(11)	N1	B	N3	106.39(9)
C6	C5	C8	128.00(11)	N1	B	C9	108.09(10)
C7	C6	C5	103.96(10)	N1	B	C15	110.10(9)
N3	C7	C6	108.97(10)	N3	B	C9	106.00(9)
F4	C8	C5	112.18(12)	N3	B	C15	109.36(9)
F5	C8	F4	106.58(15)	C15	B	C9	116.39(10)

Steric maps of bis(pyrazolyl)borates in $[Ph_2B(3\text{-}(SF_5)Pz)_2]Cu(L)$ and $[Ph_2B(3\text{-}(CF_3)Pz)_2]Cu(L)$, L = C_2H_4 or CO

Computed using SambVca 2.1 for a sphere radius of 3.5 Å about the metal center, Bondi van der Waals radii scaled by a factor of 1.17, 0.10 Å mesh spacing, and including hydrogen atoms (difference in $\%V_{bur}$ value was negligible (≤0.1%) between inclusion or omission of H-atoms in these molecules during calculation).[10] Per SambVca protocol, metal-ethylene and metal-CO moieties were removed for the calculation of steric maps and $\%V_{bur}$ of bis(pyrazolyl)borate in the copper-ethylene and copper-CO complexes $[Ph_2B(3\text{-}(SF_5)Pz)_2]Cu(L)$ and $[Ph_2B(3\text{-}(CF_3)Pz)_2]Cu(L)$, L = C_2H_4 or CO.

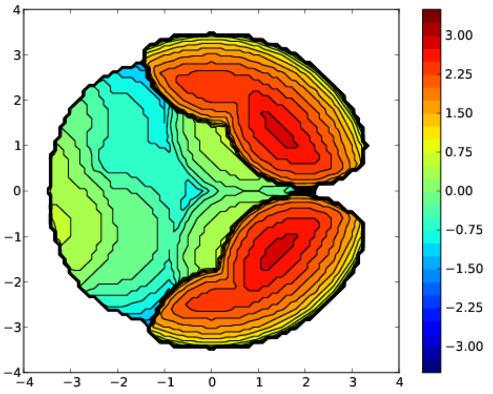

$[Ph_2B(3\text{-}(SF_5)Pz)_2]Cu(C_2H_4)$

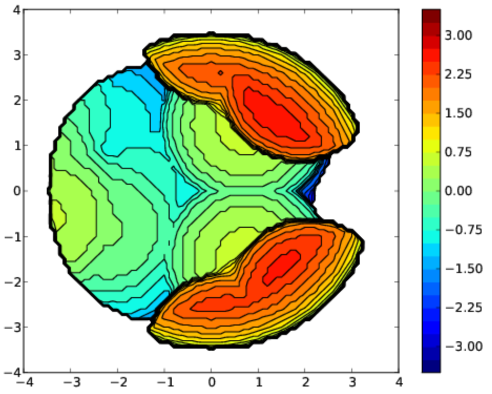

$[Ph_2B(3\text{-}(CF_3)Pz)_2]Cu(C_2H_4)$

Figure S40: Steric maps of bis(pyrazolyl)borate ligands in $[Ph_2B(3\text{-}(SF_5)Pz)_2]Cu(C_2H_4)$ (left) and $[Ph_2B(3\text{-}(CF_3)Pz)_2]Cu(C_2H_4)$ (right) based on X-ray data. The resulting % buried volume ($\%V_{bur}$) values are 65.6 (average of 66.1 and 65.0 for the two molecules in the asymmetric unit) and 59.5, respectively.

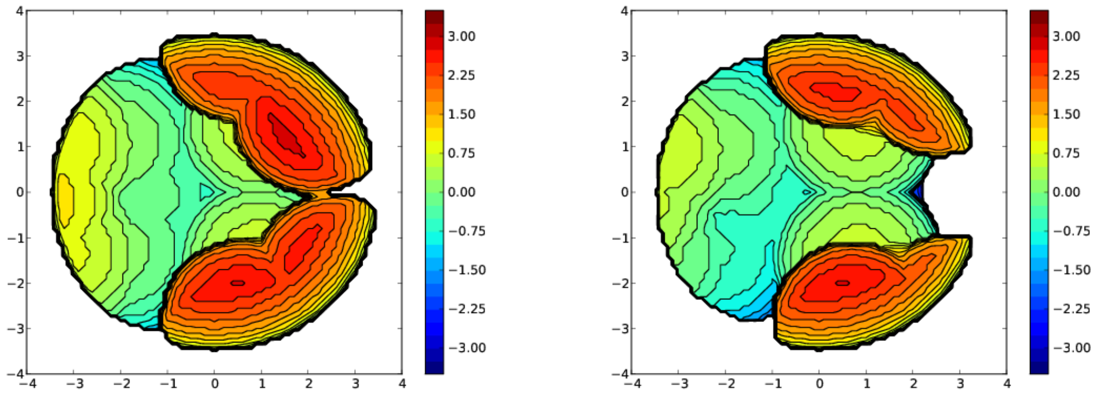

[Ph$_2$B(3-(SF$_5$)Pz)$_2$]Cu(CO) **[Ph$_2$B(3-(CF$_3$)Pz)$_2$]Cu(CO)**

Figure S41: Steric maps of bis(pyrazolyl)borate ligands in [Ph$_2$B(3-(SF$_5$)Pz)$_2$]Cu(CO) (left) and [Ph$_2$B(3-(CF$_3$)Pz)$_2$]Cu(CO) (right) based on X-ray data. The resulting % buried volume (%V$_{bur}$) values are 68.7 (average of 69.0 and 68.4 for the two molecules in the asymmetric unit) and 60.8 (fluorine atoms of one of the CF$_3$ groups of [Ph$_2$B(3-(CF$_3$)Pz)$_2$]Cu(CO) show positional disorder, and only the major occupancy fluorine atoms were utilized in the calculation to avoid inflated steric bulk), respectively. For comparison, inclusion of both disorder-parts in [Ph$_2$B(3-(CF$_3$)Pz)$_2$]Cu(CO) leads to a marginally higher %V$_{bur}$ value of 61.9.

Computational studies

All calculations were carried out by using relativistic DFT methods employing the ADF code[11] with the all-electron triple-ζ Slater basis set plus the double-polarization (STO-TZ2P) basis set in conjunction with the Becke-Perdew (BP86) functional[12] within the generalized gradient approximation (GGA). London dispersion corrections were taken into account via the pairwise Grimme (BP86-D3) approach.[13]Geometry optimizations were performed without any symmetry restrain via the analytical energy gradient method implemented by Versluis and Ziegler,[14] with energy convergence criteria set at 10^{-4} Hartree, gradient convergence criteria at 10^{-4} Hartree/Å, and radial convergence of 10^{-3} Å. Scalar relativistic effects were considered through the ZORA Hamiltonian.[15]

The interaction energy is further dissected into several chemically meaningful terms according to the Energy Decomposition Analysis (EDA) of Ziegler and Rauk,[16, 17]

$$\Delta E_{int} = \Delta E_{Pauli} + \Delta E_{elstat} + \Delta E_{orb} + \Delta E_{disp}$$

where ΔE_{Pauli} term involves the electron repulsion between occupied orbitals from the different fragments. ΔE_{elstat} and ΔE_{orb} are related to the stabilizing electrostatic and covalent character of the interaction, respectively. The contribution from dispersion interaction (ΔE_{disp}) is evaluated using the pairwise correction of Grimme (D3). Bonding analysis is given in terms of bonding contributions to ΔE_{orb} by using the Natural Orbitals for Chemical Valence extension of the EDA method (EDA-NOCV),[18] resulting in deformation densities accounting for the individual in- and out-flow of charges related to the bonding pattern.

Table S16: Calculated IR ῡ(C=C) stretching frequencies (cm^{-1}) and selected bond distances from optimized structures (Å)

Parameter	$[Ph_2B(3\text{-}(SF_5)Pz)_2]Cu(C_2H_4)$	$[Ph_2B(3\text{-}(CF_3)Pz)_2]Cu(C_2H_4)$	$[Ph_2B(3\text{-}(CH_3)Pz)_2]Cu(C_2H_4)$
ῡ(C=C)	1516.3	1513.5	1509.3
C=C	1.383	1.386	1.389
Cu-C	2.047	2.045	2.029
Cu-C	2.050	2.051	2.034

Table S17: Calculated proton affinities of $[Ph_2B(3\text{-}(R)Pz)_2]^{-}$ ligands with R= $-SF_5$, $-CF_3$, and $-CH_3$. Values in $kJ \cdot mol^{-1}$.

Parameter	$[Ph_2B(3\text{-}(SF_5)Pz)_2]^{-}$	$[Ph_2B(3\text{-}(CF_3)Pz)_2]^{-}$	$[Ph_2B(3\text{-}(CH_3)Pz)_2]^{-}$
proton affinity	936.5	944.1	1016.5

For comparison with experimental data, calculated and experimentally determined proton affinities for $3,5\text{-}(CH_3)_2PzH$ are 967.2 and 933.5 $kJ \cdot mol^{-1}$ respectively.[19]

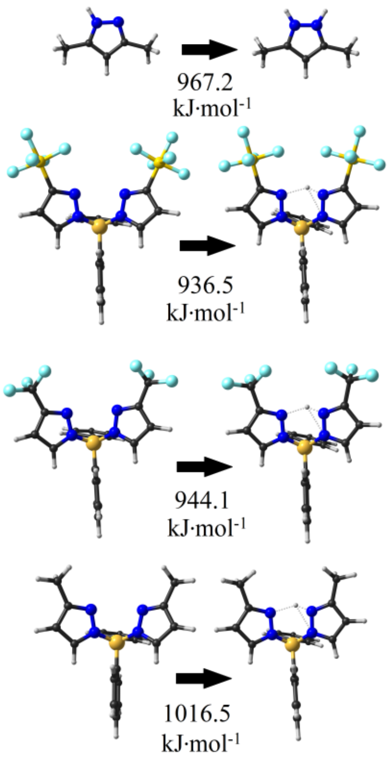

From top to bottom, the figures represent:

$3,5\text{-}(CH_3)_2PzH + H^+ \rightarrow [3,5\text{-}(CH_3)_2PzH_2]^+$

$[Ph_2B(3\text{-}(SF_5)Pz)_2]^{-} + H^+ \rightarrow [Ph_2B(3\text{-}(SF_5)Pz)_2]H$

$[Ph_2B(3\text{-}(CF_3)Pz)_2]^{-} + H^+ \rightarrow [Ph_2B(3\text{-}(CF_3)Pz)_2]H$

$[Ph_2B(3\text{-}(CH_3)Pz)_2]^{-} + H^+ \rightarrow [Ph_2B(3\text{-}(CH_3)Pz)_2]H$

Figure 42. Proton affinities (PA) were calculated for equations given above in which proton affinity is defined as the negative of the difference in energy between the protonated and non-protonated forms for hypothetical gas-phase protonation reactions (PA = - E_{PA}),

where $E_{PA} = E\ (protonated\ acceptor) - [\ E(acceptor) + E(H^+)]$

Table S18. Energy decomposition analyses for the C_2H_4-Cu interaction, for different $[Ph_2B(3\text{-}(R)Pz)_2]Cu(C_2H_4)$ complexes, in which R= -SF_5, -CF_3, and -CH_3. Values in kcal·mol^{-1}. In addition the contribution from both π_2^*-backbonding and σ-donation is given accounting for π_2*-C_2H_4 ← Cu and π_1-C_2H_4 → Cu bonding schemes, respectively. In addition, charge distribution analysis (*q*) obtained from Hirshfeld charge analysis is given.

Parameter	$[Ph_2B(3\text{-}(SF_5)Pz)_2]Cu(C_2H_4)$		$[Ph_2B(3\text{-}(CF_3)Pz)_2]Cu(C_2H_4)$		$[Ph_2B(3\text{-}(CH_3)Pz)_2]Cu(C_2H_4)$	
ΔE_{int}	-44.9		-45.9		-45.2	
ΔE_{Pauli}	114.0		112.5		125.4	
ΔE_{disp}	-6.5	4.1%[a]	-5.6	3.5%[a]	-5.3	3.1%[a]
ΔE_{elstat}	-95.2	59.9%[a]	-95.0	59.9%[a]	-102.1	59.9%[a]
ΔE_{orb}	-57.2	36.0%[a]	-57.9	36.6%[a]	-63.2	37.1%[a]
π_1-C_2H_4 → Cu	-17.1	29.9%[b]	-16.5	28.5%[b]	-15.5	24.5%[b]
π_2*-C_2H_4 ← Cu	-31.4	54.9%[b]	-33.5	57.9%[b]	-40.2	63.7%[b]
ΔE_{orb}^{rest}	-8.7		-7.8		-7.5	
[c]Popul. π_1	1.82		1.83		1.85	
[c]Popul. π_2*	0.22		0.27		0.30	
*q*L	-0.28		-0.28		-0.24	
*q*Cu	0.33		0.34		0.34	
q(C_2H_4)	-0.05		-0.06		-0.10	

[a]Percentage contribution to the total attractive interactions ΔE_{elstat}+ ΔE_{orb} + ΔE_{disp}.

[b]Percentage contribution to the total orbital interactions ΔE_{orb}.

[c]Polulation in a.u. for theylene π_1 and π_2* orbitals in the resulting complex.

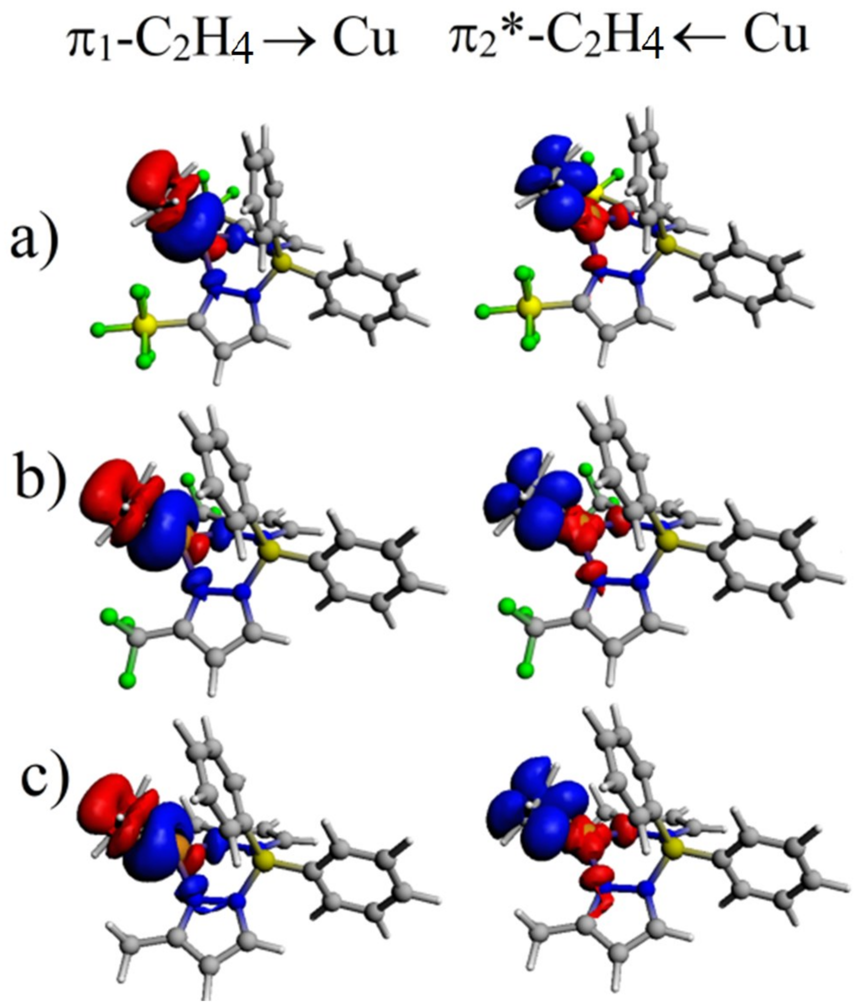

Figure S43: Selected deformation densities from the NOCV-EDA analysis, for **[$Ph_2B(3\text{-}(CF_3)Pz)_2$]$Cu(C_2H_4)$** (**a**), **[$Ph_2B(3\text{-}(SF_5)Pz)_2$]$Cu(C_2H_4)$** (**b**), and **[$Ph_2B(3\text{-}(CH_3)Pz)_2$]$Cu(C_2H_4)$** (**c**), accounting for σ-donation (left) and π-backbonding (right) in the formation of ethylene-copper complexes. Charge flow from red to blue.

Table S19: Energy decomposition analyses for the C_2H_4-Cu and C_2H_4-Ni interaction for isoelectronic [$H_2B(3-(CH_3)Pz)_2$]Cu(C_2H_4) (**Cu-BH₂**) and [$H_2C(3-(CH_3)Pz)_2$]Ni(C_2H_4) (**Ni-CH₂**) complexes. Values in kcal·mol^{-1}. In addition, the contribution from both π_2^*-backbonding and σ-donation is given accounting for π_2^*-C_2H_4 ← Cu/Ni and π_1-C_2H_4 → Cu/Ni bonding schemes, respectively. In addition, charge distribution analysis (q) obtained from Hirshfeld charge analysis (where L = [$H_2B(3-(CH_3)Pz)_2$] or [$H_2C(3-(CH_3)Pz)_2$] fragment) and calculated ῡ(C=C) (in cm^{-1}) are also given.

This calculation was performed to compare the π-backbonding and σ-donation components of closely related, Cu(I) and Ni(0) systems in their ethylene complexes.

Parameter	[$H_2B(3-(CH_3)Pz)_2$]Cu(C_2H_4)		[$H_2C(3-(CH_3)Pz)_2$]Ni(C_2H_4)	
ΔE_{int}	-44.1		-78.6	
ΔE_{Pauli}	123.1		173.4	
ΔE_{disp}	-7.2	4.3%[a]	-7.4	2.9%[a]
ΔE_{elstat}	-98.7	59.1%[a]	-137.2	54.4%[a]
ΔE_{orb}	-61.2	36.6%[a]	-107.5	42.7%[a]
π_1-C_2H_4 → Cu/Ni	-15.6	25.5%[b]	-13.6	12.7%[b]
π_2^*-C_2H_4 ← Cu/Ni	-39.0	63.6%[b]	-86.8	80.7%[b]
ΔE_{orb}^{rest}	-6.7	10.9%[b]	-7.1	6.6%[b]
qL	-0.28		0.12	
qCu/qNi	0.36		0.17	
q(C_2H_4)	-0.08		-0.29	
ῡ(C=C) Calc.	1513.9		1458.3	

[a]Percentage contribution to the total attractive interactions $\Delta E_{elstat} + \Delta E_{orb} + \Delta E_{disp}$.

[b]Percentage contribution to the total orbital interactions ΔE_{orb}.

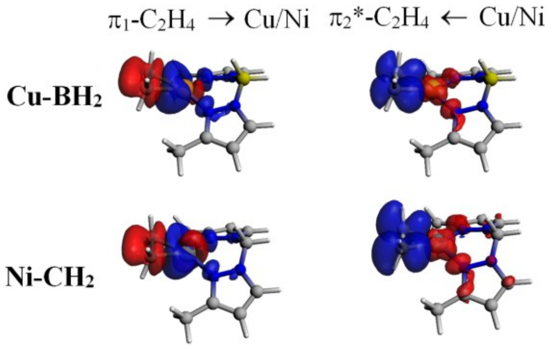

Figure S44: Selected deformation densities from the NOCV-EDA analysis for C_2H_4-Cu and C_2H_4-Ni interaction for the isoelectronic [$H_2B(3-(CH_3)Pz)_2$]Cu(C_2H_4) (**Cu-BH₂**) and [$H_2C(3-(CH_3)Pz)_2$]Ni(C_2H_4) (**Ni-CH₂**) complexes. Charge flow from red to blue.

Table S20: Energy decomposition analyses for the Cu-CO interaction, for different $[Ph_2B(3\text{-}(R)Pz)_2]Cu(CO)$ complexes, with R= $-SF_5$, $-CF_3$, and $-CH_3$. Values in kcal·mol^{-1}. In addition the contribution from both $\parallel 2\pi^*$- and $\perp 2\pi^*$-backbonding, and $3\sigma \rightarrow Cu$ donation are given. In addition, charge distribution analysis (q) obtained from Hirshfeld charge analysis (where L = $[Ph_2B(3\text{-}(R)Pz)_2]$ fragment) and calculated $\bar{\upsilon}(CO)$ (in cm^{-1}) are also given.

Parameter	$[Ph_2B(3\text{-}(SF_5)Pz)_2]$ Cu(CO)		$[Ph_2B(3\text{-}(CF_3)Pz)_2]$ Cu(CO)		$[Ph_2B(3\text{-}(CH_3)Pz)_2]$ Cu(CO)	
ΔE_{int}	-39.9		-39.8		-40.9	
ΔE_{Pauli}	116.1		118.6		125.2	
ΔE_{disp}	-3.9	2.5%[a]	-3.2	2.0%[a]	-2.7	1.6%[a]
ΔE_{elstat}	-95.6	61.2%[a]	-97.2	61.3%[a]	-100.4	60.5%[a]
ΔE_{orb}	-56.6	36.3%[a]	-58.1	36.7%[a]	-62.9	37.9%[a]
3σ-CO $\rightarrow$ Cu	-14.6	25.8%[b]	-14.1	24.3%[b]	-14.2	22.6%[b]
$\parallel 2\pi^*$-CO $\leftarrow$ Cu	-18.3	32.4%[b]	-19.7	33.9%[b]	-23.6	37.5%[b]
$\perp 2\pi^*$-CO $\leftarrow$ Cu	-13.1	23.2%[b]	-13.8	23.7%[b]	-15.1	24.0%[b]
ΔE_{orb}^{rest}	-10.6	18.6%[b]	-10.5	18.1%[b]	-10.0	15.9%[b]
qL	-0.21		-0.21		-0.20	
qCu	0.31		0.32		0.34	
q(CO)	-0.10		-0.11		-0.14	
$\bar{\upsilon}$(CO) Calc.	2110.0		2099.0		2080.3	
$\bar{\upsilon}$(CO) Exp.	2121		2117			

[a]Percentage contribution to the total attractive interactions $\Delta E_{elstat} + \Delta E_{orb} + \Delta E_{disp}$.

[b]Percentage contribution to the total orbital interactions ΔE_{orb}.

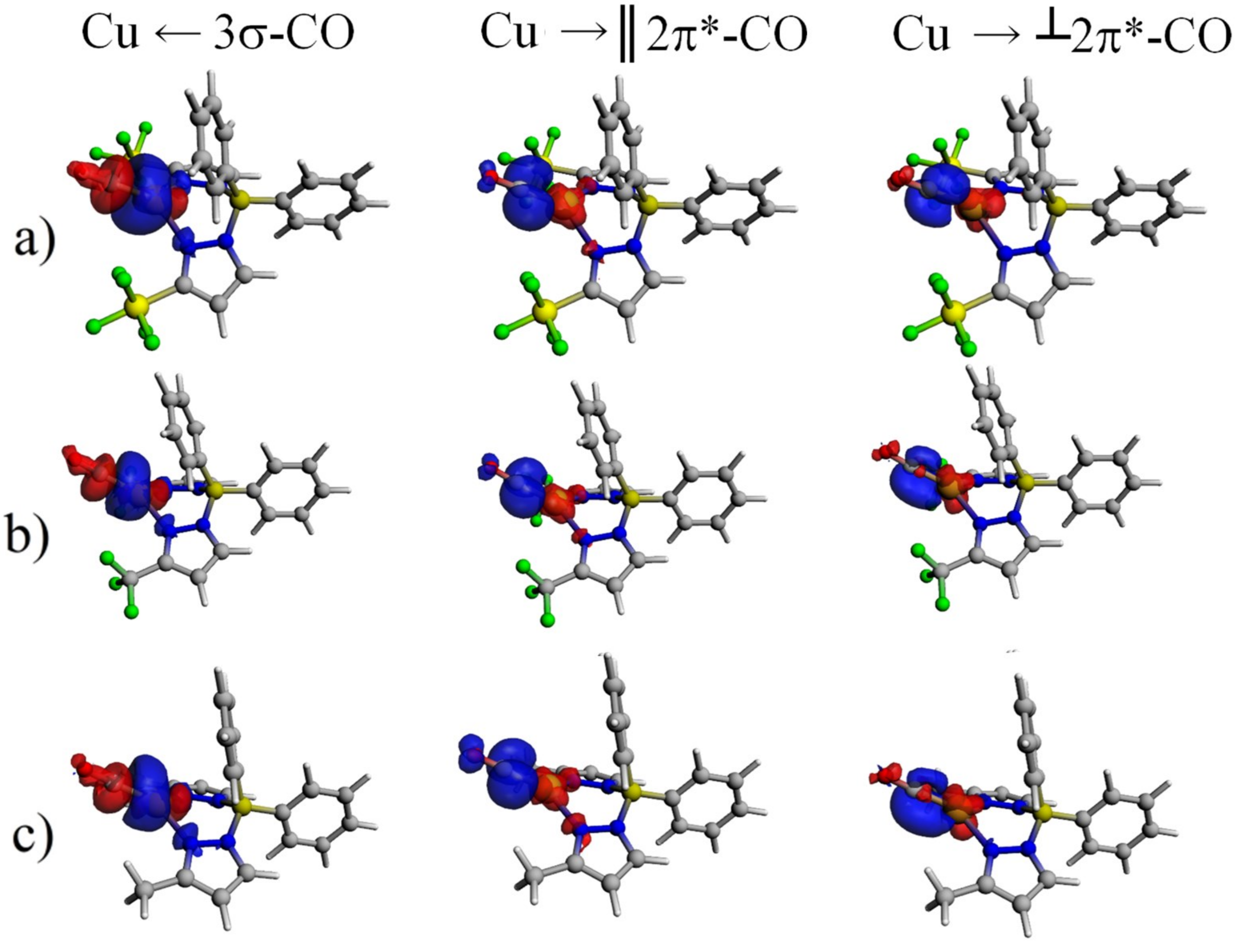

Figure S45: Selected deformation densities from the NOCV-EDA analysis, for $[Ph_2B(3\text{-}(SF_5)Pz)_2]Cu(CO)$ (**a**), $[Ph_2B(3\text{-}(CF_3)Pz)_2]Cu(CO)$ (**b**), and $[Ph_2B(3\text{-}(CH_3)Pz)_2]Cu(CO)$ (**c**) accounting for σ-donation, and perpendicular and parallel in relation to the Cu-N_2 plane π-backbonding in the formation of copper-CO complexes. Charge flow from red to blue.

Thermochemical parameters for C_2H_4 replacement by CO at 298 K. values in kcal·mol^{-1}.

$[Ph_2B(3\text{-}(SF_5)Pz)_2]Cu(C_2H_4) + CO \rightarrow [Ph_2B(3\text{-}(SF_5)Pz)_2]Cu(CO) + C_2H_4$

ΔG^{298K}	1.30
ΔH	1.59
$T\Delta S$	0.29

$[Ph_2B(3\text{-}(CF_3)Pz)_2]Cu(C_2H_4) + CO \rightarrow [Ph_2B(3\text{-}(CF_3)Pz)_2]Cu(CO) + C_2H_4$

ΔG^{298K}	0.59
ΔH	3.47
$T\Delta S$	2.87

References

1. S. Hyde, J. Veliks, B. Liégault, D. Grassi, M. Taillefer and V. Gouverneur, *Angewandte Chemie International Edition*, 2016, **55**, 3785-3789.
2. A. J. Lupinetti, S. H. Strauss and G. Frenking, in *Progress in Inorganic Chemistry*, 2001, DOI: https://doi.org/10.1002/9780470166512.ch1, pp. 1-112.
3. C. W. Tullock, D. D. Coffman and E. L. Muetterties, *Journal of the American Chemical Society*, 1964, **86**, 357-361.
4. J. A. Flores, V. Badarinarayana, S. Singh, C. J. Lovely and H. V. R. Dias, *Dalton Transactions*, 2009, DOI: 10.1039/B911981G, 7648-7652.
5. P. K. Mykhailiuk, S. Afonin, A. S. Ulrich and I. V. Komarov, *Synthesis*, 2008, **2008**, 1757-1760.
6. L. Krause, R. Herbst-Irmer, G. M. Sheldrick and D. Stalke, *Journal of Applied Crystallography*, 2015, **48**, 3-10.
7. G. Sheldrick, *Acta Crystallographica Section A*, 2015, **71**, 3-8.
8. G. Sheldrick, *Acta Crystallographica Section C*, 2015, **71**, 3-8.
9. O. V. Dolomanov, L. J. Bourhis, R. J. Gildea, J. A. K. Howard and H. Puschmann, *Journal of Applied Crystallography*, 2009, **42**, 339-341.
10. L. Falivene, Z. Cao, A. Petta, L. Serra, A. Poater, R. Oliva, V. Scarano and L. Cavallo, *Nature Chemistry*, 2019, **11**, 872-879.
11. Amsterdam Density Functional (ADF 2019) Code, Theoretical Chemistry, Vrije Universiteit, Amsterdam, The Netherlands, http://www.scm.com.
12. A. D. Becke, *Physical Review A*, 1988, **38**, 3098-3100.
13. S. Grimme, *WIREs Computational Molecular Science*, 2011, **1**, 211-228.
14. L. Versluis and T. Ziegler, *The Journal of Chemical Physics*, 1988, **88**, 322-328.
15. E. v. Lenthe, E. J. Baerends and J. G. Snijders, *The Journal of Chemical Physics*, 1994, **101**, 9783-9792.
16. M. v. Hopffgarten and G. Frenking, *WIREs Computational Molecular Science*, 2012, **2**, 43-62.
17. T. Ziegler and A. Rauk, *Theoretica chimica acta*, 1977, **46**, 1-10.
18. M. P. Mitoraj, A. Michalak and T. Ziegler, *Journal of Chemical Theory and Computation*, 2009, **5**, 962-975.
19. E. P. L. Hunter and S. G. Lias, *Journal of Physical and Chemical Reference Data*, 1998, **27**, 413-656.
